# Supplementary material for: A Dynamical Framework for the All-or-None G1/S Transition
Source: Cell Syst. 2016 Jan 27;2(1):27–37. doi: 10.1016/j.cels.2016.01.001 (PMC4802413; doi:10.1016/j.cels.2016.01.001)
Supplement: Document S1. Supplemental Experimental Procedures and Figures S1–S6 [file mmc1.pdf]

Cell Systems
Supplemental Information

A Dynamical Framework for the All-or-None G1/S Transition

Alexis R. Barr, Frank S. Heldt, Tongli Zhang, Chris Bakal, and Béla Novák

Inventory of Supplemental Information

Figure S1: Validation of GFP-tagged p27^{Kip1}, CyclinE1, and CyclinA2 constructs (related to Figure 1).

Figure S2: Validation of Cdk2 activity sensor, CDK2L-GFP (related to Figure 1).

Figure S3: Dynamics of G1/S regulators in unperturbed cells and upon Cdk2 inhibition (related to Figure 2).

Figure S4: siRNA-mediated depletion of CyclinE1/2 and CyclinA2 (related to Figures 3 and 4, respectively).

Figure S5: siRNA-mediated depletion of Emi1 (related to Figure 5).

Figure S6: Inhibition of Cdk2 and siRNA-mediated depletion of Emi1 (related to Figure 5).

Supplemental Experimental Procedures

Movie S1: p27^{Kip1}-GFP/LSS2-mKate PCNA expressing HeLa cells (related to Figure 1A).
Movie S2: CyclinE1-GFP/LSS2-mKate PCNA expressing HeLa cells (related to Figure 1B).
Movie S3: CyclinA2-GFP/LSS2-mKate PCNA expressing HeLa cells (related to Figure 1C).
Movie S4: CDK2L-GFP/LSS2-mKate PCNA expressing HeLa cells (related to Figure 1D).
Movie S5: GFP-PCNA expressing HeLa cells treated with Control siRNA (related to Figure S6B).
Movie S6: GFP-PCNA expressing HeLa cells treated with Emi1 siRNA (related to Figure S6B).

Supplemental References

Supplemental Figures

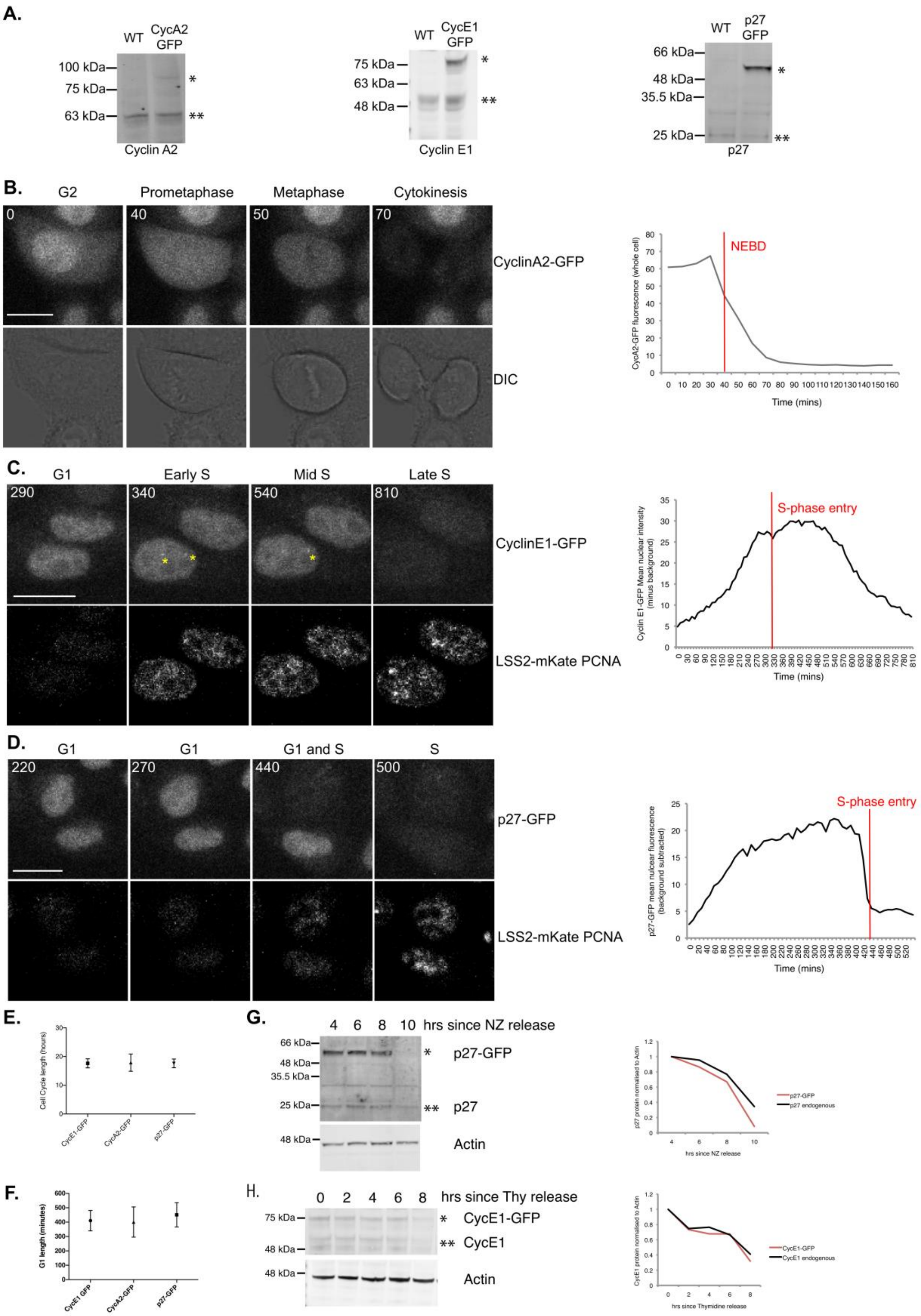

Figure S1: Validation of GFP-tagged p27^{Kip1}, CyclinE1, and CyclinA2 constructs (related to Figure 1). **A.** Western blots of HeLa clonal cell lines expressing GFP-tagged BAC reporters generated in this study. From left to right: CyclinA2-GFP, CyclinE1-GFP and p27^{Kip1}-GFP. In all three Western blots, wild-type (WT) HeLa lysate is shown on the left and the clonal cell line on the right. The lower bands (marked by double asterisks) are the endogenous proteins and the upper bands (marked by a single asterisk) are the GFP-tagged proteins expressed from the BAC. **B.** CyclinA2-GFP mimics endogenous CyclinA2 localisation and expression: it is stable during S and G2 phases but degraded during mitosis (den Elzen and Pines, 2001). NEBD = nuclear envelope breakdown. Scale bar is 10 μm. Graph represents CyclinA2-GFP expression in a single cell. Relative time is shown in minutes on each image. **C.** CyclinE1-GFP mimics endogenous CyclinE1 localisation and expression in the nucleus and to Cajal bodies (marked by yellow asterisks; (Liu et al., 2000)). CyclinE1-GFP accumulates during G1, and begins to be degraded after cells enter S-phase. CyclinE1-GFP is degraded before the end of S-phase, as reported for endogenous CyclinE1 (Dulic et al., 1992; Koff et al., 1992). Scale bar is 10 μm. Graph represents CyclinE1-GFP expression in a single cell. Relative time is shown in minutes on each image. **D.** p27^{Kip1}-GFP mimics endogenous p27^{Kip1} localisation and expression in the nucleus. p27^{Kip1}-GFP accumulates during G1 and is degraded at S-phase entry, as reported for endogenous p27^{Kip1} (Carrano et al., 1999). Scale bar is 10 μm. Graph represents p27^{Kip1}-GFP expression in a single cell. Relative time is shown in minutes on each image. **E.** Cell cycle length is not significantly different (one-way ANOVA followed by Tukey's multiple comparison test, $p<0.05$) between LSS2-mKate-PCNA sensor expressing cell lines used in this study. Mean +/- standard deviation (STD) is shown. Number of cells analysed: CyclinE1-GFP n=53, CyclinA2-GFP n=58, p27^{Kip1}-GFP n=59, from three independent experiments. **F.** G1 length is significantly longer in p27^{Kip1}-GFP expressing HeLa cells than in CyclinA2-GFP expressing cells (one-way ANOVA, $p<0.05$). However, all p27^{Kip1}-GFP expressing cells progress through G1 successfully and the length of G1 is within the normal range. There is no significant difference in G1 length between CyclinE1-GFP and CyclinA2-GFP, or between CyclinE1-GFP and p27^{Kip1}-GFP cells. Mean +/- STD is shown. Number of cells analysed: CyclinE1-GFP n=52, CyclinA2-GFP n=61, p27^{Kip1}-GFP n=50, from three independent experiments. **G.** Western blot showing degradation of p27^{Kip1}-GFP and endogenous p27^{Kip1} after release from a nocodazole (NZ) block. B-actin was used as a loading control. The lower bands (marked by double asterisk) are the endogenous protein and the upper bands (marked by a single asterisk) are the GFP-tagged protein expressed from the BAC. Graph on the right shows quantification of protein levels over time. For quantification, p27^{Kip1} levels were first normalised to actin and then standardised to an initial p27^{Kip1} level of 1 at 4 hr post NZ release such that the relative rates of degradation could be compared. **H.** Western blot showing degradation of CyclinE1-GFP and endogenous CyclinE1 after release from a double thymidine (Thy) block. B-actin was used as a loading control. The lower bands (marked by double asterisk) are the endogenous protein and the upper bands (marked by a single asterisk) are the GFP-tagged protein expressed from the BAC. Graph on the right shows quantification of protein levels over time. For quantification, CyclinE1 levels were first normalised to actin and then standardised to an initial CyclinE1 level of 1 at 0 hr post Thy release such that the relative rates of degradation could be compared.

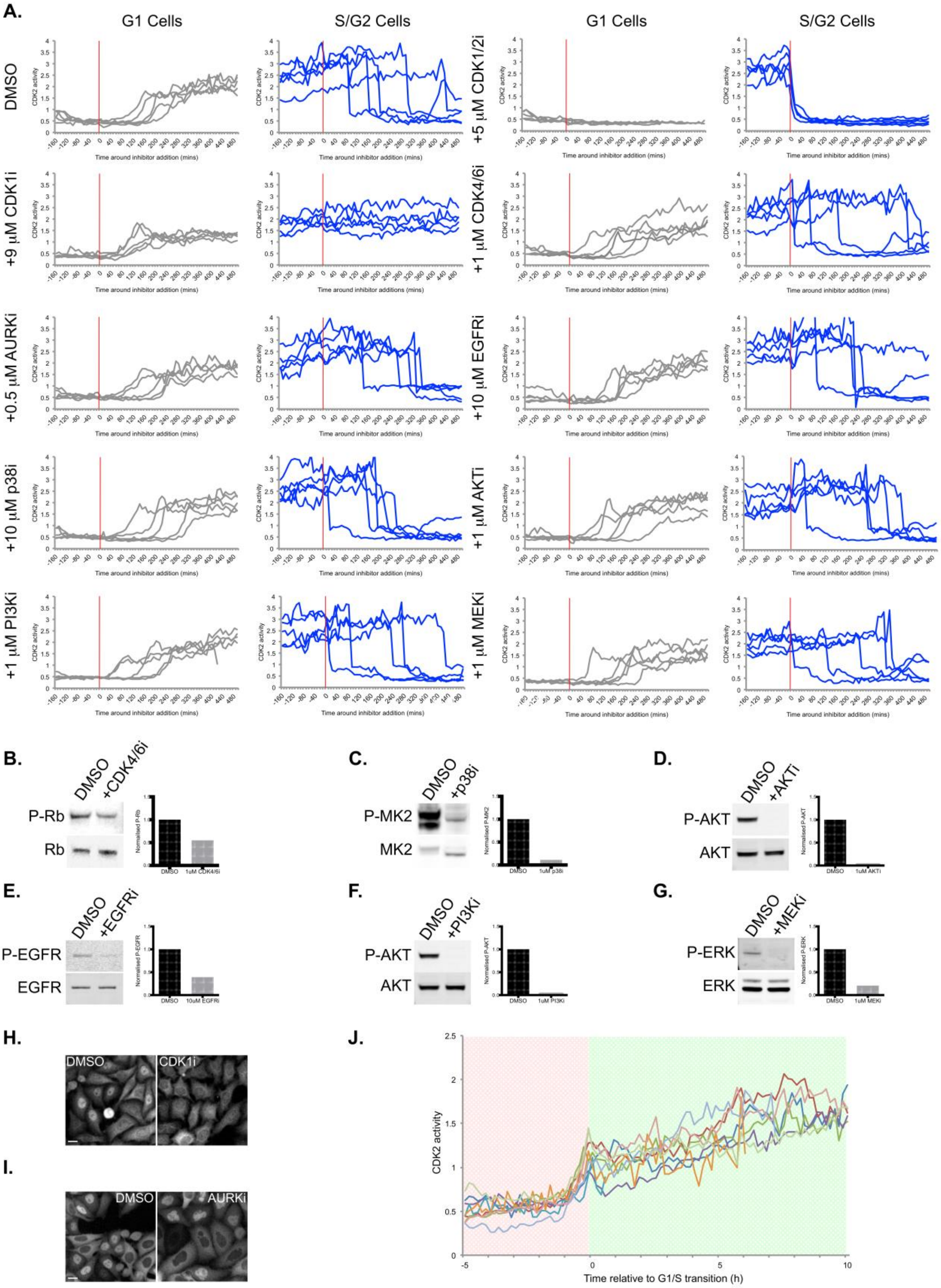

A.
G1 Cells
S/G2 Cells
G1 Cells
S/G2 Cells
DMSO
+5 µM CDK1/2i
+9 µM CDK1i
+1 µM CDK4/6i
+0.5 µM AURKi
+10 µM EGFRi
+10 µM p38i
+1 µM AKTi
+1 µM PI3Ki
+1 µM MEKi
CDK2 activity
Time around inhibitor addition (mins)
B.
DMSO
+CDK4/6i
P-Rb
Rb
C.
DMSO
+p38i
P-MK2
MK2
D.
DMSO
+AKTi
P-AKT
AKT
E.
DMSO
+EGFRi
P-EGFR
EGFR
F.
DMSO
+PI3Ki
P-AKT
AKT
G.
DMSO
+MEKi
P-ERK
ERK
H.
DMSO
CDK1i
I.
DMSO
AURKi
J.
CDK2 activity
Time relative to G1/S transition (h)

Figure S2: Validation of Cdk2 activity sensor, CDK2L-GFP (related to Figure 1). A. Graphs show quantification of Cdk2 activity in individual cells around the time of inhibitor addition. For each drug used, two graphs are shown: on the left (grey curves) are cells that were in G1 at the time of drug addition. On the right (blue curves) are cells that were in S or G2 at the time of drug addition. Red vertical line indicates time of drug addition (time 0 mins). Reporter activity only decreases on addition of the CDK1/2 inhibitor. Rapid decreases in Cdk2 activity in other blue curves are cells that are entering mitosis. Note that after CDK1i addition, cells arrest in G2 (blue curves plateau) indicating that the CDK1i is working effectively. Inhibitors used here are: CDK1/2i - CDK1/2 inhibitor III 217714; CDK1i - RO-3306; CDK4/6i - PD-0332991 (Palbociclib); AURKi - CCT241736; EGFRi - Lapatinib; p38i - SB203580; AKTi - MK2206; PI3Ki - GDC-0941; and MEKi - PD-0325901. Each drug was tested in two independent experiments and the results of one experiment are shown here. **B.** Western blot showing reduction in P-S780 pRb after addition of CDK4/6i. Graph shows quantification of Western blot. Note that this Western blot was performed in MCF10A cells to show that the inhibitor is active since Palbociclib has no effect on pRb phosphorylation in HeLa cells. **C.** Western blot showing reduction in P-T334 MAPKAPK2 (MK2) after addition of p38i. Graph shows quantification of Western blot. **D.** Western blot showing reduction in P-S473 AKT after addition of AKTi. Graph shows quantification of Western blot. **E.** Western blot showing reduction in P-Y1068 EGFR after addition of EGFRi. Graph shows quantification of Western blot. **F.** Western blot showing reduction in P-S473 AKT after addition of Pi3Ki. Graph shows quantification of Western blot. **G.** Western blot showing reduction in P-T202/Y204 ERK after addition of ERKi. Graph shows quantification of Western blot. **H.** Images showing arrest in G2 of CDK1i treated cells. Note, in the right hand image that CDK2L-GFP is in the cytoplasm indicating G2 arrest. Scale bar is 10 μm. **I.** Images showing inhibition of cytokinesis in AURKi treated CDK2L-GFP expressing cells. Image panel on right shows irregular shaped nuclei after 24 hr in AURKi. Scale bar is 10 μm. **J.** Graphs shows quantification of individual cells co-expressing the DHB-Ven Cdk2 activity reporter (Spencer et al., 2013) and fluorescent PCNA to mark S-phase entry. Single cell traces are aligned to S-phase entry.

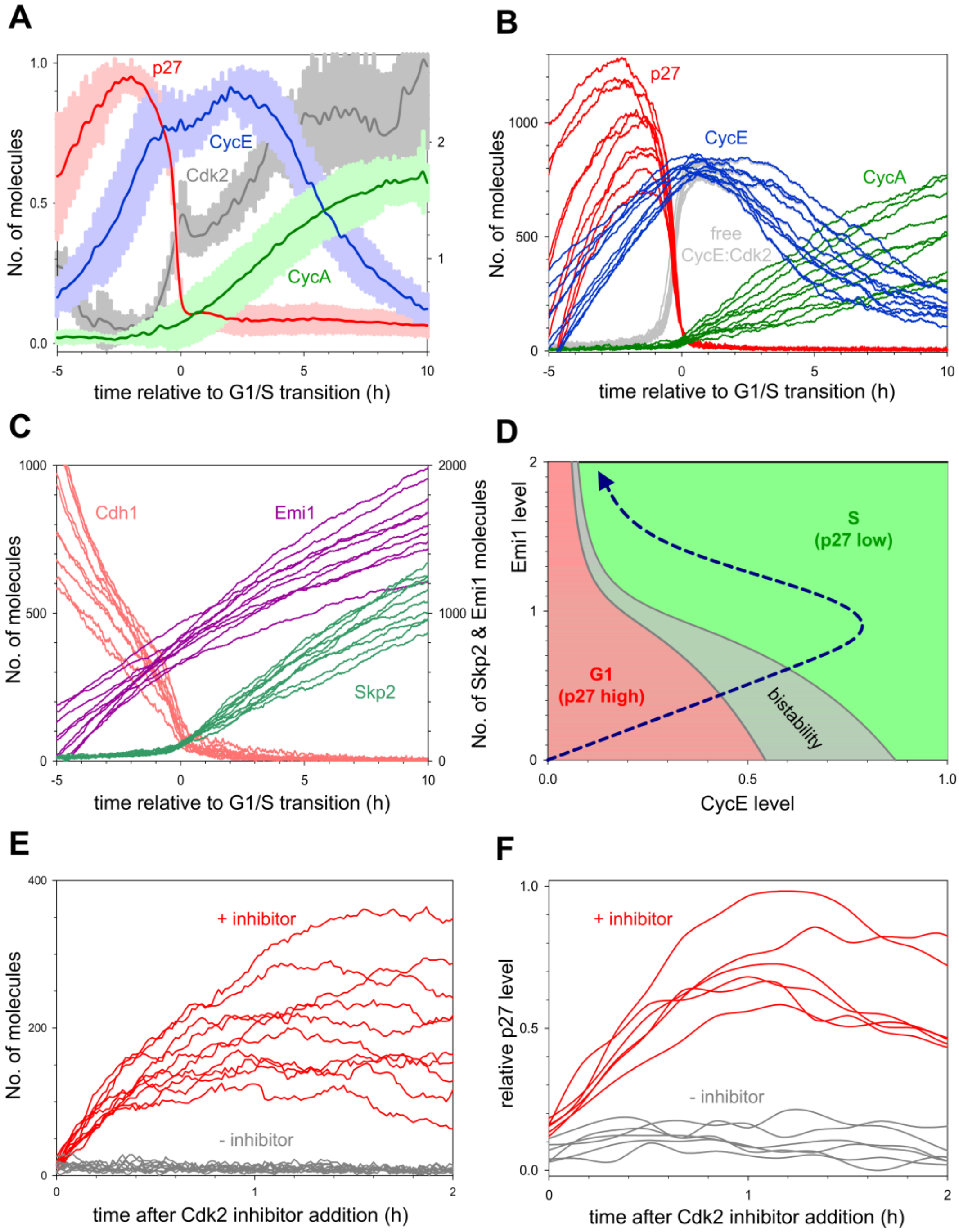

Figure S3: Dynamics of G1/S regulators in unperturbed cells and upon Cdk2 inhibition (related to Figure 2). A. Standard deviation of G1/S aligned and averaged time-courses of experimental measurements of cell cycle reporters and Cdk2 activity, from Fig. 2B. **B.** G1/S aligned stochastic simulations for $p27^{Kip1}$, CyclinE and CyclinA. The curves were aligned to time zero when the number of $p27^{Kip1}$ molecules dropped below 100 (10% of its average peak value). **C.** G1/S aligned stochastic simulations for Emi1, Skp2 and for the active form of Cdh1. **D.** The CyclinE thresholds for $p27^{Kip1}$ inactivation and reactivation from Fig. 2D are plotted as a function of Emi1 levels with the deterministic model. The diagram is divided into three territories: low (green) and high (red) levels of $p27^{Kip1}$ and bistability (grey) where both of these states coexist. The trajectory of cell cycle progression of unperturbed cells is shown by a blue dashed curve. **E**. Stochastic simulations of $p27^{Kip1}$ re-accumulation after Cdk2 inhibition in S-phase arrested cells. Both CyclinE and CyclinA dependent-Cdk2 activities were reduced to 5% at time zero (corresponding to early S-phase). **F**. Quantification of $p27^{Kip1}$-GFP levels after addition of CDK1/2 inhibitor to S-phase arrested cells. $p27^{Kip1}$-GFP accumulates in HeLa cells arrested in S-phase after inhibition of Cdk2 activity. Four independent measurements taken, 6 cells for each treatment from one experiment are shown.

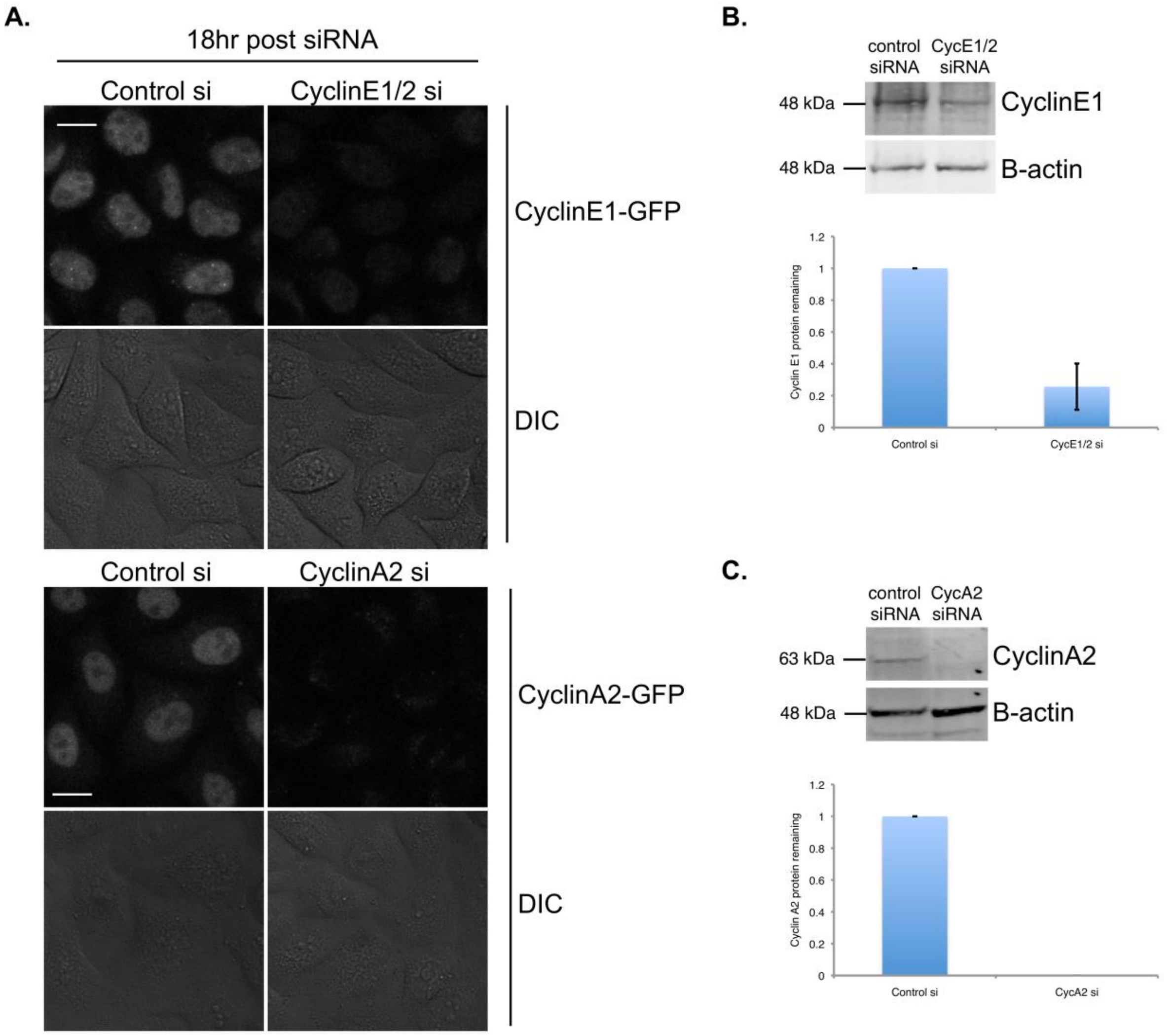

Figure S4: siRNA-mediated depletion of CyclinE1/2 and CyclinA2 (related to Figures 3 and 4). A. Images of CyclinE1-GFP expressing cells treated with CyclinE1/2 siRNA or control siRNA (upper panels) and CyclinA2-GFP expressing cells treated with CyclinA2 siRNA or control siRNA (lower panels) to validate protein knockdown within a single cell cycle. Scale bars are 10 μm. **B.** Representative Western blot for CyclinE1 knockdown. B-actin is used as a loading control. Cells were lysed 18 hr post siRNA transfection (see Supplemental Experimental Procedures). Graph shows quantification of CyclinE1 levels from Western blots, normalised to B-actin. Mean +/- STD is shown (n=4). **C.** Representative Western blot for CyclinA2 knockdown. B-actin is used as a loading control. Cells were lysed 18 hr post siRNA transfection. Graph shows quantification of CyclinA2 levels from Western blots, normalised to B-actin. Mean +/- STD is shown (n=3).

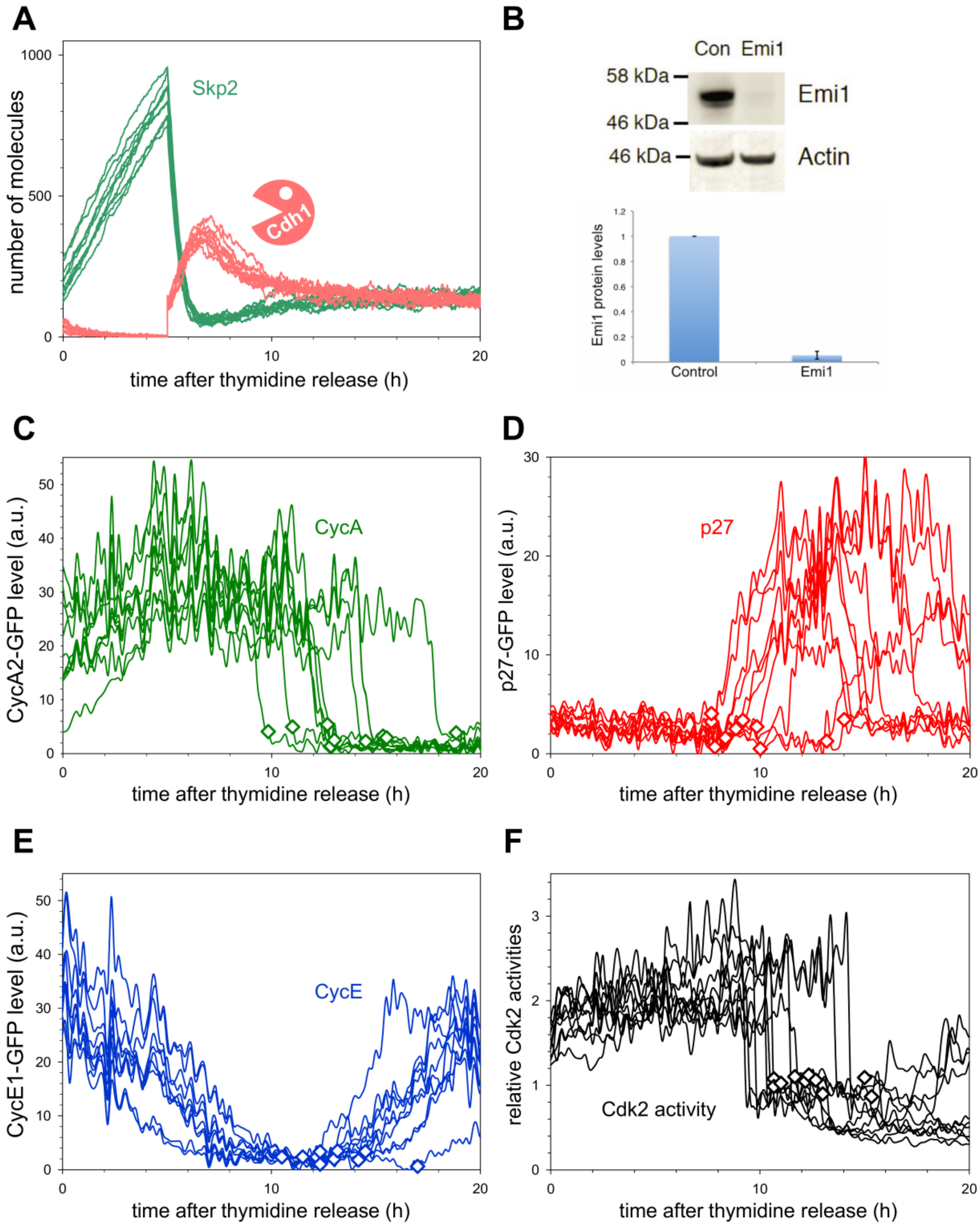

Figure S5: siRNA-mediated depletion of Emi1 (related to Figure 5). A. Stochastic simulations of Emi1 depletion in cells released from a thymidine S-phase block (as in Fig. 5A). Loss of Emi1 (at 5 hr) causes an abrupt reactivation of APC/C^{Cdh1} that promotes degradation of Skp2. **B.** Western blot showing depletion of Emi1 40 hr after the second thymidine release. Graph at the bottom shows quantification of Emi1 depletion. Mean +/- STD is shown (n=2). **C-F.** Quantification of protein/activity levels in cells treated with control siRNA, measured from the time of release from the second thymidine block (corresponds to Fig. 5C-F). Each curve represents a single cell and diamonds represent time of cytokinesis. Note that all control siRNA cells go through mitosis during the filming period. In each graph, 10 cells are shown. At least two independent experiments were conducted in each cell line. **C.** CyclinA2-GFP levels. **D.** p27-GFP levels. **E.** CyclinE1-GFP levels. **F.** Cdk2 activity as measured by the CDK2L-GFP sensor.

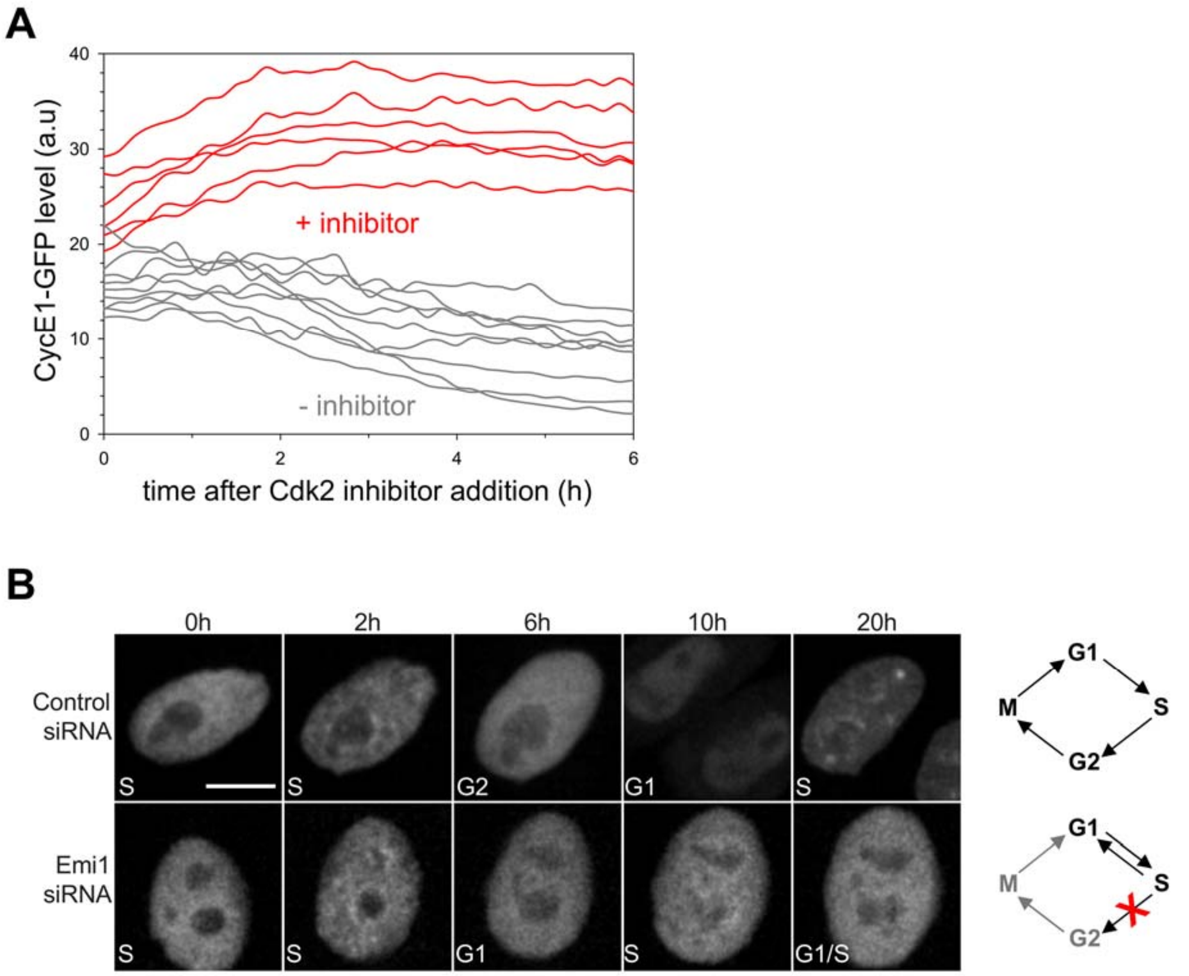

Figure S6: Inhibition of Cdk2 and siRNA-mediated depletion of Emi1 (related to Figure 5). A. Quantification of CyclinE1-GFP protein after addition of CDK1/2 inhibitor to cells in early S-phase (as determined by PCNA foci). CyclinE1-GFP accumulates and is then stabilised after inhibitor addition. Two independent measurements taken, 9 (control) and 6 (inhibitor-treated) cells from one experiment are shown. **B.** Still images taken from Movies S5 and S6 showing expression of fluorescent PCNA in cells treated with Control siRNA (upper panels) or Emi1 siRNA (lower panels). Scale bars are 10 μm. Time 0 hr is release from thymidine block therefore PCNA levels are already high in these cells since they had been arrested in early S-phase. On each panel is the cell cycle stage. The 20h panel for Emi1 siRNA treated cells is labelled as 'G1/S' since cells appear to enter an intermediate state capable of licensing and firing replication origins. On the right are shown the paths through the cell cycle that the cells shown in the images take.

Supplemental Experimental Procedures

Cell culture

HeLa cells (ATCC) were maintained in DMEM (Invitrogen) + 10% FBS (Sigma) and 1% Penicillin/Streptomycin (Invitrogen) at 37°C and 5% CO_2. For live cell imaging, DMEM without phenol red was used.

Generation of reporter cell lines

Tagged BAC constructs were purified using the Nucleobond AX100 kit (Machery-Nagel), according to the manufacturer's protocols for "low-copy plasmid purification". BAC DNA was transfected into HeLa cells using Lipofectamine 2000 (Invitrogen), according to the manufacturer's instructions. The day after transfection, stable clones were selected with 0.4 mg/ml G418 for 7-10 days. After this time, GFP positive cells were sorted to single-cell density in 96-well plates using a FACS Aria (BD Biosciences). Single cell clones were expanded for 7-10 days in selection media and then checked by Western blotting to determine that the full-length protein was tagged. In all cases, the clones with the lowest expression of the GFP-tagged protein were selected. Live-cell imaging was then used to determine that the localisation and dynamics of the tagged protein was the same as described for the endogenous protein.

To monitor PCNA dynamics, we cloned full-length human PCNA cDNA (generous gift of Cristina Cardoso, Technische Universitat Darmstadt) into a modified pIRES-Puro3 vector, where the Puromycin resistance gene had been swapped with a Blasticidin resistance gene. PCNA was tagged at the N-terminus with the modified LSS2-mKate fluorophore to generate LSS2-mKate-PCNA (Piatkevich et al., 2010) (LSS2-mKate cDNA was a generous gift of Vladislav Verkhusha, Albert Einstein College of Medicine, NY). LSS2-mKate-PCNA was transfected into HeLa cell lines using Lipofectamine 2000 (Invitrogen), according to manufacturer's instructions. Stable cell lines were selected over 7-10 days using 0.4 mg/ml G418 and 2 μg/ml Blasticidin (Invitrogen). Single cell clones were then obtained as above, using FACS to sort single cells into 96-well plates. Live-cell imaging was used to determine that the localisation and dynamics of the tagged protein was the same as described for the endogenous protein.

To generate HeLa cells expressing CDK2L-GFP, purified plasmid DNA was transfected into HeLa cells using Lipofectamine 2000 (Invitrogen) according to manufacturer's instructions. Cells were selected in 1 μg/ml Puromycin for 7-10 days and sorted to single cell density, as above, to generate clonal cell lines.

Live Cell Imaging

For imaging on the Zeiss LSM710 confocal microscope, cells were plated in 35 mm uncoated, glass-bottomed Mattek dishes. For imaging on the PerkinElmer Opera high-throughput confocal microscope, cells were plated on 96-well CellCarrier plates (PerkinElmer). Images were captured every 5-10 min and all imaging data was exported as .lsm files (Zeiss) or .flex files (Opera) and imported into Volocity software for analysis and quantification (PerkinElmer).

Degradation time courses

$p27^{Kip1}$-GFP/LSS2-mKate-PCNA cells were arrested in prometaphase by treatment with 40 ng/ml nocodazole for 16 hr. Mitotic shake off was used to collect mitotic cells. Cells were released from this arrest by washing in warm media three times before replating in warm media. Cells were then lysed directly in 1X Laemmli buffer at each timepoint.

CyclinE1-GFP/LSS2-mKate-PCNA cells were arrested in early S-phase by double thymidine synchronisation. After incubation in the second round of thymidine, cells were washed three times in warm PBS and released into warm medium. Cells were then lysed directly in 1X Laemmli buffer at each time-point.

To quantify protein degradation of GFP-tagged and endogenous proteins, each signal was normalised to B-actin at the same time-point. Each normalised value was then scaled to a starting point of 1, such that the degradation of the two proteins could be compared directly on the graph.

Western blotting

Samples for Western blotting were lysed directly in boiling 1X Laemmli buffer. Lysates were separated on 4-20% Precise pre-cast protein gels (Pierce) and transferred to PVDF-FL (Millipore). PVDF membranes were blocked in 5% milk in TBS + 10% glycerol for 1 hr at RT. Membranes were probed with primary antibodies overnight at 4°C. Unbound antibody was removed with three 20 min washes in TBS/0.05% TritonX-100 (TBS/T). Primary antibodies were detected using either anti-mouse DyLight 680 or anti-rabbit DyLight 800 (Cell Signalling) diluted to 1 μg/ml in ROTI-block. Membranes were incubated in the dark for 1 hr at RT in secondary antibodies. Unbound antibody was removed with three 20 min washes in TBS/T. Membranes were

finally washed for 5 min at RT in water before visualising bands on the LiCor imaging system. Protein levels were quantified using Odyssey software and normalised to B-actin, as outlined above. For Emi1 protein detection, anti-mouse HRP secondary antibody was used and proteins were visualised by ECL detection on the Azure digital imaging system.

Primary antibodies used in this study are: CyclinA2 E23.1 (AbCam), CyclinE1 HE12 (AbCam), $p27^{Kip1}$ (BD Bioscience), Emi1 (Zymed), P-S780 pRb (Rb MAb CST C84F6) and Total pRb (Rb MAb CST D20), P-S473 AKT and pan-AKT (CST 9271 and 2920), P-Y1068 EGFR and pan-EGFR (CST 3777 and 2239), P-T202/Y204 ERK and ERK (CST 9106 and 4695), P-T334 MAPKAPK-2 and MAPKAPK-2 (CST 3007 and 12155).

siRNA transfection

All siRNAs were purchased from Dharmacon. Prior to siRNA transfection, cells were washed twice in 1XPBS and incubated in OPTIMEM (Invitrogen). siRNA was transfected into HeLa cells using Lipofectamine RNAiMAX (Invitrogen), according to manufacturer's instructions. siRNA sequences used in this study were: Control siRNA - ON-TARGETplus Non-targeting siRNA #2; CyclinA2 - siGenomePool siRNA, and CyclinE1/E2 – siGenomePool siRNA; Emi1 – custom siRNA Emi1_1 (Di Fiore and Pines, 2007). siRNAs were used at a final concentration of 20 nM.

For synchronisation experiments with CyclinE1/2 and CyclinA2 siRNA, cells were subjected to a double thymidine synchronisation protocol. Briefly, cells were arrested at G1/S with 2 mM Thymidine for 20 hr, released into fresh medium for 9 hr, treated with 2 mM Thymidine a second time for 16 hr, released for 5 hr before cells were transfected with siRNA. After 4 hr incubation in siRNA, cells were imaged to capture cells in the first G1/S transition after protein depletion. We validated that protein was depleted by this time using Western blotting and through visualisation of CyclinE1-GFP or CyclinA2-GFP levels in live cells (Fig. S4A).

For synchronisation experiments with Emi1 siRNA, cells were subjected to a modified double thymidine synchronisation protocol. Cells were arrested at G1/S with 2 mM Thymidine for 20 hr, released into fresh medium for 5 hr then transfected with siRNA. After 4 hr incubation in siRNA cells were again treated with 2 mM Thymidine a second time for 16 hr. Cells were then released form thymidine and filmed immediately or cells were processed for FACS (Fig. 5B) or Western blotting (40 hr after release, Fig. S5B).

Inhibitors

Inhibitors used in this study are: CDK1/2 inhibitor (EMD Biosciences #217714), CDK1 inhibitor RO-3306 (Sigma), CDK4/6 inhibitor (Palbociclib; SelleckChem), Aurora Kinase inhibitor CCT241736 (Bavetsias et al., 2012), EGFR inhibitor (Lapatinib ditosylate, Santa Cruz), AKT inhibitor MK-2206 (SelleckChem), PI3K inhibitor GDC-0941 (SelleckChem), MEK inhibitor PD-0325901 (Sigma), and p38 inhibitor SB203580 (CST). For experiments shown in Fig. S2, a 2X dilution was made of each drug and then either added to cells that were imaged to monitor CDK2 activity or added to cells in a 6-well plate to monitor pathway inhibition by Western blot. Inhibitor was added for 1 hr before cell lysis in all cases. To monitor p38 inhibitor action, we stimulated the cells with sodium arsenite (1/100, Sigma) for the final 10 mins before lysis.

Flow Cytometry

Cells were trypsinised and washed once in cold PBS. Cells were fixed in ice-cold 70% EtOH in PBS at -20 °C overnight. The next day, cells were washed in ice-cold PBS and resuspended in PBS/0.1% Triton X-100 + 20 μg/ml propidium-iodide (Sigma) and 200 μg/ml DNAse-free RNAse A. Cells were stained at RT for 30 min before being stored on ice and analysed on a LSRII (BD Biosciences). FACS data was plotted on FlowJo software.

Mathematical modelling of the G1/S transition

Fig. 2A is our working hypothesis for how Cyclin-dependent kinases and their regulators (e.g. $p27^{Kip1}$, Cdh1) interact to control events of the G1/S transition. The proposed mechanism is based on many reasonable deductions and assumptions drawn from the experimental literature. In order to understand the behaviours of such a complex regulatory network, and to make testable predictions of its emergent properties, we have built a mathematical model of the control system, based on well-established principles of biochemical kinetics, and then used computation to derive precisely the time dependent dynamics of the control network. To this end, the molecular interaction network on Fig. 2A has been converted into a set of ordinary (nonlinear) differential equations (ODE) based on the principle of law of mass action and the following assumptions.

The synthesis and degradation of E2F-independent (p27 and Skp2) and E2F-dependent (CycE, CycA and Emi1) mRNAs and the corresponding proteins are described by the following ODEs:

**mRNA**	**protein**
$\frac{d[p27]_m}{dt} = k^m_{s27} - k^m_{d27} \cdot [p27]_m$	$\frac{d[p27]_T}{dt} = k_{s27} \cdot [p27]_m - V_{dp27} \cdot [p27]_T$
$\frac{d[Skp2]_m}{dt} = k^m_{sskp2} - k^m_{dskp2} \cdot [Skp2]_m$	$\frac{d[Skp2]}{dt} = k_{sskp2} \cdot [Skp2]_m - V_{dskp2} \cdot [Skp2]$
$\frac{d[CycE]_m}{dt} = k^m_{scyce} \cdot [E2F] - k^m_{dcyce} \cdot [CycE]_m$	$\frac{d[CycE]_T}{dt} = k_{scyce} \cdot [CycE]_m - V_{dcyce} \cdot [CycE]_T$
$\frac{d[CycA]_m}{dt} = k^m_{scyca} \cdot [E2F] - k^m_{dcyca} \cdot [CycA]_m$	$\frac{d[CycA]_T}{dt} = k_{scyca} \cdot [CycA]_m - V_{dcyca} \cdot [CycA]_T$
$\frac{d[Emi1]_m}{dt} = k^m_{semi1} \cdot [E2F] - k^m_{demi1} \cdot [Emi1]_m$	$\frac{d[Emi1]_T}{dt} = k_{semi1} \cdot [Emi1]_m - k_{demi1} \cdot [Emi1]_T$

The total levels of proteins existing in more than one form (p27, CycE, CycA and Emi1) are labelled by subscript 'T'. By assuming that the Cyclins associate rapidly with Cdk2 present in excess (Arooz et al., 2000), the total levels of CycE ($CycE_T$) and CycA ($CycA_T$) correspond to the sum of CycE:Cdk2 and CycA:Cdk2 dimers and their trimeric complexes with p27, respectively. We assume that CyclinE accumulates in inactive trimers with Cdk2 and $p27^{Kip1}$ until there is sufficient CyclinE to overcome $p27^{Kip1}$ –mediated inhibition. This assumption is consistent with *in vitro* data describing the binding kinetics of Cdk2:CyclinE and $p27^{Kip1}$, where the transition from a loosely-bound $p27^{Kip1}$ state to a tightly-bound one is fast (1 min^{-1}) compared to a slow off rate (120 min^{-1}; (Sheaff et al., 1997)). This suggests that the vast majority of Cdk2:CyclinE exists in an inhibited trimeric complex during G1 (Swanson et al., 2000), which is consistent with our measurements of Cdk2 activity in HeLa cells.

The first and second order rate constants are indicated by 'k' and both of them have a dimension of $time^{-1}$ because the concentration of molecules is expressed on a relative scale in the deterministic model. Degradation of most proteins is regulated, which is described by specific rate functions ('V'). All rate functions contain a small, first-order rate constant corresponding to slow constitutive degradation in front of regulated degradation terms. The degradation of p27 is promoted by CycE- and CycA-dependent Cdk2 activities and Skp2:

$$V_{dp27} = k_{d27} \; + \; (k_{d27e} \cdot [CycE] + \; k_{d27a} \cdot [CycA]) \cdot [Skp2\,]$$

Auto-phosphorylated CycE becomes a substrate of ubiquitin-dependent proteasomal degradation (Won and Reed, 1996). We assume that CycA-kinase also phosphorylates CycE and thereby promotes its degradation (Kalaszczynska et al., 2009):

$$V_{dcyce} \; = \; k_{dcyce} + \; k_{dcycee} \cdot [CycE] + \; k_{dcycea} \cdot [CycA]$$

The degradation of both CycA and Skp2 are APC/C^{Cdh1}-dependent during G1 phase (Bashir et al., 2004; Wei et al., 2004):

$$V_{dcyca} = \; k_{dcyca} \; + \; k_{dcycac1} \cdot [Cdh1]$$

$$V_{dskp2} = k_{dskp2} \; + \; k_{dskp2c1} \cdot [Cdh1]$$

The p27 stoichiometric Cdk-inhibitor binds reversibly to both CycE:Cdk2 (*CycE*) and CycA:Cdk2 (*CycA*), thereby creating inactive kinase trimers (CycEp27 and CycAp27):

$$\frac{d[CycEp27]}{dt} \; = \; k_{asse} \cdot ([CycE]_T - [CycEp27]) \cdot ([p27]_T - [CycAp27] - [CycEp27]) - (k_{dise} + V_{dp27} + V_{dcyce}) \cdot [CycEp27]$$

$$\frac{d[CycAp27]}{dt} \; = \; k_{assa} \cdot ([CycA]_T - [CycAp27]) \cdot ([p27]_T - [CycAp27] - [CycEp27]) - (k_{disa} + V_{dp27} + V_{dcyca}) \cdot [CycAp27]$$

APC/C^{Cdh1} (labelled as Cdh1) is inactivated by reversible binding of Emi1 (Hsu et al., 2002):

$$\frac{d[EmiC]}{dt} \; = \; k_{asec} \cdot ([Cdh1]_T \; - [\,EmiC]) \cdot ([Emi1]_T \; - \; [EmiC]) \; - \; (k_{diec} \; + \; k_{demi1}) \cdot [EmiC]$$

and by Cdk-dependent phosphorylation (Lukas et al., 1999):

$$\frac{d[Cdh1]_{dp}}{dt} \; = \; k_{acdh1} \cdot ([Cdh1]_T \; - \; [Cdh1]_{dp}) \; - \; V_{icdh1} \cdot [Cdh1]_{dp}$$

where $Cdh1_{dp}$ labels the dephosphorylated forms and

$$V_{icdh1} = k_{icdh1e} \cdot [CycE] + k_{icdh1a} \cdot [CycA]$$

is the rate-function of Cdh1 inactivation by CycE:Cdk2 and CycA:Cdk2.

The only active form of Cdh1 is the dephosphorylated one that is not bound to Emi1:

$$\frac{d[Cdh1]}{dt} = (k_{diec} + k_{demi1}) \cdot \left([Cdh1]_{dp} - [Cdh1]\right) - k_{asec} \cdot [Cdh1] \cdot ([Emi1]_T - [EmiC])$$
$$+ k_{acdh1} \cdot ([Cdh1]_T - [EmiC] - [Cdh1]) - V_{icdh1} \cdot [Cdh1]$$

The levels and the activities of free (not p27 bound) cyclin:Cdk2 complexes are calculated by the following algebraic equations:

$$[CycE] = [CycE]_T - [CycEp27] \quad [CycE]_a = \frac{[CycE]_T - [CycEp27]}{1 + [Inhibitor]}$$

$$[CycA] = [CycA]_T - [CycAp27] \quad [CycA]_a \frac{[CycA]_T - [CycAp27]}{1 + [Inhibitor]}$$

where [Inhibitor] refers to the concentration of the Cdk2 inhibitor relative to its IC_{50} value. It has a value of zero except in the simulations shown in Figure S3E, where its value is 19 which causes a 95% inhibition.

The kinetic parameters of the model (k's) were estimated by numerical simulations of the deterministic model and comparing the calculated trajectories with the pseudo-synchronised average time-courses of cell cycle regulators. The code used with freely available XPPAUT software (http://www.math.pitt.edu/~bard/xpp/xpp.html) is provided below. The values of the kinetic parameters used for simulation of control cells are listed in the XPPAUT code below. For deterministic simulations, the levels of mRNAs were set to one and assumed to be in pseudo-steady state. The initial values of all other dynamic variables were set to zero with the only exceptions of E2F and Cdh1, which were started from a value of one. These initial conditions mimic the state right after cell division, when both Cdh1 and E2F are rapidly reactivated by the APC/C-dependent loss of CycA and CycB (Krek et al., 1994; Sorensen et al., 2001).

The deterministic model has also been used for one- and two-parameter bifurcation diagrams shown on Fig. 2D, Fig. 3B and Fig. S3D.

Stochastic simulations of intrinsic and extrinsic noise were used to capture the non-genetic variability in cell cycle progression of individual HeLa cells. The stochastic fluctuations in the number of mRNA and protein molecules (intrinsic noise) can be accurately simulated by Gillespie's (Gillespie, 2007) Stochastic Simulation Algorithm (SSA). To this end, the rate expressions of individual reactions of the deterministic model were converted into propensity functions. This conversion transforms the relative concentrations (levels) of cell cycle regulators into number of molecules in a given volume. The stochastic fluctuations in the number of mRNA and protein molecules have been followed in a volume of 1000 units using *MatLab* code. Given high copy numbers of cell cycle control proteins, most of the intrinsic noise in cell cycle control has transcriptional origin (Kar et al., 2009). In accordance with sparse experimental data, the probability of transcription and degradation for all mRNAs was set to 0.2 min^{-1} and 0.002 min^{-1}, respectively, which yields about 100 mRNA molecules in steady state (see (Schwanhausser et al., 2011)). Unequal distribution of cell cycle regulators introduces extrinsic noise, which has been simply captured in our stochastic simulations by assuming Gaussian distribution in the number of E2F molecules among individual cells $\sigma_{E2F}^2 = 0.015$.

In case of siRNA-mediated cyclin depletion, the rate constants of the cyclin mRNA degradation were increased 20-fold, which reduced the steady state level of the targeted cyclin's mRNA to 5% of the level in untreated cells.

```
################################################################################
#           A MATHEMATICAL MODEL FOR G1/S TRANSITION IN HeLa CELLS
################################################################################

################################################################################
#    INITITAL CONDITIONS CORRESPONDING TO THE TIME OF CELL DIVISION
initial CycET=0, CycAT=0, p27T=0, CycEp27=0, CycAp27=0
initial Cdh1dp=1, Emi1T=0, EmiC=0, Cdh1=1 Skp2=0

################################################################################
#    DIFFERENTIAL EQUATIONS
CycET' = kscyce - Vdcyce*CycET
CycAT' = kscyca - Vdcyca*CycAT
p27T' = ks27 - Vdp27*p27T
CycEp27' = kasse*(CycET-CycEp27)*(p27T-CycAp27-CycEp27)-(kdise+Vdp27+Vdcyce)*CycEp27
CycAp27' = kassa*(CycAT-CycAp27)*(p27T-CycAp27-CycEp27)-(kdisa+Vdp27+Vdcyca)*CycAp27
Cdh1dp' = kacdh1*(Cdh1T-Cdh1dp) - Vicdh1*Cdh1dp
Emi1T' = ksemi1 - kdemi1*Emi1T
EmiC' = kasec*(Cdh1T - EmiC)*(Emi1T-EmiC) - (kdiec+kdemi1)*EmiC
Cdh1' = kacdh1*(Cdh1T-EmiC-Cdh1)-Vicdh1*Cdh1-kasec*Cdh1*(Emi1T-EmiC) \
          + (kdiec+kdemi1)*(Cdh1dp-Cdh1)
Skp2' = ksskp2 - (kdskp2 + kdskp2c1*Cdh1)*Skp2

################################################################################
#    RATE-FUNCTIONS FOR THE DEGRADATION OF p27, CyCE, CyCA
#    AND FOR INACTIVATION OF CDH1 BY Cdk2:CycA/E
Vdp27 = (kd27e*CycE/(1+Inhibitor) + kd27a*CycA/(1+Inhibitor))*Skp2 + kd27
Vdcyca = kdcyca + kdcycac1*Cdh1
Vdcyce = kdcyce + kdcycee*CycE/(1 + Inhibitor) + kdcycea*CycA/(1 + Inhibitor)
Vicdh1 = kicdh1e*CycE/(1+Inhibitor) + kicdh1a*CycA/(1+Inhibitor)

################################################################################
#     ALGEBRAIC EQUATIONS FOR FREE (NOT p27 BOUND)  Cdk2:CycE/A complexes
CycA = CycAT - CycAp27
CycE = CycET - CycEp27
aux CycA = CycAT - CycAp27
aux CycE = CycET - CycEp27
################################################################################
#         PARAMETER VALUES
##  CYCE SYNTHESIS, DEGRADATION AND P27 BINDING/DISSOCIATION:
par kscyce=0.003, kdcyce=0.001, kdcycee=0.0001, kdcycea=0.03, kasse=1, kdise=0.02
##  CYCA SYNTHESIS, DEGRADATION AND P27 BINDING/DISSOCIATION:
par kscyca=0.0025, kdcyca=0.002, kdcycac1=0.4, kassa=1, kdisa=0.02
##  P27 SYNTHESIS AND DEGRADATION:
par ks27=0.008, kd27=0.004, kd27e=2, kd27a=2
##  EMI1 SYNTHESIS AND DEGRADATION:
par ksemi1=0.003, kdemi1=0.001
##  CDH1 REGULATION:
par Cdh1T=1, kacdh1=0.02, kicdh1e=0.07, kicdh1a=0.2, kasec=2, kdiec=0.02
##  SKP2 SYNTHESIS AND DEGRADATION:
par ksskp2=0.004, kdskp2=0.002, kdskp2c1=0.2
##  CDK INHIBITOR
par Inhibitor=0
################################################################################
#        NUMERICAL SETTINGS
@ method=stiff, total=900, bound=100, Maxstore=100000, bound=2000, dt=1, xlo=0, xhi=900
@ ylo=0, yhi=1, NPLOT=5, yp1=p27T, yp2=CycET, yp3=CycAT, yp4=CycE, yp5=cycA
done
```

Supplemental Movies

Movie S1: p27^{Kip1}-GFP/LSS2-mKate PCNA expressing HeLa cells (related to Figure 1A).
From left to right: p27^{Kip1}-GFP, LSS2-mKate PCNA, Brightfield.

Movie S2: CyclinE1-GFP/LSS2-mKate PCNA expressing HeLa cells (related to Figure 1B).
From left to right: CyclinE1-GFP, LSS2-mKate PCNA, Brightfield.

Movie S3: CyclinA2-GFP/LSS2-mKate PCNA expressing HeLa cells (related to Figure 1C).
From left to right: CyclinA2-GFP, LSS2-mKate PCNA, Brightfield.

Movie S4: CDK2L-GFP/LSS2-mKate PCNA expressing HeLa cells (related to Figure 1D).
From left to right: CDK2L-GFP, LSS2-mKate PCNA, Brightfield.

Movie S5: GFP-PCNA expressing HeLa cells treated with Control siRNA (related to Figure S6B).

Movie S6: GFP-PCNA expressing HeLa cells treated with Emi1 siRNA (related to Figure S6B).

Supplemental References

Arooz, T., Yam, C.H., Siu, W.Y., Lau, A., Li, K.K., and Poon, R.Y. (2000). On the concentrations of cyclins and cyclin-dependent kinases in extracts of cultured human cells. Biochemistry *39*, 9494-9501.

Bashir, T., Dorrello, N.V., Amador, V., Guardavaccaro, D., and Pagano, M. (2004). Control of the SCF(Skp2-Cks1) ubiquitin ligase by the APC/C(Cdh1) ubiquitin ligase. Nature *428*, 190-193.

Bavetsias, V., Crumpler, S., Sun, C., Avery, S., Atrash, B., Faisal, A., Moore, A.S., Kosmopoulou, M., Brown, N., Sheldrake, P.W., *et al.* (2012). Optimization of imidazo[4,5-b]pyridine-based kinase inhibitors: identification of a dual FLT3/Aurora kinase inhibitor as an orally bioavailable preclinical development candidate for the treatment of acute myeloid leukemia. J Med Chem *55*, 8721-8734.

Carrano, A.C., Eytan, E., Hershko, A., and Pagano, M. (1999). SKP2 is required for ubiquitin-mediated degradation of the CDK inhibitor p27. Nat Cell Biol *1*, 193-199.

den Elzen, N., and Pines, J. (2001). Cyclin A is destroyed in prometaphase and can delay chromosome alignment and anaphase. The Journal of cell biology *153*, 121-136.

Di Fiore, B., and Pines, J. (2007). Emi1 is needed to couple DNA replication with mitosis but does not regulate activation of the mitotic APC/C. The Journal of cell biology *177*, 425-437.

Dulic, V., Lees, E., and Reed, S.I. (1992). Association of human cyclin E with a periodic G1-S phase protein kinase. Science *257*, 1958-1961.

Gillespie, D.T. (2007). Stochastic simulation of chemical kinetics. Annu Rev Phys Chem *58*, 35-55.

Hsu, J.Y., Reimann, J.D., Sorensen, C.S., Lukas, J., and Jackson, P.K. (2002). E2F-dependent accumulation of hEmi1 regulates S phase entry by inhibiting APC(Cdh1). Nat Cell Biol *4*, 358-366.

Kalaszczynska, I., Geng, Y., Iino, T., Mizuno, S., Choi, Y., Kondratiuk, I., Silver, D.P., Wolgemuth, D.J., Akashi, K., and Sicinski, P. (2009). Cyclin A is redundant in fibroblasts but essential in hematopoietic and embryonic stem cells. Cell *138*, 352-365.

Kar, S., Baumann, W.T., Paul, M.R., and Tyson, J.J. (2009). Exploring the roles of noise in the eukaryotic cell cycle. Proceedings of the National Academy of Sciences of the United States of America *106*, 6471-6476.

Koff, A., Giordano, A., Desai, D., Yamashita, K., Harper, J.W., Elledge, S., Nishimoto, T., Morgan, D.O., Franza, B.R., and Roberts, J.M. (1992). Formation and activation of a cyclin E-cdk2 complex during the G1 phase of the human cell cycle. Science *257*, 1689-1694.

Krek, W., Ewen, M.E., Shirodkar, S., Arany, Z., Kaelin, W.G., Jr., and Livingston, D.M. (1994). Negative regulation of the growth-promoting transcription factor E2F-1 by a stably bound cyclin A-dependent protein kinase. Cell *78*, 161-172.

Liu, J., Hebert, M.D., Ye, Y., Templeton, D.J., Kung, H., and Matera, A.G. (2000). Cell cycle-dependent localization of the CDK2-cyclin E complex in Cajal (coiled) bodies. Journal of cell science *113 (Pt 9)*, 1543-1552.

Lukas, C., Sorensen, C.S., Kramer, E., Santoni-Rugiu, E., Lindeneg, C., Peters, J.M., Bartek, J., and Lukas, J. (1999). Accumulation of cyclin B1 requires E2F and cyclin-A-dependent rearrangement of the anaphase-promoting complex. Nature *401*, 815-818.

Piatkevich, K.D., Hulit, J., Subach, O.M., Wu, B., Abdulla, A., Segall, J.E., and Verkhusha, V.V. (2010). Monomeric red fluorescent proteins with a large Stokes shift. Proceedings of the National Academy of Sciences of the United States of America *107*, 5369-5374.

Schwanhausser, B., Busse, D., Li, N., Dittmar, G., Schuchhardt, J., Wolf, J., Chen, W., and Selbach, M. (2011). Global quantification of mammalian gene expression control. Nature *473*, 337-342.

Sheaff, R.J., Groudine, M., Gordon, M., Roberts, J.M., and Clurman, B.E. (1997). Cyclin E-CDK2 is a regulator of p27Kip1. Genes Dev *11*, 1464-1478.

Sorensen, C.S., Lukas, C., Kramer, E.R., Peters, J.M., Bartek, J., and Lukas, J. (2001). A conserved cyclin-binding domain determines functional interplay between anaphase-promoting complex-Cdh1 and cyclin A-Cdk2 during cell cycle progression. Molecular and cellular biology *21*, 3692-3703.

Spencer, S.L., Cappell, S.D., Tsai, F.C., Overton, K.W., Wang, C.L., and Meyer, T. (2013). The proliferation-quiescence decision is controlled by a bifurcation in CDK2 activity at mitotic exit. Cell *155*, 369-383.

Swanson, C., Ross, J., and Jackson, P.K. (2000). Nuclear accumulation of cyclin E/Cdk2 triggers a concentration-dependent switch for the destruction of p27Xic1. Proceedings of the National Academy of Sciences of the United States of America *97*, 7796-7801.

Wei, W., Ayad, N.G., Wan, Y., Zhang, G.J., Kirschner, M.W., and Kaelin, W.G., Jr. (2004). Degradation of the SCF component Skp2 in cell-cycle phase G1 by the anaphase-promoting complex. Nature *428*, 194-198.

Won, K.A., and Reed, S.I. (1996). Activation of cyclin E/CDK2 is coupled to site-specific autophosphorylation and ubiquitin-dependent degradation of cyclin E. The EMBO journal *15*, 4182-4193.
