# Supplementary figures and images for: A Dynamical Framework for the All-or-None G1/S Transition
Source: Cell Syst. 2016 Jan 27;2(1):27–37. doi: 10.1016/j.cels.2016.01.001 (PMC4802413; doi:10.1016/j.cels.2016.01.001)

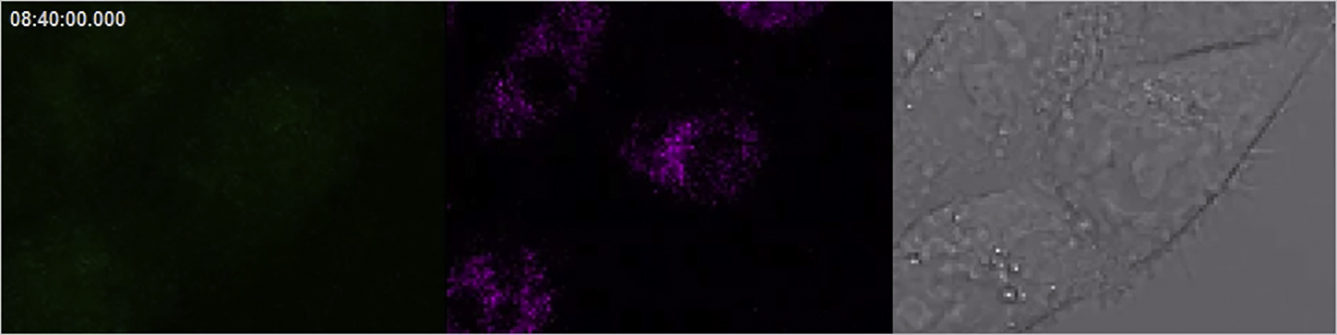

Supplement: Movie S1. p27Kip1-GFP/LSS2-mKate PCNA Expressing HeLa Cells, Related to Figure 1A [file mmc2.jpg]

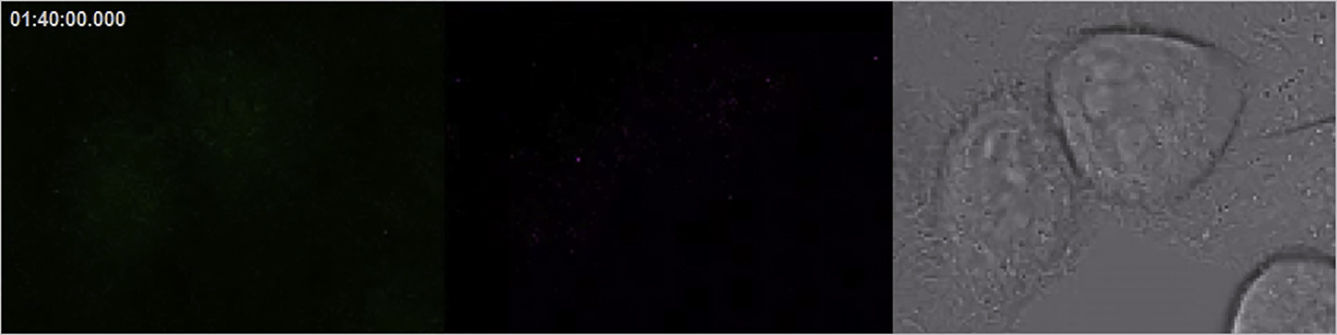

Supplement: Movie S2. CyclinE1-GFP/LSS2-mKate PCNA Expressing HeLa Cells, Related to Figure 1B [file mmc3.jpg]

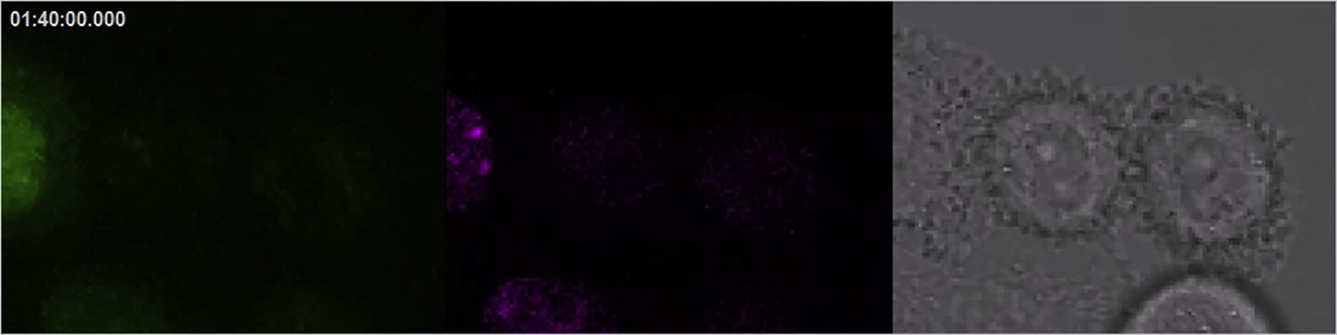

Supplement: Movie S3. CyclinA2-GFP/LSS2-mKate PCNA Expressing HeLa Cells, Related to Figure 1E [file mmc4.jpg]

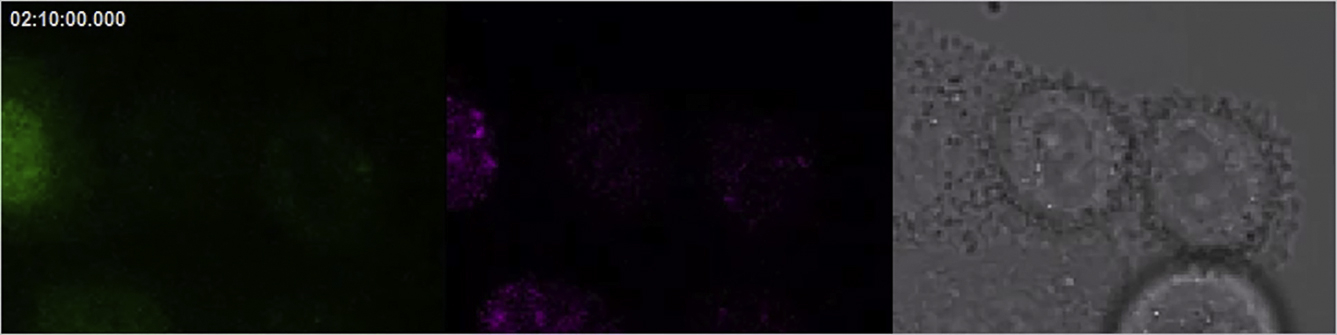

Supplement: Movie S4. CDK2L-GFP/LSS2-mKate PCNA Expressing HeLa Cells, Related to Figure 1D [file mmc5.jpg]

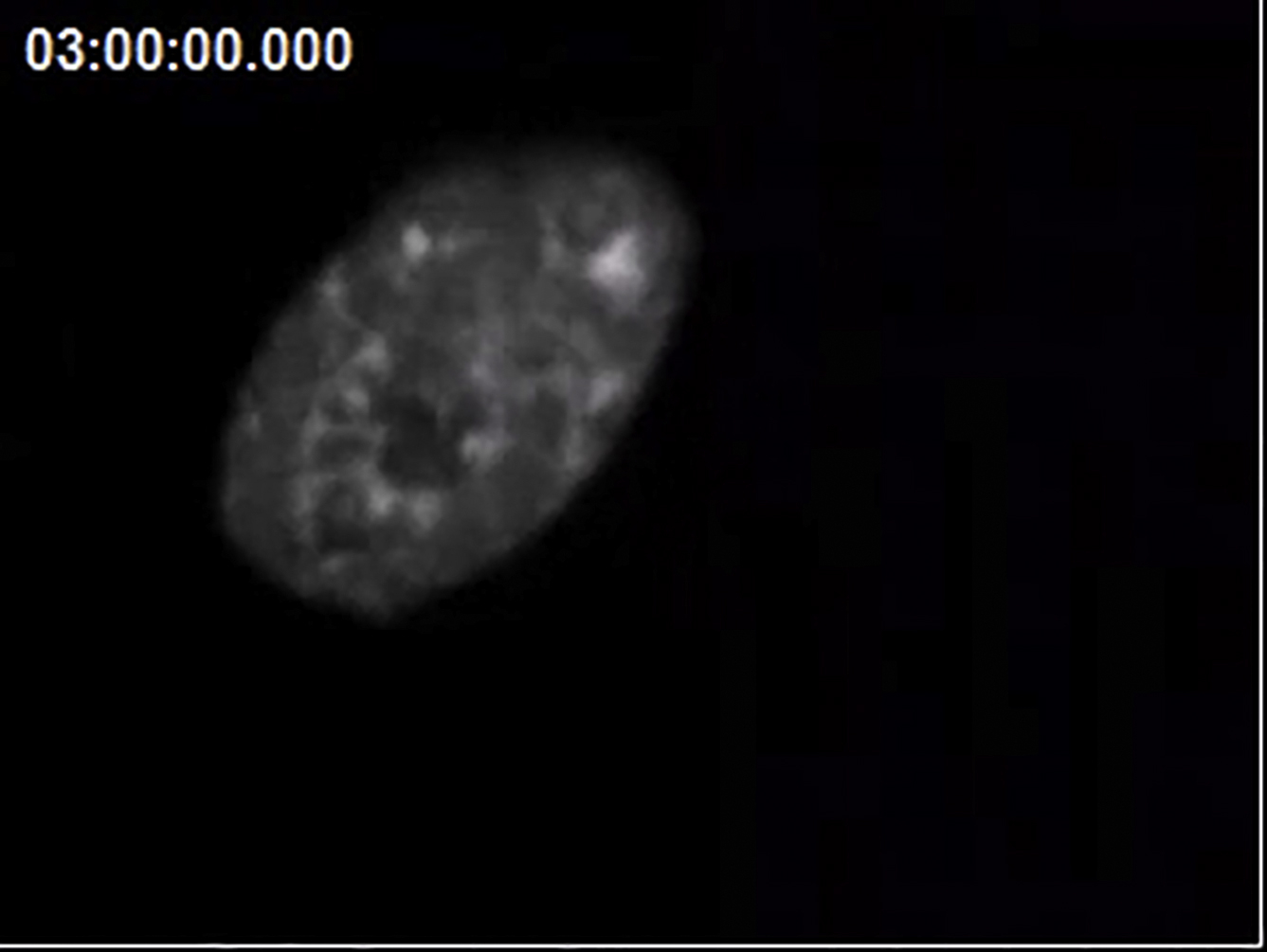

Supplement: Movie S5. GFP-PCNA Expressing HeLa Cells, Related to Figure S6B [file mmc6.jpg]

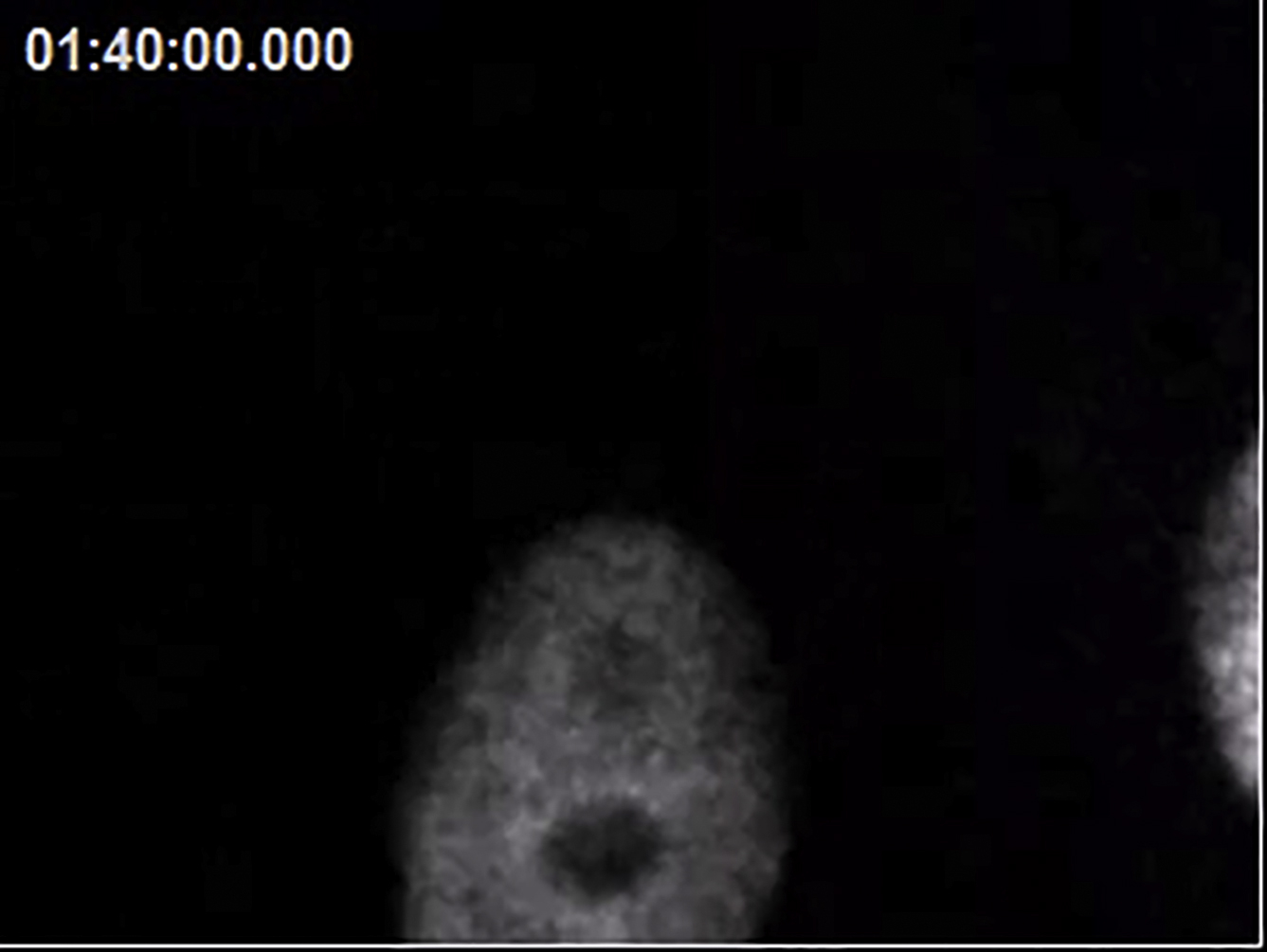

Supplement: Movie S6. GFP-PCNA-Expressing HeLa Cells Treated with Emi1 siRNA, Related to Figure S6B [file mmc7.jpg]
