# Supplementary material for: A Dynamical Framework for the All-or-None G1/S Transition
Source: Cell Syst. 2016 Jan 27;2(1):27–37. doi: 10.1016/j.cels.2016.01.001 (PMC4802413; doi:10.1016/j.cels.2016.01.001)
Supplement: Document S2. Article plus Supplemental Information [file mmc8.pdf]

Cell Systems

Article

A Dynamical Framework for the All-or-None G1/S Transition

Graphical Abstract

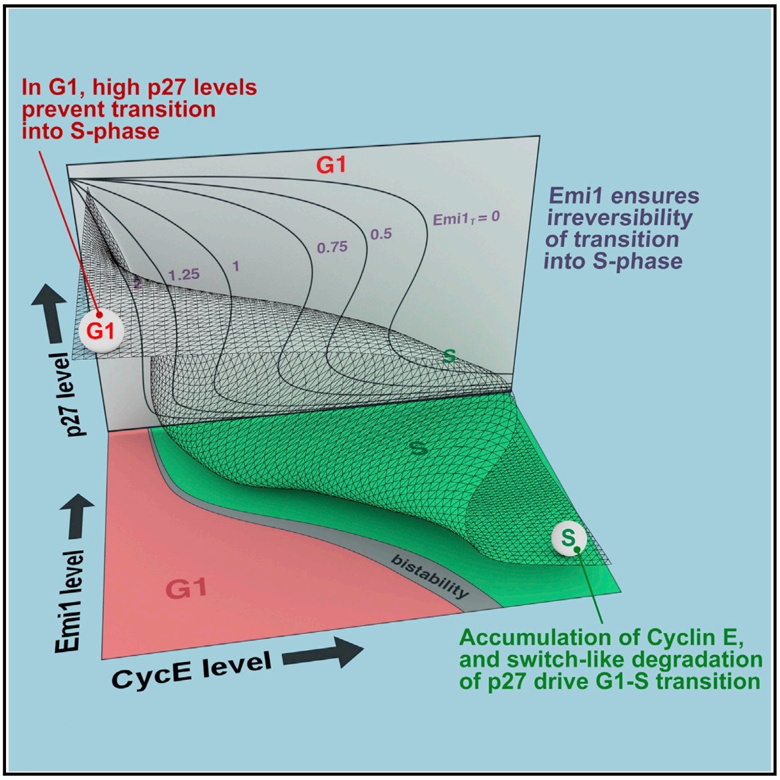

Authors

Alexis R. Barr, Frank S. Heldt, Tongli Zhang, Chris Bakal, Béla Novák

Correspondence

chris.bakal@icr.ac.uk (C.B.), bela.novak@bioch.ox.ac.uk (B.N.)

In Brief

The principles underlying switch-like and irreversible transitions between cell-cycle phases are poorly understood in mammalian cells. Barr et al. have used quantitative real-time imaging of regulators of the G1-to-S transition in single human cells to develop a mechanistic model to show how this network drives rapid and irreversible commitment to cell-cycle progression.

Highlights

- A model of the human G1/S transition is derived from single-cell imaging of key regulators
- Double-negative feedback between Cdk2:CycE and p27 drives a switch-like transition
- The APC/C inhibitor Emi1 ensures that the G1/S transition is kept irreversible
- Modeling provides insight into the differences in Cdk2 activity between cancer and normal cells

Barr et al., 2016, Cell Systems 2, 27–37
January 27, 2016 ©2016 The Authors
http://dx.doi.org/10.1016/j.cels.2016.01.001

OPEN ACCESS
CellPress

A Dynamical Framework for the All-or-None G1/S Transition

Alexis R. Barr,[1] Frank S. Heldt,[2] Tongli Zhang,[2] Chris Bakal,[1,*] and Béla Novák[2,*]
[1]Division of Cancer Biology, Institute of Cancer Research, 237 Fulham Road, London SW3 6JB, UK
[2]Department of Biochemistry, University of Oxford, South Parks Road, Oxford OX1 3QU, UK
*Correspondence: chris.bakal@icr.ac.uk (C.B.), bela.novak@bioch.ox.ac.uk (B.N.)
http://dx.doi.org/10.1016/j.cels.2016.01.001
This is an open access article under the CC BY license (http://creativecommons.org/licenses/by/4.0/).

SUMMARY

The transition from G1 into DNA replication (S phase) is an emergent behavior resulting from dynamic and complex interactions between cyclin-dependent kinases (Cdks), Cdk inhibitors (CKIs), and the anaphase-promoting complex/cyclosome (APC/C). Understanding the cellular decision to commit to S phase requires a quantitative description of these interactions. We apply quantitative imaging of single human cells to track the expression of G1/S regulators and use these data to parametrize a stochastic mathematical model of the G1/S transition. We show that a rapid, proteolytic, double-negative feedback loop between Cdk2:Cyclin and the Cdk inhibitor p27^{Kip1} drives a switch-like entry into S phase. Furthermore, our model predicts that increasing Emi1 levels throughout S phase are critical in maintaining irreversibility of the G1/S transition, which we validate using Emi1 knockdown and live imaging of G1/S reporters. This work provides insight into the general design principles of the signaling networks governing the temporally abrupt transitions between cell-cycle phases.

INTRODUCTION

Through decades of painstaking genetic and biochemical research, the components regulating cell-cycle progression have been identified, but how these components dynamically interact to drive this progression in either normal or cancerous human cells is poorly understood. In mammalian cells, the decision to enter the cell cycle is made at the restriction point, which is analogous to the Start transition in yeast (Johnson and Skotheim, 2013; Zetterberg et al., 1995). Restriction point/Start is followed by a separate G1/S transition where the cell becomes committed to DNA replication. In yeast, Start is characterized by a transcriptional burst that leads to the accumulation of G1- and S-phase cyclins (Clns and Clbs, respectively). This transcriptional activation is driven by a positive feedback loop (Skotheim et al., 2008) that makes Start an irreversible transition (Charvin et al., 2010). The accumulation of S-phase cyclins is necessary, but not sufficient, to initiate DNA replication because the activity of Cdk1:Clb complexes is initially masked by their stoichiometric inhibitor, the Cyclin-dependent Kinase Inhibitor (CKI) Sic1 (Schwob et al., 1994); note that the colon indicates that Cdk1 and Clb are in a protein complex. The G1/S transition is triggered by the proteolytic degradation of Sic1. Sic1 degradation is initiated by Cln:Cdk1 activity (Nash et al., 2001) and continues to be rapidly degraded by the Clb:Cdk1 kinases as they become activated (Kõivomägi et al., 2011; Yang et al., 2013).

A transcriptional positive-feedback loop similar to the Start network in yeasts operates at the restriction point in mammalian cells (Bertoli et al., 2013; Cross et al., 2011). CyclinE:Cdk2 promotes *CyclinE* transcription through activation of its transcription factor (E2F) (Geng et al., 1996; Ohtani et al., 1995; Weinberg, 1995). A bistable switch generated by transcriptional feedback makes the transition through the restriction point irreversible (Yao et al., 2008). Following the transition past the restriction point, E2F-mediated transcription leads to the accumulation of both CyclinE and CyclinA, which form complexes with Cdk2, resulting in its activation, which serves as the trigger for S-phase entry. Similar to yeast, activation of Cdk2:S-phase Cyclin complexes requires the proteolytic degradation of their stoichiometric CKIs, p27^{Kip1}, and p21^{Cip1} (Sherr and Roberts, 1999).

The proteolytic double-negative feedback between CKI and S-phase kinases in yeast leads to G1 and S being two discrete, alternative states. Furthermore, this double-negative feedback loop ensures that commitment to S phase in yeast is irreversible (Chen et al., 2000; Cross et al., 2002; Verdugo et al., 2013; Yang et al., 2013). While the core regulatory machinery (i.e., the proteolytic double-negative feedback loop) of the G1/S transition is conserved between yeast and man, this machinery has been extensively elaborated upon. This elaboration is in the form of many more regulatory components (Cyclins, CDKs, CKIs), with sometimes partially overlapping functions, and additional interactions between conserved components (Cross et al., 2011). Thus, an unresolved question is whether the mammalian G1/S transition exhibits similar system-level properties as yeast. Answering this question is important not only to gain insight into the mammalian G1/S transition, but also to understand how particular behaviors observed in yeast, such as the irreversible switching between discrete states, can be retained when the size and complexity of the regulatory networks governing these behaviors increase during evolution.

Describing the systems-level interactions between Cdk2, CyclinE, CyclinA, and CKI is also highly relevant to gaining insight into the dysregulated G1/S progression of cancer cells, where mutations and epigenetic events often conspire to increase

OPEN
ACCESS
CellPress

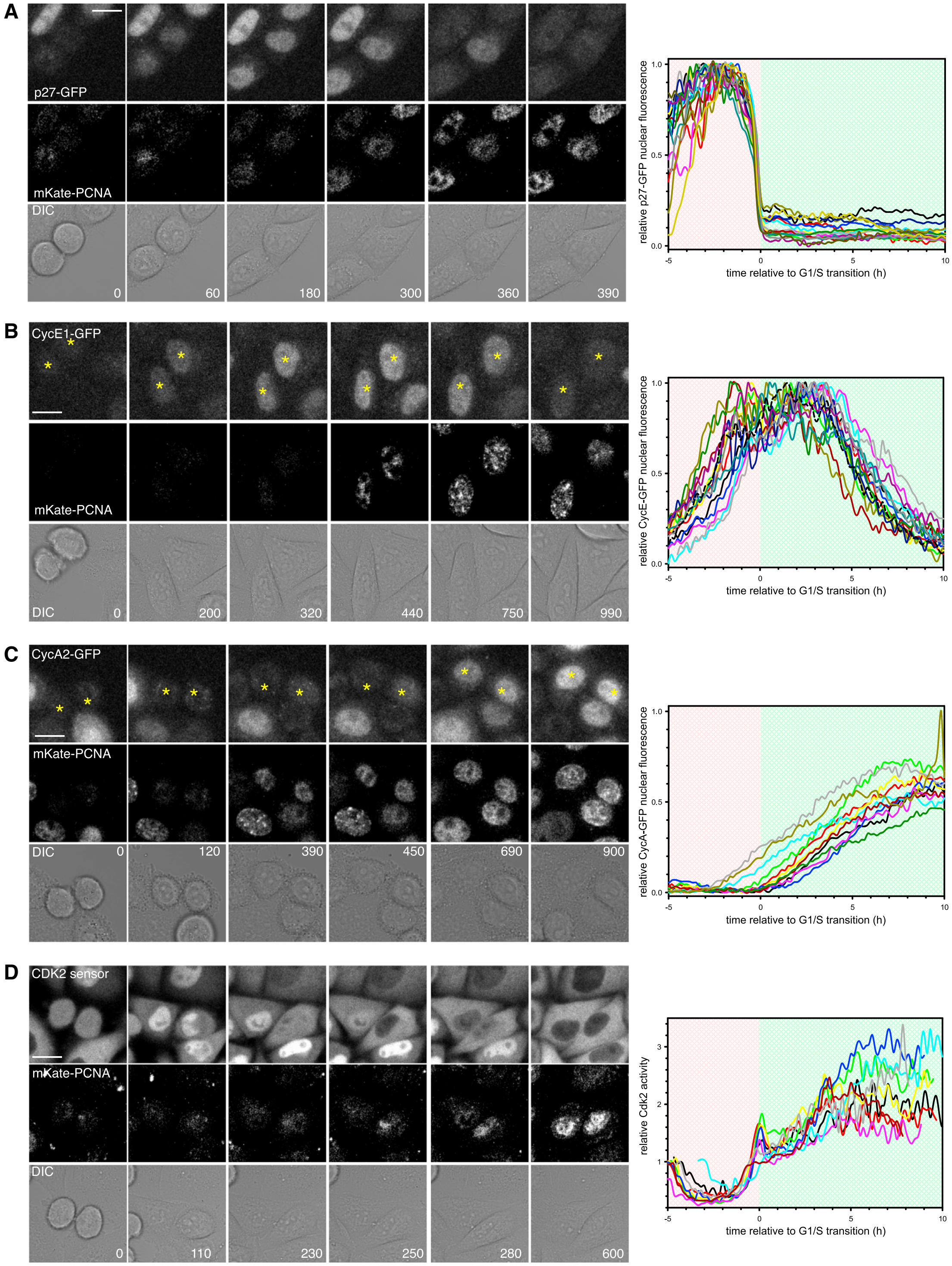

Figure 1. Quantifying the Dynamics of G1/S Regulators in Single Cells

(A) Stills taken from Movie S1 of HeLa cells stably expressing p27^{Kip1}-GFP and LSS2-mKate-PCNA. Graph shows quantification of p27^{Kip1}-GFP from individual cells, aligned to the G1/S transition. Four independent measurements were taken, and 17 cells from one experiment are shown.

(B) Stills taken from Movie S2 of HeLa cells stably expressing CyclinE1-GFP and LSS2-mKate-PCNA. Graph shows quantification of CyclinE1-GFP from individual cells, aligned to the G1/S transition. Three independent measurements were taken, and 15 cells from one experiment are shown.

(C) Stills taken from Movie S3 of HeLa cells stably expressing CyclinA2-GFP and LSS2-mKate-PCNA. Graph shows quantification of Cyclin A2-GFP from individual cells, aligned to the G1/S transition. Four independent measurements were taken, and 11 cells from one experiment are shown.

(legend continued on next page)

CyclinE and downregulate p27^{Kip1} levels (Chu et al., 2008; Fero et al., 1996; Hershko, 2010; Kiyokawa et al., 1996; Nakayama et al., 1996; Scaltriti et al., 2011). Moreover, many cancer cells do not have an intact restriction point (Asghar et al., 2015), and thus, inhibition of the G1/S transition is an attractive therapeutic target to stop these cancer cells from proliferating. To inhibit S-phase entry effectively, we need a quantitative understanding of how Cdk2 activity is a function of Cyclins, CKIs, and APC/C components and how this drives cell-cycle progression.

Only a handful of studies have been able to describe the systems-level logic of progression through different cell-cycle phases in mammalian cells (Pomerening et al., 2008; Yao et al., 2008; Yuan et al., 2014), likely because it has been difficult to quantify the levels and/or activity of different network components at a single cell level. Single cell, time-lapse data are essential to generate predictive quantitative models because such models require information not only on the signaling state of a cell at a given time, but also regarding the cell's history, which is unattainable using population-based measurements.

Here we describe an experimental system to quantify the real-time protein expression of key regulatory molecules involved in the G1/S transition (CyclinA2, CyclinE1, and p27^{Kip1}) and Cdk2 activity. We correlate these changes with cellular fate (initiation of DNA replication) in single proliferating cells. We chose to use HeLa cells as they are an excellent model for studying dysregulated G1/S progression as like almost all cancer cells, they have lost the function of pRb (an inhibitor of E2F), do not have an intact restriction point, and enter G1/S directly after cell division (Moody and Laimins, 2010; Schwarz et al., 1985). We used single cell imaging data of protein levels throughout the G1/S transition to construct a mathematical model of this transition. This work reveals that a switch-like G1/S transition is driven by the mutual antagonism between Cdk2 and p27^{Kip1}. Furthermore, our model provides a mechanistic explanation for how Emi1 keeps the G1/S transition irreversible to prevent DNA re-replication.

RESULTS

Establishing a System to Analyze the Dynamics of the G1/S Transition in Human Cancer Cells

We first sought to follow the dynamics of Cyclin and p27^{Kip1} expression in HeLa cells at the G1/S transition. We genetically modified bacterial artificial chromosomes (BACs; Ciotta et al., 2011; Hutchins et al., 2010) to generate GFP-tagged p27^{Kip1}, CyclinE1, and CyclinA2, under the control of their endogenous promoters. These constructs were used to generate clonal cell lines for this study (Figure S1A). Expression of all three reporters mimicked the localization and expression of their endogenous counterparts (Figures 1 and S1B–S1D), indicating that the GFP tag did not affect their cell-cycle-mediated regulation. Moreover, the GFP tag did not prevent S-phase entry or cell-cycle progression (Figures S1E and S1F) and did not affect protein degradation rates (Figures S1G and S1H; timely degradation of CyclinA2-GFP has been validated in den Elzen and Pines, 2001). Thus, our system accurately reflects Cyclin and p27^{Kip1} expression throughout the G1/S transition.

To correlate Cyclin and CKI expression with the G1/S transition, we co-imaged each GFP tagged protein with LSS2-mKate-PCNA (Figures 1, S1C, and S1D; Movies S1, S2, and S3). Fluorescently labeled PCNA is a robust and well-used marker of DNA replication in mammalian cells (Essers et al., 2005; Held et al., 2010; Leonhardt et al., 2000; Pomerening et al., 2008) and does not interfere with cell-cycle progression in our system. When cells enter S phase, PCNA fluorescence rapidly increases and then switches to a punctate localization (Figures 1A–1D, S1C, and S1D). Since the duration of G1 varies between cells, LSS2-mKate-PCNA localization can act as a marker to pseudo-synchronize cells at the G1/S transition, which we define as time zero (Figures 1A–1C).

We next sought to define the temporal regulation of each of the GFP tagged G1/S regulators throughout the G1/S transition. Consistent with previous observations made in HeLa cell populations by western blotting (Carrano et al., 1999), we found that p27^{Kip1}-GFP is absent after cell division, accumulates rapidly, and is then degraded, starting approximately 5–10 hr after mitotic exit (Figure S1D). Through live single cell imaging, we observed a decelerating pattern of p27^{Kip1}-GFP accumulation during G1 that strongly suggests that p27^{Kip1} degradation is turned off after cell division. By imaging p27^{Kip1}-GFP and LSS2-mKate PCNA in the same cell, we saw that p27^{Kip1}-GFP was very rapidly degraded immediately prior to S-phase entry (Figures 1A and S1D). Western blotting of synchronized cell lysates did not show this rapid degradation because in a cell population cells enter S phase at different rates (Figure S1G), and thus, p27^{Kip1} degradation appears slow.

We observed that CyclinE1-GFP accumulates prior to the G1/S transition and is only degraded after S-phase entry (Figure 1B), consistent with previous reports of CyclinE levels in HeLa cell populations determined by western blotting of lysates from synchronized cells (Dulić et al., 1992; Koff et al., 1992). Single cell imaging also revealed that CyclinE1-GFP levels reach a short plateau immediately after S-phase entry, consistent with the plateau reached by endogenous CyclinE as detected by western blotting (Yuan et al., 2014). Moreover, accumulation of CyclinE1-GFP occurred in an accelerated fashion.

In contrast to CyclinE1-GFP, CyclinA2-GFP remained low during G1, despite the fact that their common transcription factor (E2F) was active during G1 (Figure 1C). Imaging of CyclinA2-GFP showed that CyclinA2 protein only began to accumulate in a linear manner after the G1/S transition (Figure 1C), consistent with previous reports from western blotting of HeLa cell lysates (Pines and Hunter, 1990). This suggests that CyclinA2 is degraded during G1.

Notably, the levels of CyclinE and CyclinA alone do not reveal Cdk2 activity since Cyclin binding alone is not necessarily sufficient to activate Cdks (reviewed in Morgan, 1997). To measure Cdk2 activity, we generated a Cdk2 activity sensor

(D) Stills taken from Movie S4 of HeLa cells stably expressing the CDK2 sensor and LSS2-mKate-PCNA. Graph shows quantification of CDK2 activity from individual cells, aligned to the G1/S transition. Three independent measurements were taken, and nine cells from one experiment are shown. In all cases, each curve is an individual cell. Time on images is shown in minutes and on graphs is shown in hours. Background shading on the graphs shows G1 in red and S phase in green. Scale bars, 10 μm.

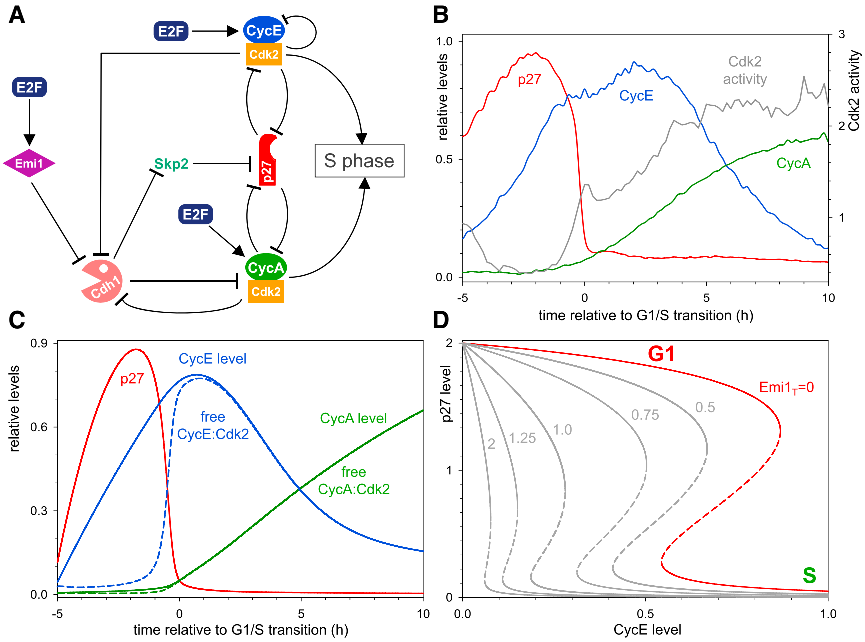

Figure 2. Dynamics of the G1/S Transition in HeLa Cells

(A) Influence diagram of G1/S regulatory network. Both CyclinE and CyclinA, as well as Emi1 (Hsu et al., 2002), are E2F target genes. The network is characterized by multiple, mutual inhibitory network motifs. (1) APC/C^{Cdh1} promotes the degradation of CyclinA, but Cdk2:CyclinA inhibits APC/C activation by Cdh1 (Lukas et al., 1999; Zachariae et al., 1998). (2) p27^{Kip1} inhibits both Cdk2 kinase activities, but Cdk2:Cyclin complexes promote p27^{Kip1} degradation directly and indirectly, thereby creating a coherent feed-forward loop. The direct effect is through phosphorylation of p27 at T187 that targets p27^{Kip1} to the SCF-proteasome system (Montagnoli et al., 1999; Sheaff et al., 1997). The indirect effect is through upregulation of the SCF component, Skp2 by inhibiting its degradation machinery, APC/C^{Cdh1}.

(B) The time courses for each of the cell-cycle reporters and Cdk2 activity was averaged across all cells measured and aligned to S-phase entry (time 0).

(C) Numerical simulation of the G1/S control network with the deterministic version of the model (see Supplemental Experimental Procedures for equations and parameter values).

(D) The steady-state dependence of p27^{Kip1} level on CyclinE and Emi1 (the diagram is computed in the absence of CyclinA since its level is low during the G1/S transition) was calculated as a one-parameter bifurcation diagram with the deterministic version of the model. p27^{Kip1} can settle at high and low levels (solid curves) at low and high CyclinE levels, respectively. The two states are separated by two different thresholds whose values are Emi1 dependent. The G1/S transition is characterized by the drop from the high to low level of p27^{Kip1}.

(CDK2L-GFP), a slightly longer version of the recently published DHB-Ven Cdk2 sensor (see Experimental Procedures; Gu et al., 2004; Spencer et al., 2013). Briefly, this is a fluorescent sensor of Cdk2 activity based on the C-terminal fragment of DNA Helicase B. When phosphorylated by Cdk2, the sensor leaves the nucleus. By measuring the cytoplasmic:nuclear ratio of CDK2L-GFP, we can obtain an estimate of Cdk2 kinase activity from individual cells. First, we validated that CDK2L-GFP was a specific reporter of Cdk2 activity in HeLa cells (Figures S2A–S2I). As judged by our CDK2L-GFP sensor, Cdk2 kinase activity abruptly increased at S-phase entry, followed by a second phase of activity increase that occurs at a relatively slower rate (Figure 1D; Movie S4). We saw similar behavior if we used the DHB-Ven Cdk2 activity sensor (Spencer et al., 2013) in HeLa cells (Figure S2J). The abrupt increase in kinase activity was precisely correlated with the rapid decrease in p27^{Kip1}-GFP (Figures 1A and 1D), suggesting that degradation of p27^{Kip1} may be rate limiting in Cdk2 activation and S-phase entry. Immediately after S-phase entry, we consistently observed a transient decrease in Cdk2 activity (Figures 1D and S2J). In CyclinE1/2-depleted cells, this decrease is more pronounced (Figure 3D). However, what causes this dip in Cdk2 activity is currently not known and warrants further investigation. Taken together, these observations describe key G1/S signaling events in single cells throughout the G1/S transition.

Developing a Dynamical Model of the G1/S Transition

To understand how the observed dynamics of Cyclin, CKI and Cdk2 activity emerge from the underlying molecular network, we next generated a dynamical model of the G1/S transition in human cells based on our live imaging data. Until now, the dynamical data necessary to parametrize such a model have not been available, as the current literature provides information only on the molecular mechanisms controlling the G1/S transition (Kohn, 1999). This G1/S molecular network involves multiple interactions between Cdk2:CyclinA, Cdk2:CyclinE, p27^{Kip1}, and their regulators (Figure 2A).

We have used prior knowledge of the G1/S molecular network to construct a stochastic mathematical model of the G1/S transition (see Supplemental Information for a detailed description of rate expressions). Our model contains a considerable number of kinetic parameters (25) that are largely unknown in the current literature, except for the half-lives of some mRNAs and proteins (Schwanhäusser et al., 2011). The unknown rate constants were estimated by comparing deterministic simulations of the model (Figure 2C) with averaged time courses of pseudo-synchronized cell-cycle regulators (Figures 2B and S3A) in a brute-force approach.

In our model, active E2F leads to an accumulation of CyclinE protein early in G1 (Figures 2C and S3B), in the absence of functional pRb in HeLa cells (Moody and Laimins, 2010; Schwarz et al., 1985). However, CyclinE accumulation is insufficient to drive progression into S phase as Cdk2:CyclinE activity is inhibited by its stoichiometric CKI, p27^{Kip1}, whose degradation, dependent on Cdk2 and SCF^{Skp2} activities (Sheaff et al., 1997), is slow at low Cdk2 activity and low Skp2 levels. Once sufficient CyclinE accumulates, Cdk2:CyclinE activity overcomes p27^{Kip1} inhibition. The activated Cdk2:CyclinE targets p27^{Kip1} for proteasome-dependent degradation through phosphorylation of T187 on p27^{Kip1} (Müller et al., 1997; Sheaff et al., 1997; Vlach et al., 1997). With the degradation of p27^{Kip1}, more Cdk2:CyclinE is released, and p27^{Kip1} degradation is further enhanced. The

fast positive feedback results in the abrupt degradation of $p27^{Kip1}$. After the abrupt degradation of $p27^{Kip1}$, Cdk2:CyclinE kinase becomes activated (Figures 2C and S3B).

In our model, despite the activation of CyclinA protein synthesis, the level of CyclinA is kept low in G1 phase by APC/C^{Cdh1}-mediated degradation (Figures 2C and S3B; Geley et al., 2001; Lukas et al., 1999; Sørensen et al., 2001). Accumulation of CyclinA does not start until inactivation of APC/C^{Cdh1}. At the end of G1, APC/C^{Cdh1} is inactivated due to the accumulation of Emi1 (Hsu et al., 2002; Miller et al., 2006) and phosphorylation of Cdh1 by Cdk2 (Keck et al., 2007; Lukas et al., 1999). This leads to accumulation of Skp2 (Bashir et al., 2004; Wei et al., 2004). The degradation of phosphorylated $p27^{Kip1}$, which is dependent on SCF^{Skp2}-dependent poly-ubiquitylation, is further accelerated by Skp2 accumulation in late G1 (Figure S3C).

All of these events—$p27^{Kip1}$ degradation, Cdk2 activation, and CyclinA accumulation—coincide with the G1/S transition. This leads to the phosphorylation-dependent degradation of CyclinE protein by Cdk2 (Won and Reed, 1996). A linear combination of the model-calculated free Cdk2:Cyclin complexes (Figure 2C) therefore approximates the Cdk2 activity, as measured by our Cdk2 sensor (Figure 2B).

Our model explains the switch-like characteristic of the G1/S transition by mutual inhibition among cell-cycle regulators, with a central role for $p27^{Kip1}$. The abrupt degradation of $p27^{Kip1}$ has a critical threshold and fast protein turnover requirement. In our model, the threshold originates from the antagonistic interaction between $p27^{Kip1}$ and Cdk2:Cyclin complexes, which creates a bistable switch with the following characteristics. (1) The steady-state level of $p27^{Kip1}$ can be either high or low depending on CyclinE levels, but intermediate steady-state levels are unstable (Figure 2D). (2) The transition from a high to low $p27^{Kip1}$ level takes place at a higher threshold of CyclinE than the opposite transition. Therefore, G1/S control possesses the property of hysteresis. (3) The threshold of CyclinE at which transitions occur is dependent on the levels of Emi1 that inhibit APC/C^{Cdh1} and thereby upregulate Skp2 (Figure 2D). At the beginning of the cycle when both CyclinE and Emi1 levels are low, the thresholds are high, $p27^{Kip1}$ is attracted to the upper steady state, and its level rises (red curve in Figure 2D). (4) During G1, E2F-dependent transcription drives the increase in both CyclinE and Emi1 protein levels. Thus, CyclinE and Emi1 could act synergistically to remove $p27^{Kip1}$. CyclinE levels reach the upper threshold at a relatively low level of Emi1 that drives the cell through G1/S into a state of low $p27^{Kip1}$ (Figure S3D). (5) After the G1/S transition, CyclinE levels start to decrease, but the cell stays in the low $p27^{Kip1}$ regime because the thresholds are also decreasing through Emi1 accumulation (Figure S3D). The continuous decrease of the thresholds makes G1/S an irreversible transition in the model.

Critically, our stochastic model captures the observed dynamics of G1/S regulators in HeLa cells (compare Figure S3A with S3B) and thus provides a suitable framework by which to make novel predictions regarding the mechanisms that underlie the dynamical nature of the G1/S transition.

Degradation of $p27^{Kip1}$ Is Driven by Cdk2 Activity

Our model of the G1/S regulatory network attributes the abrupt decrease of $p27^{Kip1}$ to fast protein turnover regulated by direct and indirect double-negative feedback loops between $p27^{Kip1}$ and Cdk2. Specifically, activated Cdk2 promotes $p27^{Kip1}$ degradation. Our model also implies that Cdk2 is required to keep the level of $p27^{Kip1}$ low after G1/S. Inhibition of Cdk2 should lead to re-accumulation of $p27^{Kip1}$ (Figure S3E). To test this prediction, we treated HeLa cells, arrested in early S phase by a thymidine block, with a Cdk1/2 inhibitor. In S-phase-arrested cells, $p27^{Kip1}$ levels are negligible. However, upon addition of Cdk1/2 inhibitor, $p27^{Kip1}$ rapidly re-accumulated (Figure S3F). This rapid re-accumulation of $p27^{Kip1}$ suggests that its synthesis (transcription and translation) is still active during DNA replication. Moreover, the low level of $p27^{Kip1}$ after G1/S transition is indeed maintained by high Cdk2 activity promoting $p27^{Kip1}$ degradation. Therefore, our model correctly predicts that a low level of $p27^{Kip1}$ is maintained throughout S phase by high Cdk activity.

CyclinE Controls the Timing of the G1/S Transition

Stochastic model simulation with reduced levels of residual CyclinE synthesis suggests that the G1/S transition is delayed if CyclinE levels are depleted in G1 (compare the red and gray $p27^{Kip1}$ curves on Figure 3A). However, the rapid degradation of $p27^{Kip1}$ and the abrupt activation of Cdk2 are conserved even after CyclinE depletion. The abruptness of $p27^{Kip1}$ degradation in the absence of CyclinE can be attributed to the mutual antagonism between Cdk2:CyclinA and its inhibitor $p27^{Kip1}$ (Figure 2A). The steady-state level of $p27^{Kip1}$ shows a similar Z-shaped dependence on the levels of CyclinA (not shown). The two CyclinA thresholds, where $p27^{Kip1}$ decreases or rises, are Emi1 dependent because Emi1 inhibits APC/C^{Cdh1} to upregulate Skp2 levels (Figure 3B). The model predicts that the "path" during the G1/S transition is different for control siRNA-treated cells (Figure S3D) and CyclinE-depleted cells (Figure 3B). In control siRNA cells, CyclinE and Emi1 accumulate simultaneously. Since Emi1 is low, APC/C^{Cdh1} is largely active, and Skp2 level is low. Therefore, a large amount of CyclinE is needed (>0.6 arbitrary units [a.u.]) to trigger $p27^{Kip1}$ degradation. In CyclinE-depleted cells, Emi1 accumulates first, leading to APC/C^{Cdh1} inactivation and subsequent accumulation of CyclinA. In the presence of a high level of Emi1, inactive APC/C^{Cdh1} and a high level of Skp2, a low level of CyclinA (approximately 0.2 a.u.) is sufficient to trigger $p27^{Kip1}$ degradation.

Consistent with our model prediction, siRNA-mediated depletion of CyclinE delayed the degradation of $p27^{Kip1}$, activation of Cdk2, and initiation of S phase (compare the gray with the red and black curves on Figures 3C and 3D, respectively; see also Figures S4A and S4B). In addition, the abrupt degradation of $p27^{Kip1}$ and abrupt activation of Cdk2 remained intact after CyclinE depletion (Figures 3C and 3D). Together, our results suggest that although CyclinE is dispensable for the initiation of DNA replication (Geng et al., 2003) CyclinE does play a role in the timing the G1/S transition and thus is rate limiting.

CyclinA Is Dispensable for the Timing of G1/S Transition

Stochastic model simulation with reduced levels of CyclinA suggests no change in the timing of the G1/S transition (compare the red and gray p27 curves on Figure 4A). In addition, the simulation suggested that neither $p27^{Kip1}$ degradation nor Cdk2 activation showed any delay relative to control cells and that these events also kept their close association with the G1/S transition. These

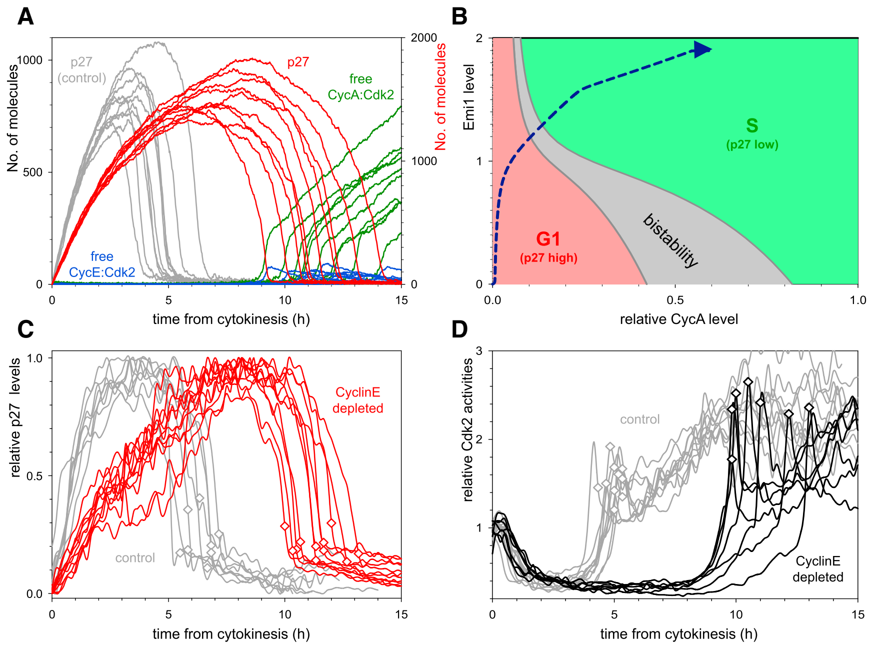

Figure 3. G1/S Transition in CyclinE1,2-Depleted Cells

(A) Stochastic simulations of G1/S transition with 5% residual CyclinE synthesis (with 20-fold increase in mRNA degradation rate). Relative $p27^{Kip1}$ levels and free Cdk2:Cyclin complexes are shown.

(B) The CyclinA thresholds for $p27^{Kip1}$ inactivation and reactivation are plotted as a function of Emi1 levels with the deterministic model. The diagram is divided into three territories: low (green) and high (red) levels of $p27^{Kip1}$ and bistability (gray) where both of these states coexist. The trajectory of cell-cycle progression of CyclinE1/2-depleted cells is shown by a blue dashed curve.

(C and D) Time courses of $p27^{Kip1}$-GFP level (C) and Cdk2 activity (D) in individual CyclinE1/2-depleted (color curves) and control siRNA-treated cells (gray curves). Both time courses are plotted from cell division, and S-phase entry is marked by a diamond on each curve. In (C), three independent experiments were conducted, and 8 (control siRNA) and 11 (CyclinE1/2 siRNA) cells are shown from one experiment. In (D), two independent experiments were conducted, and 10 (control siRNA) and 7 (CyclinE1/2 siRNA) cells are shown from one experiment.

simulation results are consistent with our experimental testing, in which CyclinA is depleted by siRNA (compare the gray with the red and black curves on Figures 4B and 4C, respectively; see also Figures S4A and S4C).

Since CyclinA does not control the timing of the G1/S transition, the observation that different cells enter S phase at different times is unlikely due to their different levels of CyclinA. Based on our stochastic simulations (Figure 4A), we predict that the stochastic expression of other G1/S regulators (for example CyclinE or Emi1) is responsible for the variability in timing of the G1/S transition in CyclinA-deficient cells.

Emi1 Ensures an Irreversible G1/S Transition

One way to ensure that DNA is replicated once and only once per cell cycle is to make the G1/S transition difficult to reverse. Intuitively, we might predict that by binding and activating Cdk2 CyclinA could maintain an S-phase state after CyclinE is degraded. However, our model simulations predict that CyclinA depletion in S-phase cells is not sufficient to trigger re-accumulation of $p27^{Kip1}$ and thus reset cells into G1. In contrast, our model suggests that Emi1 is both necessary and sufficient to maintain the post-G1 state. Specifically, our model predicts that increasing levels of Emi1 during S phase decrease the amount of CyclinE required to maintain $p27^{Kip1}$ degradation (Figures 2D and S3D). If we remove Emi1 from S-phase cells in the model, APC/C^{Cdh1} is activated, CyclinA and Skp2 are degraded, and $p27^{Kip1}$ and CyclinE re-accumulate. Later, increasing CyclinE causes a second round of $p27^{Kip1}$ degradation, and the cells could re-enter S phase once again (Figures 5A and S5A).

To test our model prediction, we synchronized cells in early S phase (with a double thymidine block) and depleted Emi1 by RNA interference during release from the first thymidine block, such that Emi1 would be depleted in the next cell cycle (Figure S5B; see Supplemental Experimental Procedures). Control siRNA-treated cells undergo mitotic cycling, confirmed by fluorescence-activated cell sorting (FACS) (Figure 5B) and the temporal patterns of cell-cycle regulators (see Figures S5C–S5F). In contrast, Emi1 depletion leads to re-replication of DNA, as previously reported (Figure 5B; Di Fiore and Pines, 2007; Machida and Dutta, 2007). This suggests that Emi1 is required to maintain an irreversible G1/S transition.

Consistent with our model prediction, the experimental data clearly show a short-lived increase then degradation of CyclinA2-GFP (Figure 5C), followed by transient accumulation of $p27^{Kip1}$-GFP (Figure 5D), and re-accumulation of CyclinE1-GFP (Figure 5E). This accumulation of CyclinE1 protein is likely due to a combination of a low level of Cdk2 activity since CyclinE1-GFP is stabilized when Cdk2 activity is inhibited (Figure S6A) and high E2F activity as cells return to a G1-like state. Direct measurements of Cdk2 activity in Emi1-depleted cells show an initial small increase in kinase activity after release from the thymidine block, followed by a decrease. In the majority of cells, this decrease is small and is followed by a plateau in Cdk2 levels at an intermediate level of between 1 and 1.5 (Figure 5F). However, in some cells, this dip in Cdk2 activity is more pronounced. The activity of Cdk2 in any individual Emi1-depleted cell will depend on (1) the level of Emi1 depletion and (2) the relative amounts of CyclinE and CyclinA when Emi1 is depleted in that cell. Therefore, cells with a larger dip in Cdk2 activity may reflect cells with a better Emi1 depletion and/or a higher ratio of CyclinA to CyclinE when Emi1 is depleted.

This begs the question of how DNA re-replicates after Emi1 depletion. Notably, we do not observe endoreduplication after Emi1 depletion (Figure 5B; no discrete peak is seen at 8n; at 200K on the x axis). If we image PCNA dynamics in cells depleted of Emi1, we can observe nuclei switching between G1 and S in the absence of mitosis and nuclei increasing in size (Figure S6B;

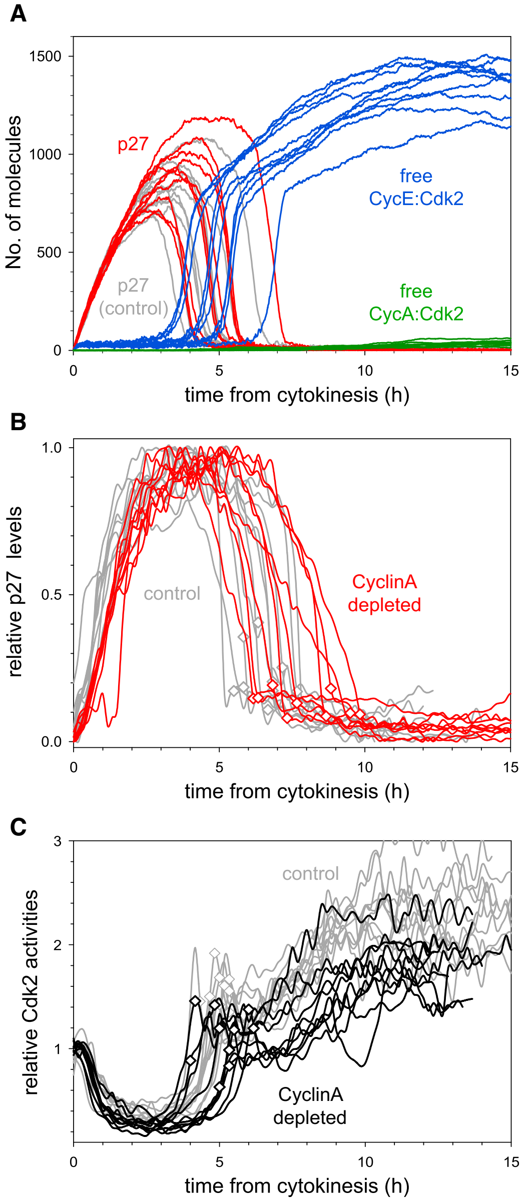

Figure 4. G1/S Transition in CyclinA-Depleted Cells

(A) Stochastic simulations of the G1/S transition with 5% residual CyclinA synthesis (with 20-fold increase in mRNA degradation rate).

(B and C) Time courses of p27^{Kip1}-GFP level (B) and Cdk2 activity (C) in individual CyclinA2-depleted (color curves) and control siRNA-treated cells (gray curves). Both time courses are plotted from cell division, and S-phase entry is marked by a diamond on each curve. In (B), three independent experiments were conducted, and ten (control siRNA) and nine (CyclinA2 siRNA) cells are shown from one experiment. In (C), two independent experiments were conducted, and ten (control siRNA) and ten (CyclinA2 siRNA) cells are shown from one experiment.

Movies S5 and S6). However, PCNA foci in Emi1-depleted cells are not discrete, as in control siRNA-treated cells, and S-phase entry is more noticeable by changes in the "texture" of PCNA localization throughout the nucleus (Movie S6). This implies that after Emi1 depletion, cells do not progress through "early," "mid," and "late" DNA replication stages, but instead likely sit at the boundary of G1 and S, refiring some DNA replication origins. Loss of Geminin after Emi1 depletion (Di Fiore and Pines, 2007; Machida and Dutta, 2007) would permit origin refiring. This is also consistent with recent data suggesting uncontrolled reactivation of replication origins after Emi1 depletion (Neelsen et al., 2013). Altogether, our data suggest that S-phase cells depleted of Emi1 can transiently return to a G1 state (low CyclinA, high p27^{Kip1}, accumulating CyclinE) before recommitting to DNA synthesis, supporting our model prediction that Emi1 maintains the irreversibility of the G1/S transition.

DISCUSSION

Our model suggests that the abrupt degradation of p27^{Kip1} is the result of a rapid proteolytic double-negative feedback loop between Cdk2:CyclinE and p27^{Kip1}. This fast degradation of p27^{Kip1} ensures a rapid activation of Cdk2 and immediate S-phase entry. We propose that p27^{Kip1} degradation is rate limiting in S-phase entry in HeLa cells. Indeed, this is consistent with previous reports using siRNA to deplete p27^{Kip1} (Sabile et al., 2006; Yuan et al., 2014). Thus, even though the proteolytic double-negative feedback loop between a Cdk:Cyclin complex and a CKI is embedded within a more complex architecture in HeLa cells than in yeast, it is sufficient to explain the temporally abrupt switching between two discrete cell-cycle states.

While the feedback between Cdk2:CyclinE and p27^{Kip1} is sufficient to explain the switch-like transition between G1 and S phases, our model predicts that the transition is made globally irreversible by Emi1 accumulation. In support of this, we observe that single S-phase cells depleted of Emi1 re-express p27^{Kip1}, accumulate CyclinE, degrade CyclinA, and appear to engage in simultaneous licensing and firing of replication origins, which are normally temporally distinct behaviors (Figure 5B). It is also formally possible that Emi1-depleted cells are activating a checkpoint that leads to the stabilization of p27^{Kip1}. However, since p27^{Kip1} expression is not sustained in the presence of continuous Emi1 depletion and we observe DNA re-replication, we do not think that this is the case.

In marked contrast to MCF10A and fibroblast cell lines, where populations exhibit heterogeneous Cdk2 dynamics and exist in either Cdk2^{high} or Cdk2^{low} states (Spencer et al., 2013), HeLa cells are homogeneous in terms of their pattern of Cdk2 activity. HeLa cells lack restriction point control due to expression of E6 and E7 proteins from integrated HPV-18, which inactivate p53 and pRb, respectively (Boshart et al., 1984; Butz et al., 1995) and also overexpress c-Myc (Dürst et al., 1987; Lazo et al., 1989). By extending our model to include pRb control over E2F activity, the differences between HeLa versus MCF10A and fibroblast cell lines can be explained. This suggests that the model may be generalizable to explain some of the differences between cancer and non-transformed cells. In non-transformed cells growing in the presence of intermediate levels of mitogens, the decision between proliferation and G1 arrest is made at the end of the previous cycle (Overton et al., 2014; Spencer et al., 2013). pRb phosphorylation by E2F-induced Cdk2:Cyclin complexes creates additional amplification loops within the network (Figure 6A), with low and high Cdk2:CyclinE activity states (Figure 6B). Whether dividing cells adopt a Cdk2^{high} or Cdk2^{low} fate depends on the mutual antagonism

OPEN ACCESS
Cell Press

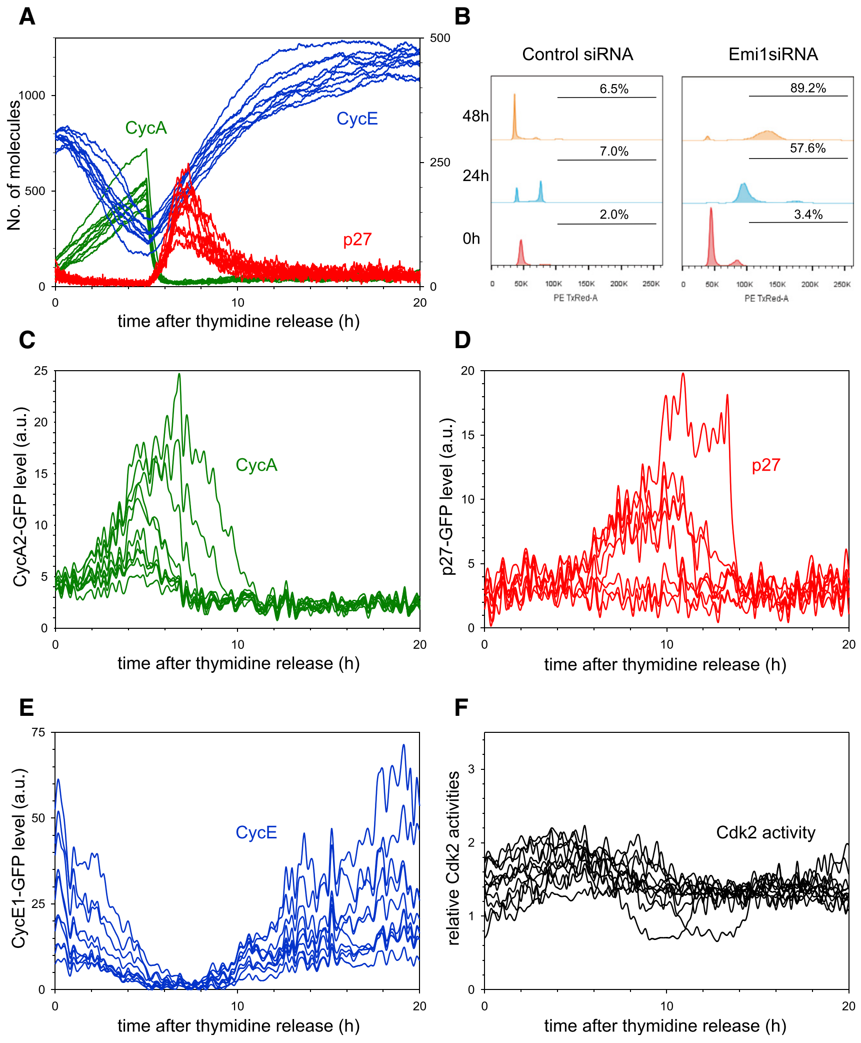

Figure 5. Emi1 Is Required for an Irreversible G1/S Transition

(A) Stochastic simulation of Emi1 depletion. The initial state of the model corresponds to an S-phase cell (CyclinA increasing, CyclinE decreasing, and $p27^{Kip1}$ level is low, see Figure 2C). At $t = 5h$, the level and rate of synthesis of Emi1 were reduced by 90%, causing APC/C^{Cdh1} reactivation, loss of CyclinA, and accumulation of $p27^{Kip1}$ and CyclinE. Later, the re-accumulation of CyclinE promotes the re-degradation of $p27^{Kip1}$.

(B) DNA re-replication after Emi1 depletion. FACS plots of control (left) and Emi1-depleted (right) cells at 0, 24, and 48 hr after release from thymidine. Numbers shown on each graph are the percentage of cells with DNA > 4n.

(C) Experimental measurement of CyclinA2-GFP in Emi1-depleted cells. CyclinA2-GFP levels continue to increase at the beginning of the experiment, possibly since Emi1 is still functional. Later, CyclinA2-GFP levels decrease and remain low due to reactivation of APC/C^{Cdh1} in the absence of Emi1. Two independent measurements were taken, and ten cells from one experiment are shown.

(D) Experimental measurement of $p27^{Kip1}$-GFP in Emi1-depleted cells. $p27^{Kip1}$-GFP is initially low and then shows a transient increase in some cells. Two independent measurements were taken, and eight cells from one experiment are shown.

(E) Experimental measurement of CyclinE1-GFP in Emi1-depleted cell. CyclinE1-GFP initially decreases and then continues to accumulate for the duration of the experiment. Two independent measurements were taken, and ten cells from one experiment are shown.

(F) Quantification of Cdk2 activity in Emi1-depleted cells. Two cells show a significant dip in Cdk2 activity. Other cells show a more moderate decrease. In all cases, individual curves represent individual cells. Three independent measurements were taken, and 11 cells from one experiment are shown.

between CKIs and the residual Cdk activity after degradation of CyclinA and CyclinB during mitosis. Cdk2:CyclinE activity is the most significant in this context, and if it is high relative to CKI-mediated inhibition, cells progress into S phase (Figure 6C). In the opposite scenario, high CKI and low Cdk2:CyclinE activity leads to pRb-dephosphorylation, E2F inactivation, and G1 arrest (Figure 6D).

Perhaps because HeLa cells lack functional pRb, they exist in a single signaling state that leads to S-phase commitment in all cells. We suggest that this homogeneity may be an important aspect in oncogenesis, as the lack of bifurcation into CDK^{high} or CDK^{low} states would ensure that the majority of cells in a tumor have a high probability of proliferating, resulting in increased tumor fitness. In contrast, if a subpopulation of a tumor frequently entered a $CDK2^{low}$ state, this would decrease the fitness of the population as a whole. However, evolving homogeneity in S-phase commitment during oncogenesis may come at a price, as the signaling networks regulating the G1/S transition exist in a single (attractor) region in signaling state space and thus are less robust to inhibition of components necessary for that state. Homogeneity in the signaling state of the G1/S network across cancer cells may explain the somewhat counterintuitive observations that some cancer cells are more dependent on key cell-cycle regulators, such as Cdk2 and CyclinE, than non-transformed cells (Martín et al., 2005; Tetsu and McCormick, 2003; van den Heuvel and Harlow, 1993).

In future studies, it will be important to determine whether the systems that regulate G1/S are similar in other cancer cell models that lack restriction point control in order to gain mechanistic insight into cell-cycle regulation. Ultimately, different patients may be stratified not only on the basis of genotypic differences, but also on predicted differences in both the architecture and dynamics of their regulatory networks.

EXPERIMENTAL PROCEDURES

Generation of Fluorescently Tagged BACs and Reporters

BACs used in this study were ordered from BACPAC Resource center (CHORI). BACs used are $p27^{Kip1}$ RP11-70N14, CCNE1 RP11-345J21, and CCNA2 RP11-768B18. All BACs were verified by PCR using verification primers as listed on the Mitocheck website (http://www.mitocheck.org). Proteins of interest were tagged using BAC Recombineering at their C-termini

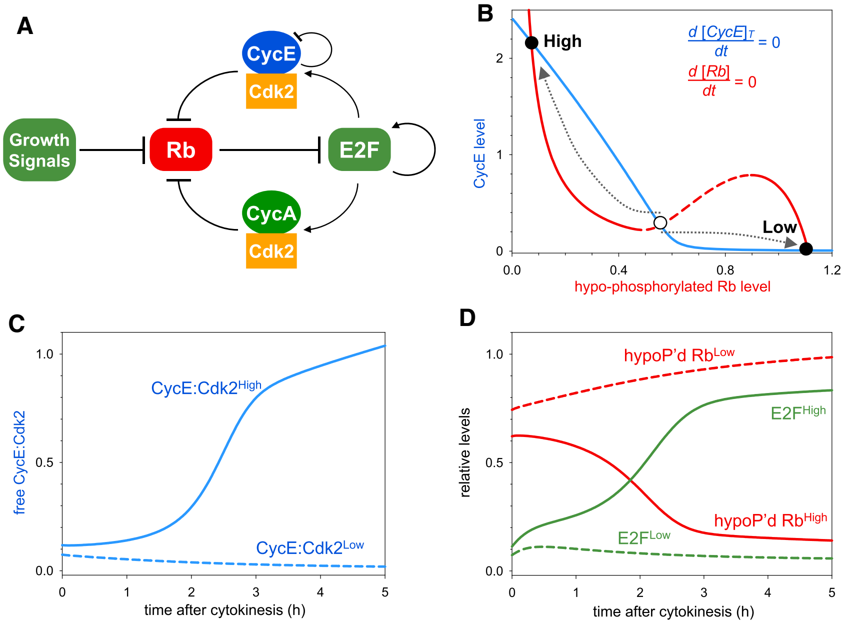

Figure 6. G1 Progression in Cells with an Intact Restriction Point

(A) G1 control in cells with an intact restriction point including pRb control over E2F activity, as well E2F autoregulation.

(B) The steady-state levels of CyclinE (blue) and pRb (red) in the extended model are plotted against each other. Low hypo-phosphorylated pRb corresponds to high E2F activity. These balance curves of CyclinE and hypo-phosphorylated pRb create two qualitatively different steady states (high CyclinE, low pRb labeled "High" and low CyclinE, high pRb labeled "Low") after cell division. The existence of two different cellular states (fates) is largely dependent on the inverse N-shaped characteristic of the pRb-balance curve (red) caused by the antagonism between Cdk2:CyclinE and CKI. In the absence of CKI, the red curve becomes a hyperbole, and the bistable regime is much reduced. The "dividing the way" behavior is illustrated by two trajectories (dotted arrows).

(C and D) Temporal evolution of free Cdk2:CyclinE (C) as well as E2F and hypo-phosphorylated pRb (D) in a cell approaching high and low Cdk2 activity states.

with localization and affinity purification (LAP) tags that contain GFP and an affinity purification tag (Poser et al., 2008). Primers used to tag BACs were those listed on the Mitocheck BACFinder website (http://www.mitocheck.org/cgi-bin/BACfinder). Successful recombination and tagging of genes was confirmed by PCR followed by DNA sequencing.

PCNA was tagged at the N terminus with the modified LSS2-mKate fluorophore to generate LSS2-mKate-PCNA (Piatkevich et al., 2010).

To measure CDK2 activity, we used a similar strategy, now described in Spencer et al. (2013). We cloned the C-terminal PSLD region of Human DNA Helicase B (DHB; 957-1087 amino acids of DHB) into pIRES-GFP Puro3 to generate CDK2L-GFP. Note that this sensor is slightly longer than that used in Spencer et al. and is similar to the one first described in Gu et al. (2004).

Live Cell Imaging

Live cell imaging was performed using a Zeiss LSM710 confocal, with a 40× oil objective, NA 1.3, or a High-content Opera Spinning disk confocal microscope (PerkinElmer), with a 40× water objective, NA 0.9. All live cell imaging was carried out in a humidified environmental chamber maintained at 37°C and 5% CO_2.

Quantification of Reporters

All imaging data was quantified in Volocity (PerkinElmer). To find dynamic changes in GFP intensities, cell nuclei were tracked using the LSS2-mKate-PCNA signal and the Shortest Path algorithm. A maximum distance of 9 μm between nuclei in consecutive timeframes was used to eliminate erroneous tracks. For $p27^{Kip1}$-GFP, CyclinE1-GFP, and CyclinA2-GFP, nuclear levels of protein were quantified since all three proteins had an exclusively nuclear localization during G1 and S. In each cell, the average value of pixel intensities was calculated, and average background fluorescence was subtracted. All fluorescence intensities were normalized to peak levels. Timing of S-phase entry was determined manually from PCNA fluorescence images. Specifically, S-phase entry is defined by a sharp increase in PCNA intensity, often coinciding with the appearance of nucleoli.

For CDK2L-GFP and DHB-Ven quantification, nuclear intensity was measured by taking a region of interest (ROI) inside the nucleus, and cytoplasmic intensity was measured as the ring region around the nucleus. Cdk2 activity was then measured as the ratio between the ring GFP intensity to the nuclear GFP intensity.

Additional experimental procedures are provided in Supplemental Information.

SUPPLEMENTAL INFORMATION

Supplemental Information includes Supplemental Experimental Procedures, six figures, and six movies and can be found with this article online at http://dx.doi.org/10.1016/j.cels.2016.01.001.

AUTHOR CONTRIBUTIONS

A.R.B. designed and performed experiments. A.R.B., F.S.H., T.Z., C.B., and B.N. analyzed the data. T.Z., F.S.H., and B.N. created the mathematical model. A.R.B., F.S.H., T.Z., C.B., and B.N. wrote the paper.

ACKNOWLEDGMENTS

We thank Faraz Mardakheh and Rebecca Wilson for technical help. We thank Francis Barr and Jonathon Pines for critical reading of the manuscript. We thank Ellen Fanning (Vanderbilt-Ingram Cancer Center), Cristina Cardoso (Technische Universitat), Vladislav Verkhusha (Albert Einstein College of Medicine), Tobias Meyer (Stanford University), and Spiros Linardopoulos (Institute of Cancer Research) for reagents. The groups of C.B. and B.N. are supported by BBSRC Strategic LoLa grant (BB/MM00354X/1).

Received: June 29, 2015
Revised: November 9, 2015
Accepted: January 4, 2016
Published: January 27, 2016

REFERENCES

Asghar, U., Witkiewicz, A.K., Turner, N.C., and Knudsen, E.S. (2015). The history and future of targeting cyclin-dependent kinases in cancer therapy. Nat. Rev. Drug Discov. *14*, 130–146.

Bashir, T., Dorrello, N.V., Amador, V., Guardavaccaro, D., and Pagano, M. (2004). Control of the SCF(Skp2-Cks1) ubiquitin ligase by the APC/C(Cdh1) ubiquitin ligase. Nature *428*, 190–193.

Bertoli, C., Skotheim, J.M., and de Bruin, R.A. (2013). Control of cell cycle transcription during G1 and S phases. Nat. Rev. Mol. Cell Biol. *14*, 518–528.

Boshart, M., Gissmann, L., Ikenberg, H., Kleinheinz, A., Scheurlen, W., and zur Hausen, H. (1984). A new type of papillomavirus DNA, its presence in genital

OPEN ACCESS
CellPress

cancer biopsies and in cell lines derived from cervical cancer. EMBO J. *3*, 1151–1157.

Butz, K., Shahabeddin, L., Geisen, C., Spitkovsky, D., Ullmann, A., and Hoppe-Seyler, F. (1995). Functional p53 protein in human papillomavirus-positive cancer cells. Oncogene *10*, 927–936.

Carrano, A.C., Eytan, E., Hershko, A., and Pagano, M. (1999). SKP2 is required for ubiquitin-mediated degradation of the CDK inhibitor p27. Nat. Cell Biol. *1*, 193–199.

Charvin, G., Oikonomou, C., Siggia, E.D., and Cross, F.R. (2010). Origin of irreversibility of cell cycle start in budding yeast. PLoS Biol. *8*, e1000284.

Chen, K.C., Csikasz-Nagy, A., Gyorffy, B., Val, J., Novak, B., and Tyson, J.J. (2000). Kinetic analysis of a molecular model of the budding yeast cell cycle. Mol. Biol. Cell *11*, 369–391.

Chu, I.M., Hengst, L., and Slingerland, J.M. (2008). The Cdk inhibitor p27 in human cancer: prognostic potential and relevance to anticancer therapy. Nat. Rev. Cancer *8*, 253–267.

Ciotta, G., Hofemeister, H., Maresca, M., Fu, J., Sarov, M., Anastassiadis, K., and Stewart, A.F. (2011). Recombineering BAC transgenes for protein tagging. Methods *53*, 113–119.

Cross, F.R., Archambault, V., Miller, M., and Klovstad, M. (2002). Testing a mathematical model of the yeast cell cycle. Mol. Biol. Cell *13*, 52–70.

Cross, F.R., Buchler, N.E., and Skotheim, J.M. (2011). Evolution of networks and sequences in eukaryotic cell cycle control. Philos. Trans. R. Soc. Lond. B Biol. Sci. *366*, 3532–3544.

den Elzen, N., and Pines, J. (2001). Cyclin A is destroyed in prometaphase and can delay chromosome alignment and anaphase. J. Cell Biol. *153*, 121–136.

Di Fiore, B., and Pines, J. (2007). Emi1 is needed to couple DNA replication with mitosis but does not regulate activation of the mitotic APC/C. J. Cell Biol. *177*, 425–437.

Dulić, V., Lees, E., and Reed, S.I. (1992). Association of human cyclin E with a periodic G1-S phase protein kinase. Science *257*, 1958–1961.

Dürst, M., Croce, C.M., Gissmann, L., Schwarz, E., and Huebner, K. (1987). Papillomavirus sequences integrate near cellular oncogenes in some cervical carcinomas. Proc. Natl. Acad. Sci. USA *84*, 1070–1074.

Essers, J., Theil, A.F., Baldeyron, C., van Cappellen, W.A., Houtsmuller, A.B., Kanaar, R., and Vermeulen, W. (2005). Nuclear dynamics of PCNA in DNA replication and repair. Mol. Cell. Biol. *25*, 9350–9359.

Fero, M.L., Rivkin, M., Tasch, M., Porter, P., Carow, C.E., Firpo, E., Polyak, K., Tsai, L.H., Broudy, V., Perlmutter, R.M., et al. (1996). A syndrome of multiorgan hyperplasia with features of gigantism, tumorigenesis, and female sterility in p27(Kip1)-deficient mice. Cell *85*, 733–744.

Geley, S., Kramer, E., Gieffers, C., Gannon, J., Peters, J.M., and Hunt, T. (2001). Anaphase-promoting complex/cyclosome-dependent proteolysis of human cyclin A starts at the beginning of mitosis and is not subject to the spindle assembly checkpoint. J. Cell Biol. *153*, 137–148.

Geng, Y., Eaton, E.N., Picón, M., Roberts, J.M., Lundberg, A.S., Gifford, A., Sardet, C., and Weinberg, R.A. (1996). Regulation of cyclin E transcription by E2Fs and retinoblastoma protein. Oncogene *12*, 1173–1180.

Geng, Y., Yu, Q., Sicinska, E., Das, M., Schneider, J.E., Bhattacharya, S., Rideout, W.M., Bronson, R.T., Gardner, H., and Sicinski, P. (2003). Cyclin E ablation in the mouse. Cell *114*, 431–443.

Gu, J., Xia, X., Yan, P., Liu, H., Podust, V.N., Reynolds, A.B., and Fanning, E. (2004). Cell cycle-dependent regulation of a human DNA helicase that localizes in DNA damage foci. Mol. Biol. Cell *15*, 3320–3332.

Held, M., Schmitz, M.H., Fischer, B., Walter, T., Neumann, B., Olma, M.H., Peter, M., Ellenberg, J., and Gerlich, D.W. (2010). CellCognition: time-resolved phenotype annotation in high-throughput live cell imaging. Nat. Methods *7*, 747–754.

Hershko, D.D. (2010). Cyclin-dependent kinase inhibitor p27 as a prognostic biomarker and potential cancer therapeutic target. Future Oncol. *6*, 1837–1847.

Hsu, J.Y., Reimann, J.D., Sørensen, C.S., Lukas, J., and Jackson, P.K. (2002). E2F-dependent accumulation of hEmi1 regulates S phase entry by inhibiting APC(Cdh1). Nat. Cell Biol. *4*, 358–366.

Hutchins, J.R., Toyoda, Y., Hegemann, B., Poser, I., Hériché, J.K., Sykora, M.M., Augsburg, M., Hudecz, O., Buschhorn, B.A., Bulkescher, J., et al. (2010). Systematic analysis of human protein complexes identifies chromosome segregation proteins. Science *328*, 593–599.

Johnson, A., and Skotheim, J.M. (2013). Start and the restriction point. Curr. Opin. Cell Biol. *25*, 717–723.

Keck, J.M., Summers, M.K., Tedesco, D., Ekholm-Reed, S., Chuang, L.C., Jackson, P.K., and Reed, S.I. (2007). Cyclin E overexpression impairs progression through mitosis by inhibiting APC(Cdh1). J. Cell Biol. *178*, 371–385.

Kiyokawa, H., Kineman, R.D., Manova-Todorova, K.O., Soares, V.C., Hoffman, E.S., Ono, M., Khanam, D., Hayday, A.C., Frohman, L.A., and Koff, A. (1996). Enhanced growth of mice lacking the cyclin-dependent kinase inhibitor function of p27(Kip1). Cell *85*, 721–732.

Koff, A., Giordano, A., Desai, D., Yamashita, K., Harper, J.W., Elledge, S., Nishimoto, T., Morgan, D.O., Franza, B.R., and Roberts, J.M. (1992). Formation and activation of a cyclin E-cdk2 complex during the G1 phase of the human cell cycle. Science *257*, 1689–1694.

Kohn, K.W. (1999). Molecular interaction map of the mammalian cell cycle control and DNA repair systems. Mol. Biol. Cell *10*, 2703–2734.

Kõivomägi, M., Valk, E., Venta, R., Iofik, A., Lepiku, M., Balog, E.R., Rubin, S.M., Morgan, D.O., and Loog, M. (2011). Cascades of multisite phosphorylation control Sic1 destruction at the onset of S phase. Nature *480*, 128–131.

Lazo, P.A., DiPaolo, J.A., and Popescu, N.C. (1989). Amplification of the integrated viral transforming genes of human papillomavirus 18 and its 5′-flanking cellular sequence located near the myc protooncogene in HeLa cells. Cancer Res. *49*, 4305–4310.

Leonhardt, H., Rahn, H.P., Weinzierl, P., Sporbert, A., Cremer, T., Zink, D., and Cardoso, M.C. (2000). Dynamics of DNA replication factories in living cells. J. Cell Biol. *149*, 271–280.

Lukas, C., Sørensen, C.S., Kramer, E., Santoni-Rugiu, E., Lindeneg, C., Peters, J.M., Bartek, J., and Lukas, J. (1999). Accumulation of cyclin B1 requires E2F and cyclin-A-dependent rearrangement of the anaphase-promoting complex. Nature *401*, 815–818.

Machida, Y.J., and Dutta, A. (2007). The APC/C inhibitor, Emi1, is essential for prevention of rereplication. Genes Dev. *21*, 184–194.

Martín, A., Odajima, J., Hunt, S.L., Dubus, P., Ortega, S., Malumbres, M., and Barbacid, M. (2005). Cdk2 is dispensable for cell cycle inhibition and tumor suppression mediated by p27(Kip1) and p21(Cip1). Cancer Cell *7*, 591–598.

Miller, J.J., Summers, M.K., Hansen, D.V., Nachury, M.V., Lehman, N.L., Loktev, A., and Jackson, P.K. (2006). Emi1 stably binds and inhibits the anaphase-promoting complex/cyclosome as a pseudosubstrate inhibitor. Genes Dev. *20*, 2410–2420.

Montagnoli, A., Fiore, F., Eytan, E., Carrano, A.C., Draetta, G.F., Hershko, A., and Pagano, M. (1999). Ubiquitination of p27 is regulated by Cdk-dependent phosphorylation and trimeric complex formation. Genes Dev. *13*, 1181–1189.

Moody, C.A., and Laimins, L.A. (2010). Human papillomavirus oncoproteins: pathways to transformation. Nat. Rev. Cancer *10*, 550–560.

Morgan, D.O. (1997). Cyclin-dependent kinases: engines, clocks, and microprocessors. Annu. Rev. Cell Dev. Biol. *13*, 261–291.

Müller, D., Bouchard, C., Rudolph, B., Steiner, P., Stuckmann, I., Saffrich, R., Ansorge, W., Huttner, W., and Eilers, M. (1997). Cdk2-dependent phosphorylation of p27 facilitates its Myc-induced release from cyclin E/cdk2 complexes. Oncogene *15*, 2561–2576.

Nakayama, K., Ishida, N., Shirane, M., Inomata, A., Inoue, T., Shishido, N., Horii, I., Loh, D.Y., and Nakayama, K. (1996). Mice lacking p27(Kip1) display increased body size, multiple organ hyperplasia, retinal dysplasia, and pituitary tumors. Cell *85*, 707–720.

Nash, P., Tang, X., Orlicky, S., Chen, Q., Gertler, F.B., Mendenhall, M.D., Sicheri, F., Pawson, T., and Tyers, M. (2001). Multisite phosphorylation of a CDK inhibitor sets a threshold for the onset of DNA replication. Nature *414*, 514–521.

Neelsen, K.J., Zanini, I.M., Mijic, S., Herrador, R., Zellweger, R., Ray Chaudhuri, A., Creavin, K.D., Blow, J.J., and Lopes, M. (2013). Deregulated origin licensing leads to chromosomal breaks by rereplication of a gapped DNA template. Genes Dev. *27*, 2537–2542.

Ohtani, K., DeGregori, J., and Nevins, J.R. (1995). Regulation of the cyclin E gene by transcription factor E2F1. Proc. Natl. Acad. Sci. USA *92*, 12146–12150.

Overton, K.W., Spencer, S.L., Noderer, W.L., Meyer, T., and Wang, C.L. (2014). Basal p21 controls population heterogeneity in cycling and quiescent cell cycle states. Proc. Natl. Acad. Sci. USA *111*, E4386–E4393.

Piatkevich, K.D., Hulit, J., Subach, O.M., Wu, B., Abdulla, A., Segall, J.E., and Verkhusha, V.V. (2010). Monomeric red fluorescent proteins with a large Stokes shift. Proc. Natl. Acad. Sci. USA *107*, 5369–5374.

Pines, J., and Hunter, T. (1990). Human cyclin A is adenovirus E1A-associated protein p60 and behaves differently from cyclin B. Nature *346*, 760–763.

Pomerening, J.R., Ubersax, J.A., and Ferrell, J.E., Jr. (2008). Rapid cycling and precocious termination of G1 phase in cells expressing CDK1AF. Mol. Biol. Cell *19*, 3426–3441.

Poser, I., Sarov, M., Hutchins, J.R., Hériché, J.K., Toyoda, Y., Pozniakovsky, A., Weigl, D., Nitzsche, A., Hegemann, B., Bird, A.W., et al. (2008). BAC TransgeneOmics: a high-throughput method for exploration of protein function in mammals. Nat. Methods *5*, 409–415.

Sabile, A., Meyer, A.M., Wirbelauer, C., Hess, D., Kogel, U., Scheffner, M., and Krek, W. (2006). Regulation of p27 degradation and S-phase progression by Ro52 RING finger protein. Mol. Cell. Biol. *26*, 5994–6004.

Scaltriti, M., Eichhorn, P.J., Cortés, J., Prudkin, L., Aura, C., Jiménez, J., Chandarlapaty, S., Serra, V., Prat, A., Ibrahim, Y.H., et al. (2011). Cyclin E amplification/overexpression is a mechanism of trastuzumab resistance in HER2+ breast cancer patients. Proc. Natl. Acad. Sci. USA *108*, 3761–3766.

Schwanhäusser, B., Busse, D., Li, N., Dittmar, G., Schuchhardt, J., Wolf, J., Chen, W., and Selbach, M. (2011). Global quantification of mammalian gene expression control. Nature *473*, 337–342.

Schwarz, E., Freese, U.K., Gissmann, L., Mayer, W., Roggenbuck, B., Stremlau, A., and zur Hausen, H. (1985). Structure and transcription of human papillomavirus sequences in cervical carcinoma cells. Nature *314*, 111–114.

Schwob, E., Böhm, T., Mendenhall, M.D., and Nasmyth, K. (1994). The B-type cyclin kinase inhibitor p40SIC1 controls the G1 to S transition in S. cerevisiae. Cell *79*, 233–244.

Sheaff, R.J., Groudine, M., Gordon, M., Roberts, J.M., and Clurman, B.E. (1997). Cyclin E-CDK2 is a regulator of p27Kip1. Genes Dev. *11*, 1464–1478.

Sherr, C.J., and Roberts, J.M. (1999). CDK inhibitors: positive and negative regulators of G1-phase progression. Genes Dev. *13*, 1501–1512.

Skotheim, J.M., Di Talia, S., Siggia, E.D., and Cross, F.R. (2008). Positive feedback of G1 cyclins ensures coherent cell cycle entry. Nature *454*, 291–296.

Sørensen, C.S., Lukas, C., Kramer, E.R., Peters, J.M., Bartek, J., and Lukas, J. (2001). A conserved cyclin-binding domain determines functional interplay between anaphase-promoting complex-Cdh1 and cyclin A-Cdk2 during cell cycle progression. Mol. Cell. Biol. *21*, 3692–3703.

Spencer, S.L., Cappell, S.D., Tsai, F.C., Overton, K.W., Wang, C.L., and Meyer, T. (2013). The proliferation-quiescence decision is controlled by a bifurcation in CDK2 activity at mitotic exit. Cell *155*, 369–383.

Tetsu, O., and McCormick, F. (2003). Proliferation of cancer cells despite CDK2 inhibition. Cancer Cell *3*, 233–245.

van den Heuvel, S., and Harlow, E. (1993). Distinct roles for cyclin-dependent kinases in cell cycle control. Science *262*, 2050–2054.

Verdugo, A., Vinod, P.K., Tyson, J.J., and Novak, B. (2013). Molecular mechanisms creating bistable switches at cell cycle transitions. Open Biol. *3*, 120179.

Vlach, J., Hennecke, S., and Amati, B. (1997). Phosphorylation-dependent degradation of the cyclin-dependent kinase inhibitor p27. EMBO J. *16*, 5334–5344.

Wei, W., Ayad, N.G., Wan, Y., Zhang, G.J., Kirschner, M.W., and Kaelin, W.G., Jr. (2004). Degradation of the SCF component Skp2 in cell-cycle phase G1 by the anaphase-promoting complex. Nature *428*, 194–198.

Weinberg, R.A. (1995). The retinoblastoma protein and cell cycle control. Cell *81*, 323–330.

Won, K.A., and Reed, S.I. (1996). Activation of cyclin E/CDK2 is coupled to site-specific autophosphorylation and ubiquitin-dependent degradation of cyclin E. EMBO J. *15*, 4182–4193.

Yang, X., Lau, K.Y., Sevim, V., and Tang, C. (2013). Design principles of the yeast G1/S switch. PLoS Biol. *11*, e1001673.

Yao, G., Lee, T.J., Mori, S., Nevins, J.R., and You, L. (2008). A bistable Rb-E2F switch underlies the restriction point. Nat. Cell Biol. *10*, 476–482.

Yuan, X., Srividhya, J., De Luca, T., Lee, J.H., and Pomerening, J.R. (2014). Uncovering the role of APC-Cdh1 in generating the dynamics of S-phase onset. Mol. Biol. Cell *25*, 441–456.

Zachariae, W., Schwab, M., Nasmyth, K., and Seufert, W. (1998). Control of cyclin ubiquitination by CDK-regulated binding of Hct1 to the anaphase promoting complex. Science *282*, 1721–1724.

Zetterberg, A., Larsson, O., and Wiman, K.G. (1995). What is the restriction point? Curr. Opin. Cell Biol. *7*, 835–842.

Cell Systems
Supplemental Information

A Dynamical Framework for the All-or-None G1/S Transition

Alexis R. Barr, Frank S. Heldt, Tongli Zhang, Chris Bakal, and Béla Novák

Inventory of Supplemental Information

Figure S1: Validation of GFP-tagged p27^{Kip1}, CyclinE1, and CyclinA2 constructs (related to Figure 1).

Figure S2: Validation of Cdk2 activity sensor, CDK2L-GFP (related to Figure 1).

Figure S3: Dynamics of G1/S regulators in unperturbed cells and upon Cdk2 inhibition (related to Figure 2).

Figure S4: siRNA-mediated depletion of CyclinE1/2 and CyclinA2 (related to Figures 3 and 4, respectively).

Figure S5: siRNA-mediated depletion of Emi1 (related to Figure 5).

Figure S6: Inhibition of Cdk2 and siRNA-mediated depletion of Emi1 (related to Figure 5).

Supplemental Experimental Procedures

Movie S1: p27^{Kip1}-GFP/LSS2-mKate PCNA expressing HeLa cells (related to Figure 1A).
Movie S2: CyclinE1-GFP/LSS2-mKate PCNA expressing HeLa cells (related to Figure 1B).
Movie S3: CyclinA2-GFP/LSS2-mKate PCNA expressing HeLa cells (related to Figure 1C).
Movie S4: CDK2L-GFP/LSS2-mKate PCNA expressing HeLa cells (related to Figure 1D).
Movie S5: GFP-PCNA expressing HeLa cells treated with Control siRNA (related to Figure S6B).
Movie S6: GFP-PCNA expressing HeLa cells treated with Emi1 siRNA (related to Figure S6B).

Supplemental References

Supplemental Figures

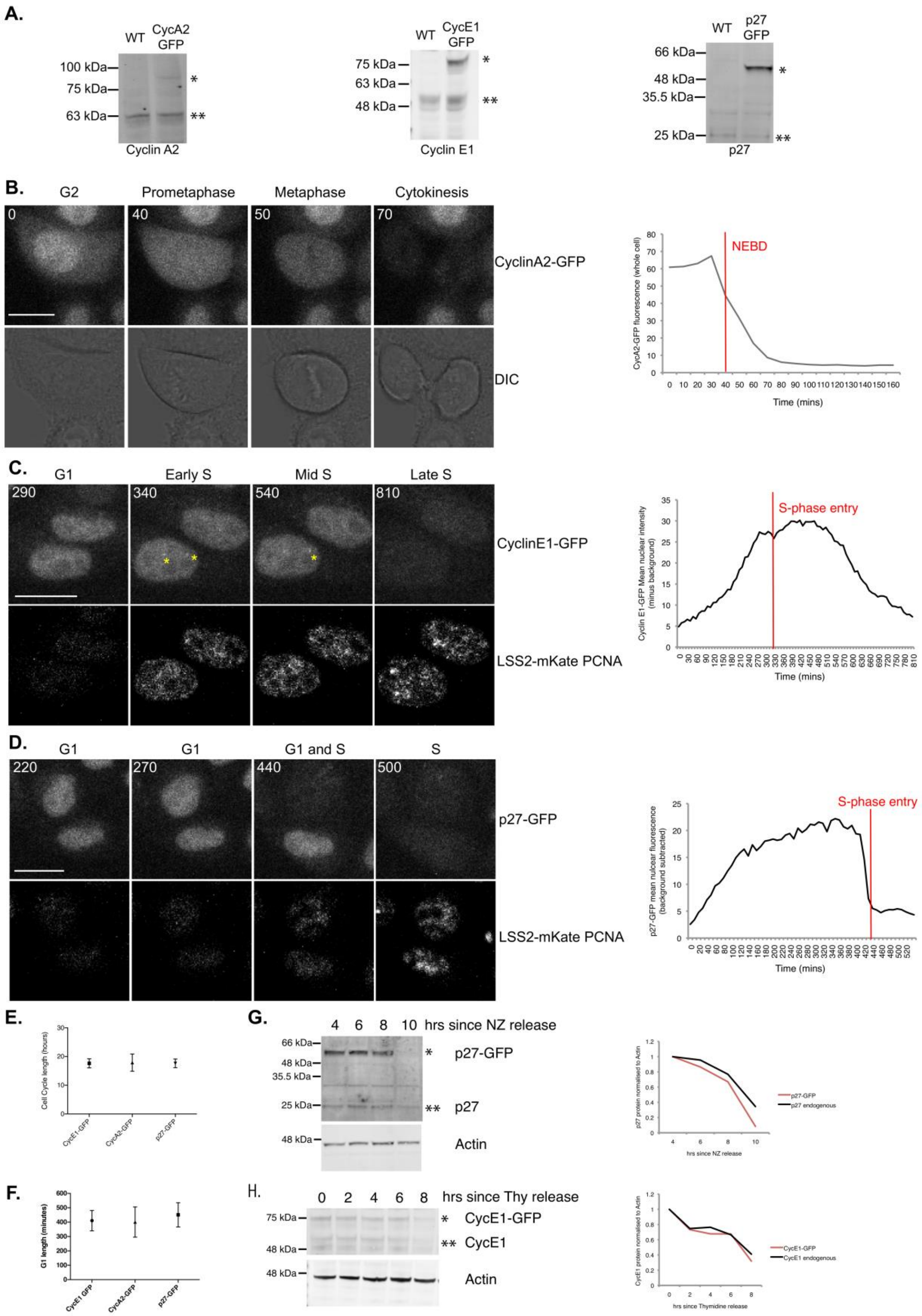

Figure S1: Validation of GFP-tagged p27^{Kip1}, CyclinE1, and CyclinA2 constructs (related to Figure 1). A. Western blots of HeLa clonal cell lines expressing GFP-tagged BAC reporters generated in this study. From left to right: CyclinA2-GFP, CyclinE1-GFP and p27^{Kip1}-GFP. In all three Western blots, wild-type (WT) HeLa lysate is shown on the left and the clonal cell line on the right. The lower bands (marked by double asterisks) are the endogenous proteins and the upper bands (marked by a single asterisk) are the GFP-tagged proteins expressed from the BAC. **B.** CyclinA2-GFP mimics endogenous CyclinA2 localisation and expression: it is stable during S and G2 phases but degraded during mitosis (den Elzen and Pines, 2001). NEBD = nuclear envelope breakdown. Scale bar is 10 μm. Graph represents CyclinA2-GFP expression in a single cell. Relative time is shown in minutes on each image. **C.** CyclinE1-GFP mimics endogenous CyclinE1 localisation and expression in the nucleus and to Cajal bodies (marked by yellow asterisks; (Liu et al., 2000)). CyclinE1-GFP accumulates during G1, and begins to be degraded after cells enter S-phase. CyclinE1-GFP is degraded before the end of S-phase, as reported for endogenous CyclinE1 (Dulic et al., 1992; Koff et al., 1992). Scale bar is 10 μm. Graph represents CyclinE1-GFP expression in a single cell. Relative time is shown in minutes on each image. **D.** p27^{Kip1}-GFP mimics endogenous p27^{Kip1} localisation and expression in the nucleus. p27^{Kip1}-GFP accumulates during G1 and is degraded at S-phase entry, as reported for endogenous p27^{Kip1} (Carrano et al., 1999). Scale bar is 10 μm. Graph represents p27^{Kip1}-GFP expression in a single cell. Relative time is shown in minutes on each image. **E.** Cell cycle length is not significantly different (one-way ANOVA followed by Tukey's multiple comparison test, $p<0.05$) between LSS2-mKate-PCNA sensor expressing cell lines used in this study. Mean +/- standard deviation (STD) is shown. Number of cells analysed: CyclinE1-GFP n=53, CyclinA2-GFP n=58, p27^{Kip1}-GFP n=59, from three independent experiments. **F.** G1 length is significantly longer in p27^{Kip1}-GFP expressing HeLa cells than in CyclinA2-GFP expressing cells (one-way ANOVA, $p<0.05$). However, all p27^{Kip1}-GFP expressing cells progress through G1 successfully and the length of G1 is within the normal range. There is no significant difference in G1 length between CyclinE1-GFP and CyclinA2-GFP, or between CyclinE1-GFP and p27^{Kip1}-GFP cells. Mean +/- STD is shown. Number of cells analysed: CyclinE1-GFP n=52, CyclinA2-GFP n=61, p27^{Kip1}-GFP n=50, from three independent experiments. **G.** Western blot showing degradation of p27^{Kip1}-GFP and endogenous p27^{Kip1} after release from a nocodazole (NZ) block. B-actin was used as a loading control. The lower bands (marked by double asterisk) are the endogenous protein and the upper bands (marked by a single asterisk) are the GFP-tagged protein expressed from the BAC. Graph on the right shows quantification of protein levels over time. For quantification, p27^{Kip1} levels were first normalised to actin and then standardised to an initial p27^{Kip1} level of 1 at 4 hr post NZ release such that the relative rates of degradation could be compared. **H.** Western blot showing degradation of CyclinE1-GFP and endogenous CyclinE1 after release from a double thymidine (Thy) block. B-actin was used as a loading control. The lower bands (marked by double asterisk) are the endogenous protein and the upper bands (marked by a single asterisk) are the GFP-tagged protein expressed from the BAC. Graph on the right shows quantification of protein levels over time. For quantification, CyclinE1 levels were first normalised to actin and then standardised to an initial CyclinE1 level of 1 at 0 hr post Thy release such that the relative rates of degradation could be compared.

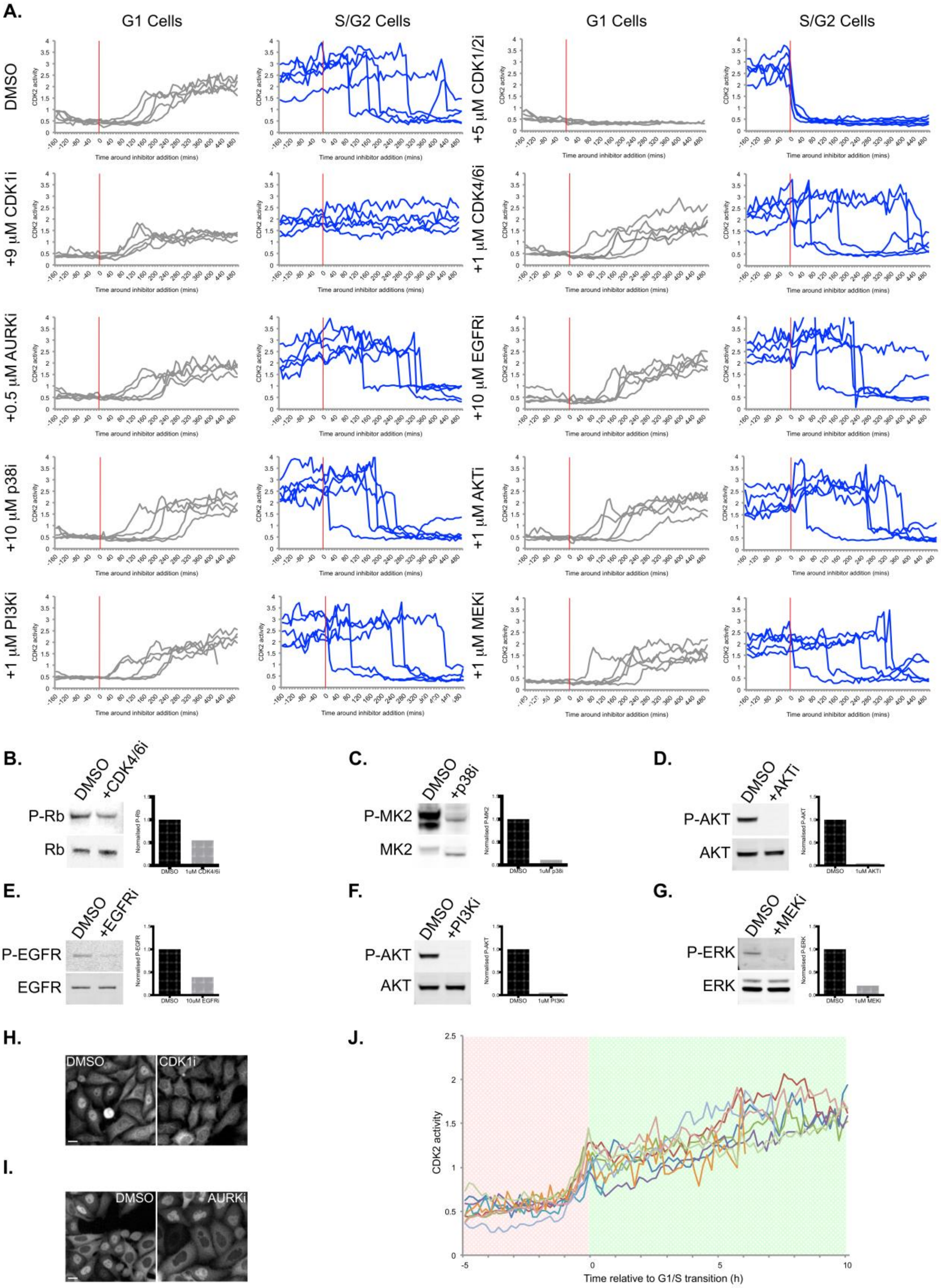
A.
G1 Cells
S/G2 Cells
G1 Cells
S/G2 Cells
DMSO
+9 μM CDK1i
+0.5 μM AURKi
+10 μM p38i
+1 μM PI3Ki
+5 μM CDK1/2i
+1 μM CDK4/6i
+10 μM EGFRi
+1 μM AKTi
+1 μM MEKi
CDK2 activity
Time around inhibitor addition (mins)
B.
DMSO
+CDK4/6i
P-Rb
Rb
Normalised P-Rb
1uM CDK4/6i
C.
+p38i
P-MK2
MK2
Normalised P-MK2
1uM p38i
D.
+AKTi
P-AKT
AKT
Normalised P-AKT
1uM AKTi
E.
+EGFRi
P-EGFR
EGFR
Normalised P-EGFR
10uM EGFRi
F.
+PI3Ki
P-AKT
AKT
Normalised P-AKT
1uM PI3Ki
G.
+MEKi
P-ERK
ERK
Normalised P-ERK
1uM MEKi
H.
DMSO
CDK1i
I.
DMSO
AURKi
J.
CDK2 activity
Time relative to G1/S transition (h)

Figure S2: Validation of Cdk2 activity sensor, CDK2L-GFP (related to Figure 1). A. Graphs show quantification of Cdk2 activity in individual cells around the time of inhibitor addition. For each drug used, two graphs are shown: on the left (grey curves) are cells that were in G1 at the time of drug addition. On the right (blue curves) are cells that were in S or G2 at the time of drug addition. Red vertical line indicates time of drug addition (time 0 mins). Reporter activity only decreases on addition of the CDK1/2 inhibitor. Rapid decreases in Cdk2 activity in other blue curves are cells that are entering mitosis. Note that after CDK1i addition, cells arrest in G2 (blue curves plateau) indicating that the CDK1i is working effectively. Inhibitors used here are: CDK1/2i - CDK1/2 inhibitor III 217714; CDK1i - RO-3306; CDK4/6i - PD-0332991 (Palbociclib); AURKi - CCT241736; EGFRi - Lapatinib; p38i - SB203580; AKTi - MK2206; PI3Ki - GDC-0941; and MEKi - PD-0325901. Each drug was tested in two independent experiments and the results of one experiment are shown here. **B.** Western blot showing reduction in P-S780 pRb after addition of CDK4/6i. Graph shows quantification of Western blot. Note that this Western blot was performed in MCF10A cells to show that the inhibitor is active since Palbociclib has no effect on pRb phosphorylation in HeLa cells. **C.** Western blot showing reduction in P-T334 MAPKAPK2 (MK2) after addition of p38i. Graph shows quantification of Western blot. **D.** Western blot showing reduction in P-S473 AKT after addition of AKTi. Graph shows quantification of Western blot. **E.** Western blot showing reduction in P-Y1068 EGFR after addition of EGFRi. Graph shows quantification of Western blot. **F.** Western blot showing reduction in P-S473 AKT after addition of Pi3Ki. Graph shows quantification of Western blot. **G.** Western blot showing reduction in P-T202/Y204 ERK after addition of ERKi. Graph shows quantification of Western blot. **H.** Images showing arrest in G2 of CDK1i treated cells. Note, in the right hand image that CDK2L-GFP is in the cytoplasm indicating G2 arrest. Scale bar is 10 μm. **I.** Images showing inhibition of cytokinesis in AURKi treated CDK2L-GFP expressing cells. Image panel on right shows irregular shaped nuclei after 24 hr in AURKi. Scale bar is 10 μm. **J.** Graphs shows quantification of individual cells co-expressing the DHB-Ven Cdk2 activity reporter (Spencer et al., 2013) and fluorescent PCNA to mark S-phase entry. Single cell traces are aligned to S-phase entry.

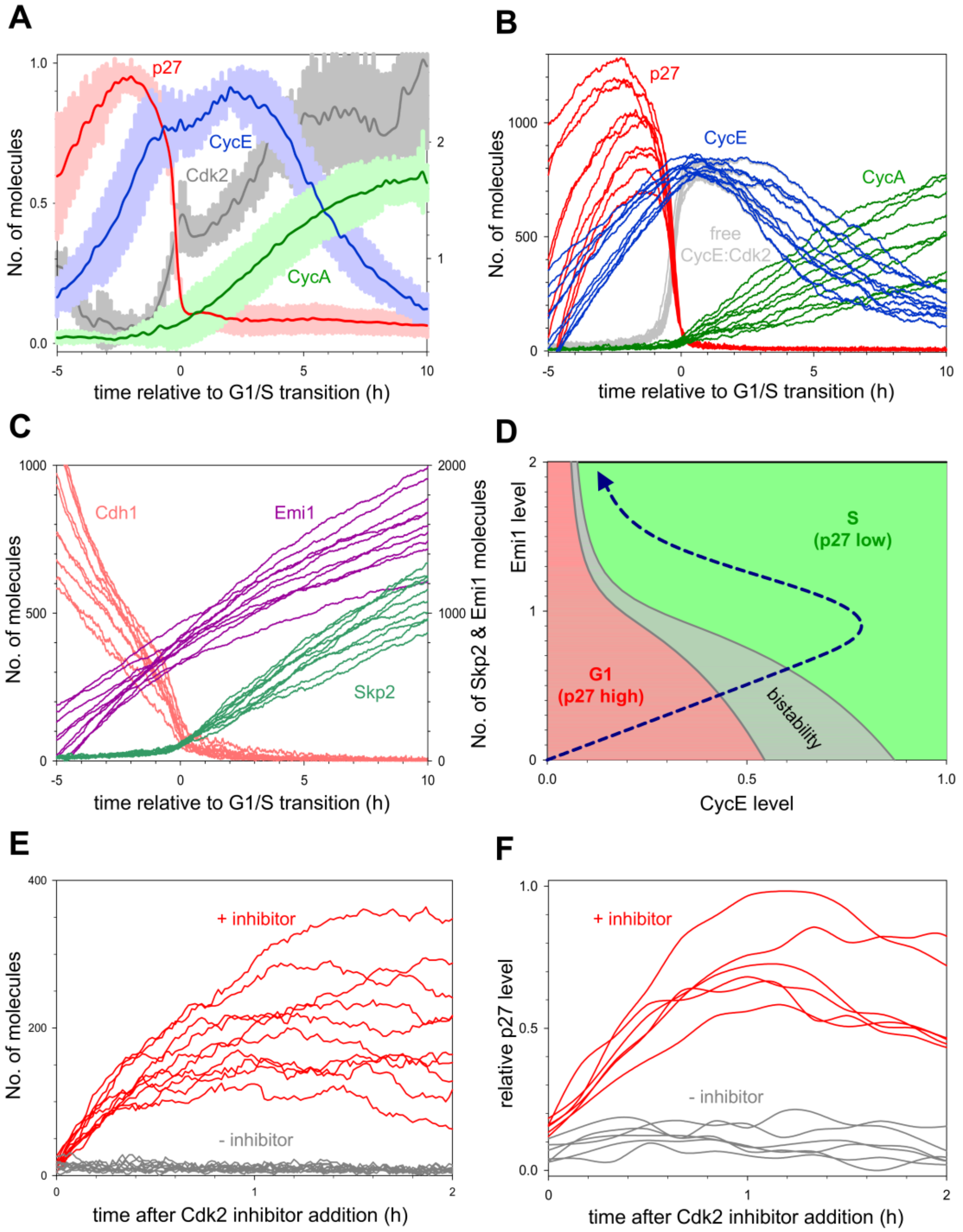

Figure S3: Dynamics of G1/S regulators in unperturbed cells and upon Cdk2 inhibition (related to Figure 2). A. Standard deviation of G1/S aligned and averaged time-courses of experimental measurements of cell cycle reporters and Cdk2 activity, from Fig. 2B. **B.** G1/S aligned stochastic simulations for $p27^{Kip1}$, CyclinE and CyclinA. The curves were aligned to time zero when the number of $p27^{Kip1}$ molecules dropped below 100 (10% of its average peak value). **C.** G1/S aligned stochastic simulations for Emi1, Skp2 and for the active form of Cdh1. **D.** The CyclinE thresholds for $p27^{Kip1}$ inactivation and reactivation from Fig. 2D are plotted as a function of Emi1 levels with the deterministic model. The diagram is divided into three territories: low (green) and high (red) levels of $p27^{Kip1}$ and bistability (grey) where both of these states coexist. The trajectory of cell cycle progression of unperturbed cells is shown by a blue dashed curve. **E**. Stochastic simulations of $p27^{Kip1}$ re-accumulation after Cdk2 inhibition in S-phase arrested cells. Both CyclinE and CyclinA dependent-Cdk2 activities were reduced to 5% at time zero (corresponding to early S-phase). **F**. Quantification of $p27^{Kip1}$-GFP levels after addition of CDK1/2 inhibitor to S-phase arrested cells. $p27^{Kip1}$-GFP accumulates in HeLa cells arrested in S-phase after inhibition of Cdk2 activity. Four independent measurements taken, 6 cells for each treatment from one experiment are shown.

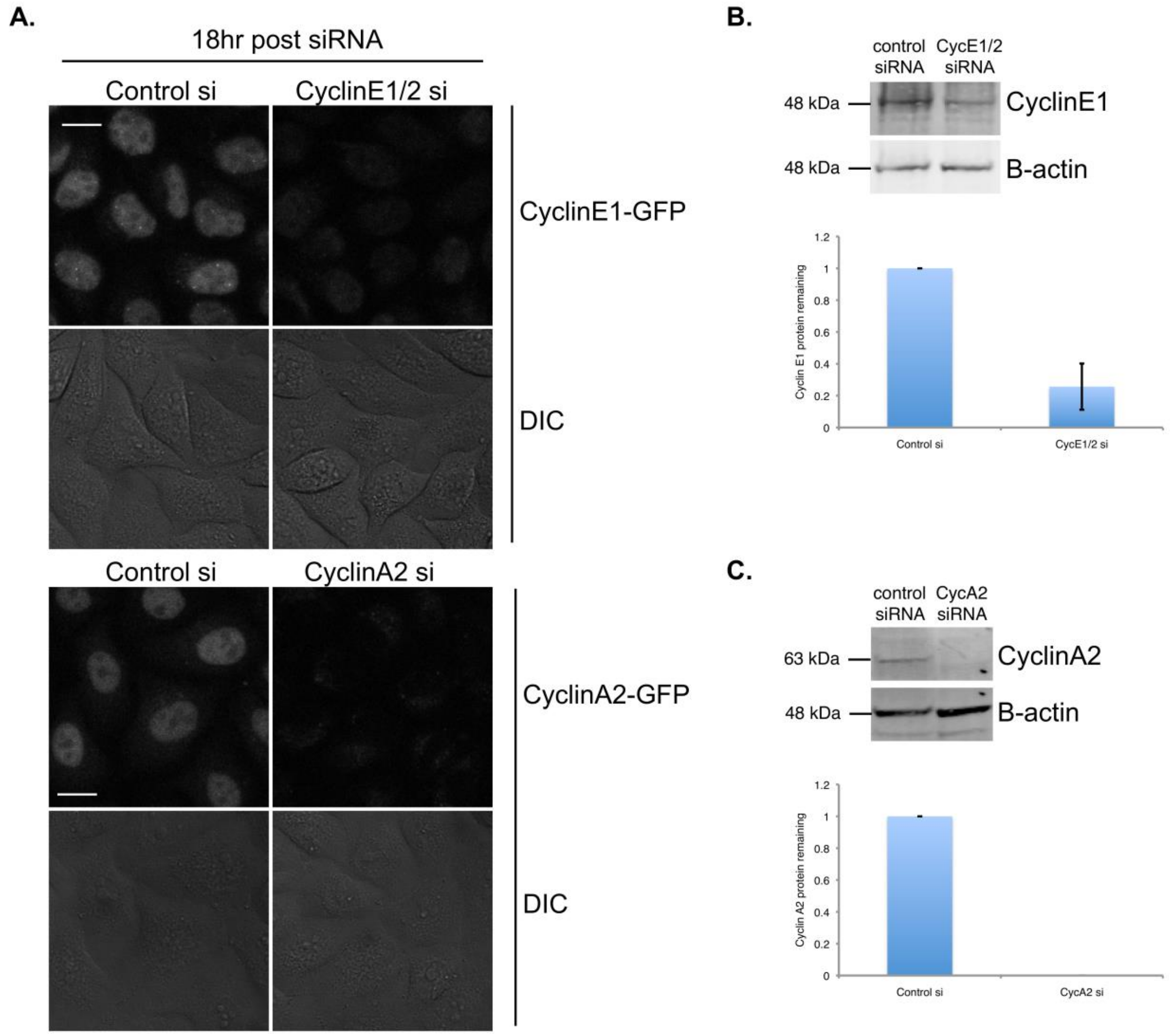

Figure S4: siRNA-mediated depletion of CyclinE1/2 and CyclinA2 (related to Figures 3 and 4). A. Images of CyclinE1-GFP expressing cells treated with CyclinE1/2 siRNA or control siRNA (upper panels) and CyclinA2-GFP expressing cells treated with CyclinA2 siRNA or control siRNA (lower panels) to validate protein knockdown within a single cell cycle. Scale bars are 10 μm. **B.** Representative Western blot for CyclinE1 knockdown. B-actin is used as a loading control. Cells were lysed 18 hr post siRNA transfection (see Supplemental Experimental Procedures). Graph shows quantification of CyclinE1 levels from Western blots, normalised to B-actin. Mean +/- STD is shown (n=4). **C.** Representative Western blot for CyclinA2 knockdown. B-actin is used as a loading control. Cells were lysed 18 hr post siRNA transfection. Graph shows quantification of CyclinA2 levels from Western blots, normalised to B-actin. Mean +/- STD is shown (n=3).

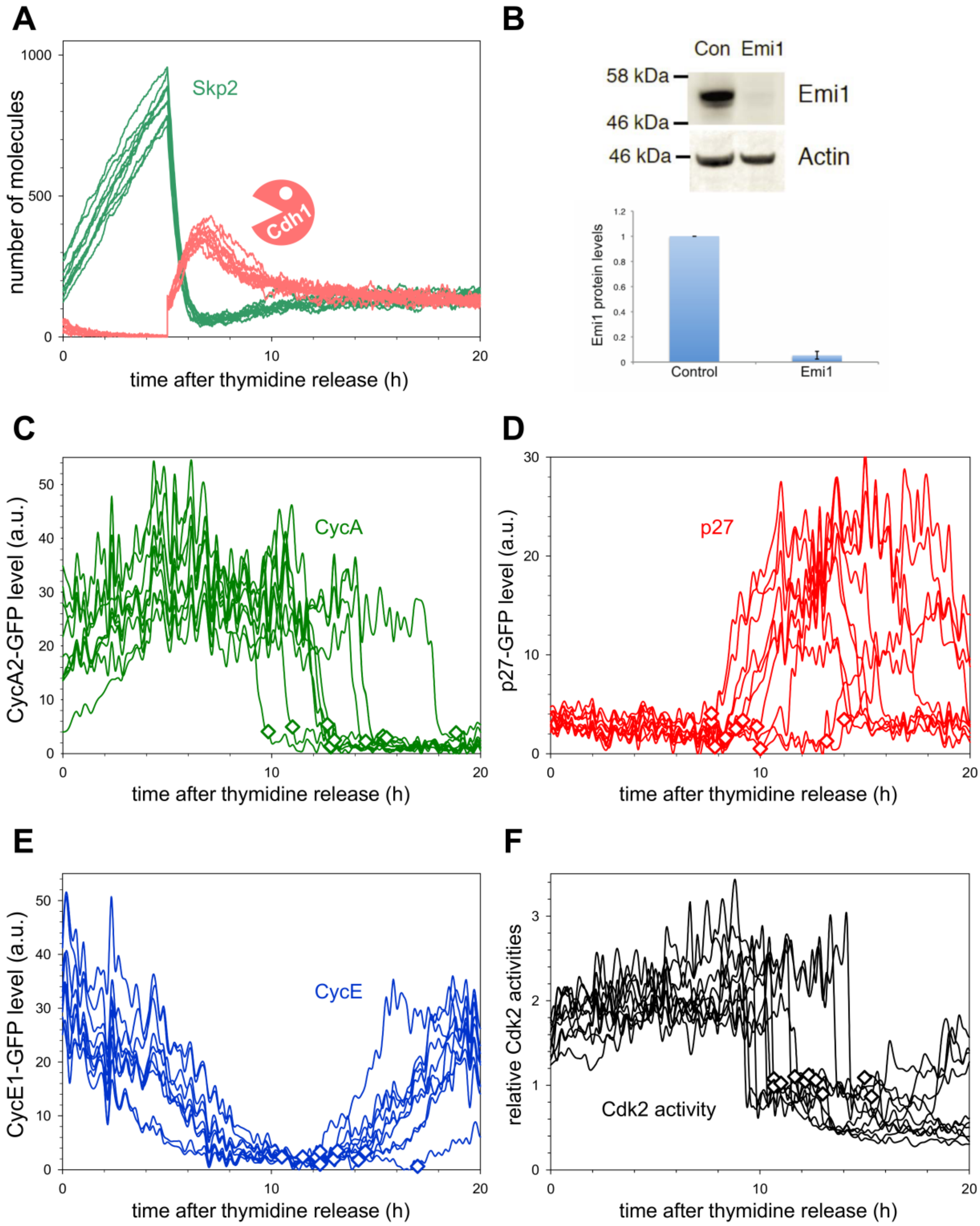

Figure S5: siRNA-mediated depletion of Emi1 (related to Figure 5). A. Stochastic simulations of Emi1 depletion in cells released from a thymidine S-phase block (as in Fig. 5A). Loss of Emi1 (at 5 hr) causes an abrupt reactivation of APC/C^{Cdh1} that promotes degradation of Skp2. **B.** Western blot showing depletion of Emi1 40 hr after the second thymidine release. Graph at the bottom shows quantification of Emi1 depletion. Mean +/- STD is shown (n=2). **C-F.** Quantification of protein/activity levels in cells treated with control siRNA, measured from the time of release from the second thymidine block (corresponds to Fig. 5C-F). Each curve represents a single cell and diamonds represent time of cytokinesis. Note that all control siRNA cells go through mitosis during the filming period. In each graph, 10 cells are shown. At least two independent experiments were conducted in each cell line. **C.** CyclinA2-GFP levels. **D.** p27-GFP levels. **E.** CyclinE1-GFP levels. **F.** Cdk2 activity as measured by the CDK2L-GFP sensor.

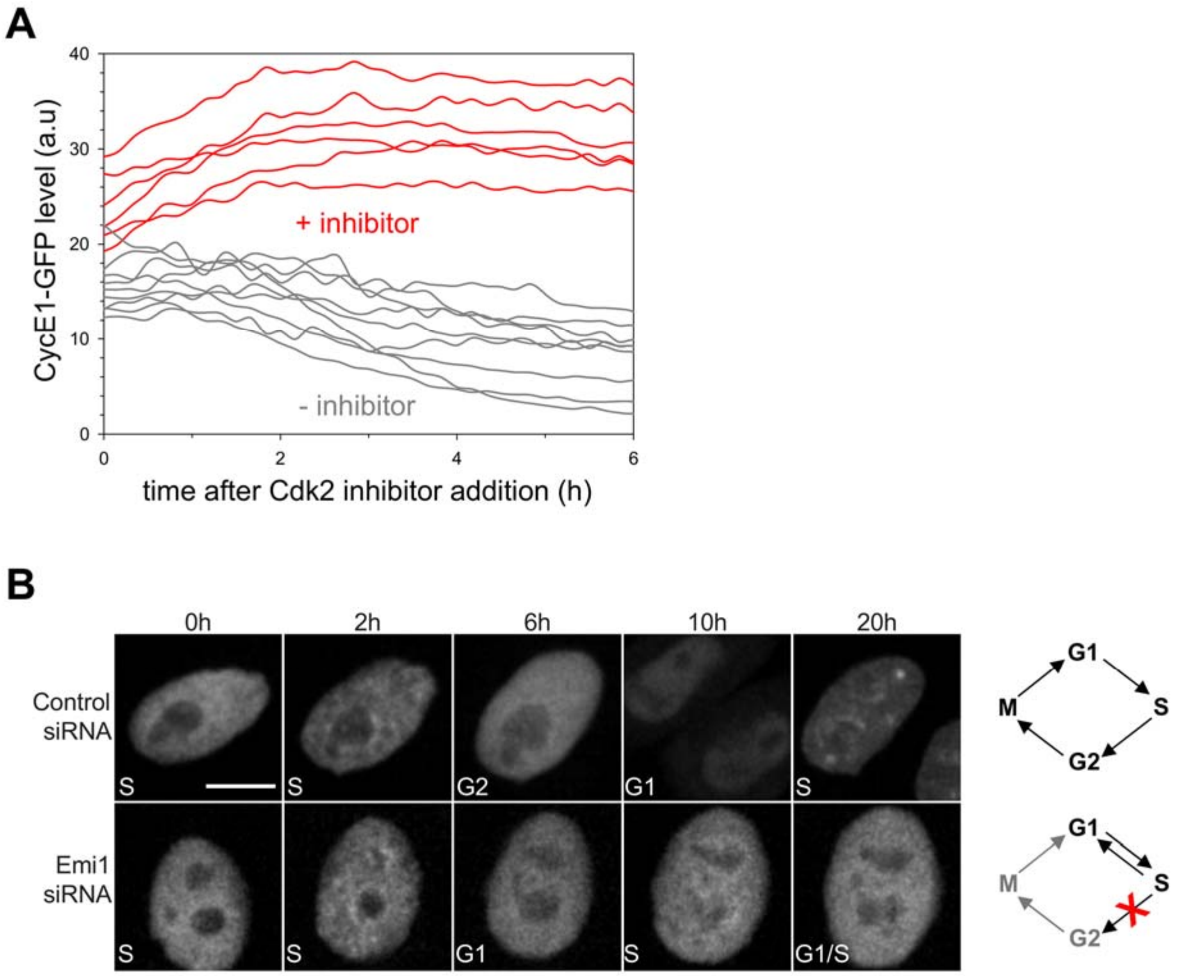

Figure S6: Inhibition of Cdk2 and siRNA-mediated depletion of Emi1 (related to Figure 5). A. Quantification of CyclinE1-GFP protein after addition of CDK1/2 inhibitor to cells in early S-phase (as determined by PCNA foci). CyclinE1-GFP accumulates and is then stabilised after inhibitor addition. Two independent measurements taken, 9 (control) and 6 (inhibitor-treated) cells from one experiment are shown. **B.** Still images taken from Movies S5 and S6 showing expression of fluorescent PCNA in cells treated with Control siRNA (upper panels) or Emi1 siRNA (lower panels). Scale bars are 10 μm. Time 0 hr is release from thymidine block therefore PCNA levels are already high in these cells since they had been arrested in early S-phase. On each panel is the cell cycle stage. The 20h panel for Emi1 siRNA treated cells is labelled as 'G1/S' since cells appear to enter an intermediate state capable of licensing and firing replication origins. On the right are shown the paths through the cell cycle that the cells shown in the images take.

Supplemental Experimental Procedures

Cell culture

HeLa cells (ATCC) were maintained in DMEM (Invitrogen) + 10% FBS (Sigma) and 1% Penicillin/Streptomycin (Invitrogen) at 37°C and 5% CO_2. For live cell imaging, DMEM without phenol red was used.

Generation of reporter cell lines

Tagged BAC constructs were purified using the Nucleobond AX100 kit (Machery-Nagel), according to the manufacturer's protocols for "low-copy plasmid purification". BAC DNA was transfected into HeLa cells using Lipofectamine 2000 (Invitrogen), according to the manufacturer's instructions. The day after transfection, stable clones were selected with 0.4 mg/ml G418 for 7-10 days. After this time, GFP positive cells were sorted to single-cell density in 96-well plates using a FACS Aria (BD Biosciences). Single cell clones were expanded for 7-10 days in selection media and then checked by Western blotting to determine that the full-length protein was tagged. In all cases, the clones with the lowest expression of the GFP-tagged protein were selected. Live-cell imaging was then used to determine that the localisation and dynamics of the tagged protein was the same as described for the endogenous protein.

To monitor PCNA dynamics, we cloned full-length human PCNA cDNA (generous gift of Cristina Cardoso, Technische Universitat Darmstadt) into a modified pIRES-Puro3 vector, where the Puromycin resistance gene had been swapped with a Blasticidin resistance gene. PCNA was tagged at the N-terminus with the modified LSS2-mKate fluorophore to generate LSS2-mKate-PCNA (Piatkevich et al., 2010) (LSS2-mKate cDNA was a generous gift of Vladislav Verkhusha, Albert Einstein College of Medicine, NY). LSS2-mKate-PCNA was transfected into HeLa cell lines using Lipofectamine 2000 (Invitrogen), according to manufacturer's instructions. Stable cell lines were selected over 7-10 days using 0.4 mg/ml G418 and 2 μg/ml Blasticidin (Invitrogen). Single cell clones were then obtained as above, using FACS to sort single cells into 96-well plates. Live-cell imaging was used to determine that the localisation and dynamics of the tagged protein was the same as described for the endogenous protein.

To generate HeLa cells expressing CDK2L-GFP, purified plasmid DNA was transfected into HeLa cells using Lipofectamine 2000 (Invitrogen) according to manufacturer's instructions. Cells were selected in 1 μg/ml Puromycin for 7-10 days and sorted to single cell density, as above, to generate clonal cell lines.

Live Cell Imaging

For imaging on the Zeiss LSM710 confocal microscope, cells were plated in 35 mm uncoated, glass-bottomed Mattek dishes. For imaging on the PerkinElmer Opera high-throughput confocal microscope, cells were plated on 96-well CellCarrier plates (PerkinElmer). Images were captured every 5-10 min and all imaging data was exported as .lsm files (Zeiss) or .flex files (Opera) and imported into Volocity software for analysis and quantification (PerkinElmer).

Degradation time courses

$p27^{Kip1}$-GFP/LSS2-mKate-PCNA cells were arrested in prometaphase by treatment with 40 ng/ml nocodazole for 16 hr. Mitotic shake off was used to collect mitotic cells. Cells were released from this arrest by washing in warm media three times before replating in warm media. Cells were then lysed directly in 1X Laemmli buffer at each timepoint.

CyclinE1-GFP/LSS2-mKate-PCNA cells were arrested in early S-phase by double thymidine synchronisation. After incubation in the second round of thymidine, cells were washed three times in warm PBS and released into warm medium. Cells were then lysed directly in 1X Laemmli buffer at each time-point.

To quantify protein degradation of GFP-tagged and endogenous proteins, each signal was normalised to B-actin at the same time-point. Each normalised value was then scaled to a starting point of 1, such that the degradation of the two proteins could be compared directly on the graph.

Western blotting

Samples for Western blotting were lysed directly in boiling 1X Laemmli buffer. Lysates were separated on 4-20% Precise pre-cast protein gels (Pierce) and transferred to PVDF-FL (Millipore). PVDF membranes were blocked in 5% milk in TBS + 10% glycerol for 1 hr at RT. Membranes were probed with primary antibodies overnight at 4°C. Unbound antibody was removed with three 20 min washes in TBS/0.05% TritonX-100 (TBS/T). Primary antibodies were detected using either anti-mouse DyLight 680 or anti-rabbit DyLight 800 (Cell Signalling) diluted to 1 μg/ml in ROTI-block. Membranes were incubated in the dark for 1 hr at RT in secondary antibodies. Unbound antibody was removed with three 20 min washes in TBS/T. Membranes were

finally washed for 5 min at RT in water before visualising bands on the LiCor imaging system. Protein levels were quantified using Odyssey software and normalised to B-actin, as outlined above. For Emi1 protein detection, anti-mouse HRP secondary antibody was used and proteins were visualised by ECL detection on the Azure digital imaging system.

Primary antibodies used in this study are: CyclinA2 E23.1 (AbCam), CyclinE1 HE12 (AbCam), $p27^{Kip1}$ (BD Bioscience), Emi1 (Zymed), P-S780 pRb (Rb MAb CST C84F6) and Total pRb (Rb MAb CST D20), P-S473 AKT and pan-AKT (CST 9271 and 2920), P-Y1068 EGFR and pan-EGFR (CST 3777 and 2239), P-T202/Y204 ERK and ERK (CST 9106 and 4695), P-T334 MAPKAPK-2 and MAPKAPK-2 (CST 3007 and 12155).

siRNA transfection

All siRNAs were purchased from Dharmacon. Prior to siRNA transfection, cells were washed twice in 1XPBS and incubated in OPTIMEM (Invitrogen). siRNA was transfected into HeLa cells using Lipofectamine RNAiMAX (Invitrogen), according to manufacturer's instructions. siRNA sequences used in this study were: Control siRNA - ON-TARGETplus Non-targeting siRNA #2; CyclinA2 - siGenomePool siRNA, and CyclinE1/E2 – siGenomePool siRNA; Emi1 – custom siRNA Emi1_1 (Di Fiore and Pines, 2007). siRNAs were used at a final concentration of 20 nM.

For synchronisation experiments with CyclinE1/2 and CyclinA2 siRNA, cells were subjected to a double thymidine synchronisation protocol. Briefly, cells were arrested at G1/S with 2 mM Thymidine for 20 hr, released into fresh medium for 9 hr, treated with 2 mM Thymidine a second time for 16 hr, released for 5 hr before cells were transfected with siRNA. After 4 hr incubation in siRNA, cells were imaged to capture cells in the first G1/S transition after protein depletion. We validated that protein was depleted by this time using Western blotting and through visualisation of CyclinE1-GFP or CyclinA2-GFP levels in live cells (Fig. S4A).

For synchronisation experiments with Emi1 siRNA, cells were subjected to a modified double thymidine synchronisation protocol. Cells were arrested at G1/S with 2 mM Thymidine for 20 hr, released into fresh medium for 5 hr then transfected with siRNA. After 4 hr incubation in siRNA cells were again treated with 2 mM Thymidine a second time for 16 hr. Cells were then released form thymidine and filmed immediately or cells were processed for FACS (Fig. 5B) or Western blotting (40 hr after release, Fig. S5B).

Inhibitors

Inhibitors used in this study are: CDK1/2 inhibitor (EMD Biosciences #217714), CDK1 inhibitor RO-3306 (Sigma), CDK4/6 inhibitor (Palbociclib; SelleckChem), Aurora Kinase inhibitor CCT241736 (Bavetsias et al., 2012), EGFR inhibitor (Lapatinib ditosylate, Santa Cruz), AKT inhibitor MK-2206 (SelleckChem), PI3K inhibitor GDC-0941 (SelleckChem), MEK inhibitor PD-0325901 (Sigma), and p38 inhibitor SB203580 (CST). For experiments shown in Fig. S2, a 2X dilution was made of each drug and then either added to cells that were imaged to monitor CDK2 activity or added to cells in a 6-well plate to monitor pathway inhibition by Western blot. Inhibitor was added for 1 hr before cell lysis in all cases. To monitor p38 inhibitor action, we stimulated the cells with sodium arsenite (1/100, Sigma) for the final 10 mins before lysis.

Flow Cytometry

Cells were trypsinised and washed once in cold PBS. Cells were fixed in ice-cold 70% EtOH in PBS at -20 °C overnight. The next day, cells were washed in ice-cold PBS and resuspended in PBS/0.1% Triton X-100 + 20 μg/ml propidium-iodide (Sigma) and 200 μg/ml DNAse-free RNAse A. Cells were stained at RT for 30 min before being stored on ice and analysed on a LSRII (BD Biosciences). FACS data was plotted on FlowJo software.

Mathematical modelling of the G1/S transition

Fig. 2A is our working hypothesis for how Cyclin-dependent kinases and their regulators (e.g. $p27^{Kip1}$, Cdh1) interact to control events of the G1/S transition. The proposed mechanism is based on many reasonable deductions and assumptions drawn from the experimental literature. In order to understand the behaviours of such a complex regulatory network, and to make testable predictions of its emergent properties, we have built a mathematical model of the control system, based on well-established principles of biochemical kinetics, and then used computation to derive precisely the time dependent dynamics of the control network. To this end, the molecular interaction network on Fig. 2A has been converted into a set of ordinary (nonlinear) differential equations (ODE) based on the principle of law of mass action and the following assumptions.

The synthesis and degradation of E2F-independent (p27 and Skp2) and E2F-dependent (CycE, CycA and Emi1) mRNAs and the corresponding proteins are described by the following ODEs:

**mRNA**	**protein**
$\frac{d[p27]_m}{dt} = k^m_{s27} - k^m_{d27} \cdot [p27]_m$	$\frac{d[p27]_T}{dt} = k_{s27} \cdot [p27]_m - V_{dp27} \cdot [p27]_T$
$\frac{d[Skp2]_m}{dt} = k^m_{sskp2} - k^m_{dskp2} \cdot [Skp2]_m$	$\frac{d[Skp2]}{dt} = k_{sskp2} \cdot [Skp2]_m - V_{dskp2} \cdot [Skp2]$
$\frac{d[CycE]_m}{dt} = k^m_{scyce} \cdot [E2F] - k^m_{dcyce} \cdot [CycE]_m$	$\frac{d[CycE]_T}{dt} = k_{scyce} \cdot [CycE]_m - V_{dcyce} \cdot [CycE]_T$
$\frac{d[CycA]_m}{dt} = k^m_{scyca} \cdot [E2F] - k^m_{dcyca} \cdot [CycA]_m$	$\frac{d[CycA]_T}{dt} = k_{scyca} \cdot [CycA]_m - V_{dcyca} \cdot [CycA]_T$
$\frac{d[Emi1]_m}{dt} = k^m_{semi1} \cdot [E2F] - k^m_{demi1} \cdot [Emi1]_m$	$\frac{d[Emi1]_T}{dt} = k_{semi1} \cdot [Emi1]_m - k_{demi1} \cdot [Emi1]_T$

The total levels of proteins existing in more than one form (p27, CycE, CycA and Emi1) are labelled by subscript 'T'. By assuming that the Cyclins associate rapidly with Cdk2 present in excess (Arooz et al., 2000), the total levels of CycE ($CycE_T$) and CycA ($CycA_T$) correspond to the sum of CycE:Cdk2 and CycA:Cdk2 dimers and their trimeric complexes with p27, respectively. We assume that CyclinE accumulates in inactive trimers with Cdk2 and $p27^{Kip1}$ until there is sufficient CyclinE to overcome $p27^{Kip1}$ –mediated inhibition. This assumption is consistent with *in vitro* data describing the binding kinetics of Cdk2:CyclinE and $p27^{Kip1}$, where the transition from a loosely-bound $p27^{Kip1}$ state to a tightly-bound one is fast (1 min^{-1}) compared to a slow off rate (120 min^{-1}; (Sheaff et al., 1997)). This suggests that the vast majority of Cdk2:CyclinE exists in an inhibited trimeric complex during G1 (Swanson et al., 2000), which is consistent with our measurements of Cdk2 activity in HeLa cells.

The first and second order rate constants are indicated by 'k' and both of them have a dimension of $time^{-1}$ because the concentration of molecules is expressed on a relative scale in the deterministic model. Degradation of most proteins is regulated, which is described by specific rate functions ('V'). All rate functions contain a small, first-order rate constant corresponding to slow constitutive degradation in front of regulated degradation terms. The degradation of p27 is promoted by CycE- and CycA-dependent Cdk2 activities and Skp2:

$$V_{dp27} = k_{d27} \; + \; (k_{d27e} \cdot [CycE] + \; k_{d27a} \cdot [CycA]) \cdot [Skp2\;]$$

Auto-phosphorylated CycE becomes a substrate of ubiquitin-dependent proteasomal degradation (Won and Reed, 1996). We assume that CycA-kinase also phosphorylates CycE and thereby promotes its degradation (Kalaszczynska et al., 2009):

$$V_{dcyce} \; = \; k_{dcyce} + \; k_{dcycee} \cdot [CycE] + \; k_{dcycea} \cdot [CycA]$$

The degradation of both CycA and Skp2 are APC/C^{Cdh1}-dependent during G1 phase (Bashir et al., 2004; Wei et al., 2004):

$$V_{dcyca} = \; k_{dcyca} \; + \; k_{dcycac1} \cdot [Cdh1]$$

$$V_{dskp2} = k_{dskp2} \; + \; k_{dskp2c1} \cdot [Cdh1]$$

The p27 stoichiometric Cdk-inhibitor binds reversibly to both CycE:Cdk2 (*CycE*) and CycA:Cdk2 (*CycA*), thereby creating inactive kinase trimers (CycEp27 and CycAp27):

$$\frac{d[CycEp27]}{dt} \; = \; k_{asse} \cdot ([CycE]_T - [CycEp27]) \cdot ([p27]_T - [CycAp27] - [CycEp27]) - (k_{dise} + V_{dp27} + V_{dcyce}) \cdot [CycEp27]$$

$$\frac{d[CycAp27]}{dt} \; = \; k_{assa} \cdot ([CycA]_T - [CycAp27]) \cdot ([p27]_T - [CycAp27] - [CycEp27]) - (k_{disa} + V_{dp27} + V_{dcyca}) \cdot [CycAp27]$$

APC/C^{Cdh1} (labelled as Cdh1) is inactivated by reversible binding of Emi1 (Hsu et al., 2002):

$$\frac{d[EmiC]}{dt} \; = \; k_{asec} \cdot ([Cdh1]_T \; - [\; EmiC]) \cdot ([Emi1]_T \; - \; [EmiC]) \; - \; (k_{diec} \; + \; k_{demi1}) \cdot [EmiC]$$

and by Cdk-dependent phosphorylation (Lukas et al., 1999):

$$\frac{d[Cdh1]_{dp}}{dt} \; = \; k_{acdh1} \cdot ([Cdh1]_T \; - \; [Cdh1]_{dp}) \; - \; V_{icdh1} \cdot [Cdh1]_{dp}$$

where $Cdh1_{dp}$ labels the dephosphorylated forms and

$$V_{icdh1} = k_{icdh1e} \cdot [CycE] + k_{icdh1a} \cdot [CycA]$$

is the rate-function of Cdh1 inactivation by CycE:Cdk2 and CycA:Cdk2.

The only active form of Cdh1 is the dephosphorylated one that is not bound to Emi1:

$$\frac{d[Cdh1]}{dt} = (k_{diec} + k_{demi1}) \cdot \left([Cdh1]_{dp} - [Cdh1]\right) - k_{asec} \cdot [Cdh1] \cdot ([Emi1]_T - [EmiC])$$
$$+ k_{acdh1} \cdot ([Cdh1]_T - [EmiC] - [Cdh1]) - V_{icdh1} \cdot [Cdh1]$$

The levels and the activities of free (not p27 bound) cyclin:Cdk2 complexes are calculated by the following algebraic equations:

$$[CycE] = [CycE]_T - [CycEp27] \quad [CycE]_a = \frac{[CycE]_T - [CycEp27]}{1 + [Inhibitor]}$$

$$[CycA] = [CycA]_T - [CycAp27] \quad [CycA]_a \frac{[CycA]_T - [CycAp27]}{1 + [Inhibitor]}$$

where [Inhibitor] refers to the concentration of the Cdk2 inhibitor relative to its IC_{50} value. It has a value of zero except in the simulations shown in Figure S3E, where its value is 19 which causes a 95% inhibition.

The kinetic parameters of the model (k's) were estimated by numerical simulations of the deterministic model and comparing the calculated trajectories with the pseudo-synchronised average time-courses of cell cycle regulators. The code used with freely available XPPAUT software (http://www.math.pitt.edu/~bard/xpp/xpp.html) is provided below. The values of the kinetic parameters used for simulation of control cells are listed in the XPPAUT code below. For deterministic simulations, the levels of mRNAs were set to one and assumed to be in pseudo-steady state. The initial values of all other dynamic variables were set to zero with the only exceptions of E2F and Cdh1, which were started from a value of one. These initial conditions mimic the state right after cell division, when both Cdh1 and E2F are rapidly reactivated by the APC/C-dependent loss of CycA and CycB (Krek et al., 1994; Sorensen et al., 2001).

The deterministic model has also been used for one- and two-parameter bifurcation diagrams shown on Fig. 2D, Fig. 3B and Fig. S3D.

Stochastic simulations of intrinsic and extrinsic noise were used to capture the non-genetic variability in cell cycle progression of individual HeLa cells. The stochastic fluctuations in the number of mRNA and protein molecules (intrinsic noise) can be accurately simulated by Gillespie's (Gillespie, 2007) Stochastic Simulation Algorithm (SSA). To this end, the rate expressions of individual reactions of the deterministic model were converted into propensity functions. This conversion transforms the relative concentrations (levels) of cell cycle regulators into number of molecules in a given volume. The stochastic fluctuations in the number of mRNA and protein molecules have been followed in a volume of 1000 units using *MatLab* code. Given high copy numbers of cell cycle control proteins, most of the intrinsic noise in cell cycle control has transcriptional origin (Kar et al., 2009). In accordance with sparse experimental data, the probability of transcription and degradation for all mRNAs was set to 0.2 min^{-1} and 0.002 min^{-1}, respectively, which yields about 100 mRNA molecules in steady state (see (Schwanhausser et al., 2011)). Unequal distribution of cell cycle regulators introduces extrinsic noise, which has been simply captured in our stochastic simulations by assuming Gaussian distribution in the number of E2F molecules among individual cells $\sigma_{E2F}^2 = 0.015$.

In case of siRNA-mediated cyclin depletion, the rate constants of the cyclin mRNA degradation were increased 20-fold, which reduced the steady state level of the targeted cyclin's mRNA to 5% of the level in untreated cells.

```
###############################################################################
#           A MATHEMATICAL MODEL FOR G1/S TRANSITION IN HeLa CELLS
###############################################################################

###############################################################################
#    INITITAL CONDITIONS CORRESPONDING TO THE TIME OF CELL DIVISION
initial CycET=0, CycAT=0, p27T=0, CycEp27=0, CycAp27=0
initial Cdh1dp=1, Emi1T=0, EmiC=0, Cdh1=1 Skp2=0

###############################################################################
#    DIFFERENTIAL EQUATIONS
CycET' = kscyce - Vdcyce*CycET
CycAT' = kscyca - Vdcyca*CycAT
p27T' = ks27 - Vdp27*p27T
CycEp27' = kasse*(CycET-CycEp27)*(p27T-CycAp27-CycEp27)-(kdise+Vdp27+Vdcyce)*CycEp27
CycAp27' = kassa*(CycAT-CycAp27)*(p27T-CycAp27-CycEp27)-(kdisa+Vdp27+Vdcyca)*CycAp27
Cdh1dp' = kacdh1*(Cdh1T-Cdh1dp) - Vicdh1*Cdh1dp
Emi1T' = ksemi1 - kdemi1*Emi1T
EmiC' = kasec*(Cdh1T - EmiC)*(Emi1T-EmiC) - (kdiec+kdemi1)*EmiC
Cdh1' = kacdh1*(Cdh1T-EmiC-Cdh1)-Vicdh1*Cdh1-kasec*Cdh1*(Emi1T-EmiC) \
          + (kdiec+kdemi1)*(Cdh1dp-Cdh1)
Skp2' = ksskp2 - (kdskp2 + kdskp2c1*Cdh1)*Skp2

###############################################################################
#    RATE-FUNCTIONS FOR THE DEGRADATION OF p27, CyCE, CyCA
#    AND FOR INACTIVATION OF CDH1 BY Cdk2:CycA/E
Vdp27 = (kd27e*CycE/(1+Inhibitor) + kd27a*CycA/(1+Inhibitor))*Skp2 + kd27
Vdcyca = kdcyca + kdcycac1*Cdh1
Vdcyce = kdcyce + kdcycee*CycE/(1 + Inhibitor) + kdcycea*CycA/(1 + Inhibitor)
Vicdh1 = kicdh1e*CycE/(1+Inhibitor) + kicdh1a*CycA/(1+Inhibitor)

###############################################################################
#     ALGEBRAIC EQUATIONS FOR FREE (NOT p27 BOUND)  Cdk2:CycE/A complexes
CycA = CycAT - CycAp27
CycE = CycET - CycEp27
aux CycA = CycAT - CycAp27
aux CycE = CycET - CycEp27
###############################################################################
#         PARAMETER VALUES
##  CYCE SYNTHESIS, DEGRADATION AND P27 BINDING/DISSOCIATION:
par kscyce=0.003, kdcyce=0.001, kdcycee=0.0001, kdcycea=0.03, kasse=1, kdise=0.02
##  CYCA SYNTHESIS, DEGRADATION AND P27 BINDING/DISSOCIATION:
par kscyca=0.0025, kdcyca=0.002, kdcycac1=0.4, kassa=1, kdisa=0.02
##  P27 SYNTHESIS AND DEGRADATION:
par ks27=0.008, kd27=0.004, kd27e=2, kd27a=2
##  EMI1 SYNTHESIS AND DEGRADATION:
par ksemi1=0.003, kdemi1=0.001
##  CDH1 REGULATION:
par Cdh1T=1, kacdh1=0.02, kicdh1e=0.07, kicdh1a=0.2, kasec=2, kdiec=0.02
##  SKP2 SYNTHESIS AND DEGRADATION:
par ksskp2=0.004, kdskp2=0.002, kdskp2c1=0.2
##  CDK INHIBITOR
par Inhibitor=0
###############################################################################
#        NUMERICAL SETTINGS
@ method=stiff, total=900, bound=100, Maxstore=100000, bound=2000, dt=1, xlo=0, xhi=900
@ ylo=0, yhi=1, NPLOT=5, yp1=p27T, yp2=CycET, yp3=CycAT, yp4=CycE, yp5=cycA
done
```

Supplemental Movies

Movie S1: p27^{Kip1}-GFP/LSS2-mKate PCNA expressing HeLa cells (related to Figure 1A).
From left to right: p27^{Kip1}-GFP, LSS2-mKate PCNA, Brightfield.

Movie S2: CyclinE1-GFP/LSS2-mKate PCNA expressing HeLa cells (related to Figure 1B).
From left to right: CyclinE1-GFP, LSS2-mKate PCNA, Brightfield.

Movie S3: CyclinA2-GFP/LSS2-mKate PCNA expressing HeLa cells (related to Figure 1C).
From left to right: CyclinA2-GFP, LSS2-mKate PCNA, Brightfield.

Movie S4: CDK2L-GFP/LSS2-mKate PCNA expressing HeLa cells (related to Figure 1D).
From left to right: CDK2L-GFP, LSS2-mKate PCNA, Brightfield.

Movie S5: GFP-PCNA expressing HeLa cells treated with Control siRNA (related to Figure S6B).

Movie S6: GFP-PCNA expressing HeLa cells treated with Emi1 siRNA (related to Figure S6B).

Supplemental References

Arooz, T., Yam, C.H., Siu, W.Y., Lau, A., Li, K.K., and Poon, R.Y. (2000). On the concentrations of cyclins and cyclin-dependent kinases in extracts of cultured human cells. Biochemistry *39*, 9494-9501.

Bashir, T., Dorrello, N.V., Amador, V., Guardavaccaro, D., and Pagano, M. (2004). Control of the SCF(Skp2-Cks1) ubiquitin ligase by the APC/C(Cdh1) ubiquitin ligase. Nature *428*, 190-193.

Bavetsias, V., Crumpler, S., Sun, C., Avery, S., Atrash, B., Faisal, A., Moore, A.S., Kosmopoulou, M., Brown, N., Sheldrake, P.W.*, et al.* (2012). Optimization of imidazo[4,5-b]pyridine-based kinase inhibitors: identification of a dual FLT3/Aurora kinase inhibitor as an orally bioavailable preclinical development candidate for the treatment of acute myeloid leukemia. J Med Chem *55*, 8721-8734.

Carrano, A.C., Eytan, E., Hershko, A., and Pagano, M. (1999). SKP2 is required for ubiquitin-mediated degradation of the CDK inhibitor p27. Nat Cell Biol *1*, 193-199.

den Elzen, N., and Pines, J. (2001). Cyclin A is destroyed in prometaphase and can delay chromosome alignment and anaphase. The Journal of cell biology *153*, 121-136.

Di Fiore, B., and Pines, J. (2007). Emi1 is needed to couple DNA replication with mitosis but does not regulate activation of the mitotic APC/C. The Journal of cell biology *177*, 425-437.

Dulic, V., Lees, E., and Reed, S.I. (1992). Association of human cyclin E with a periodic G1-S phase protein kinase. Science *257*, 1958-1961.

Gillespie, D.T. (2007). Stochastic simulation of chemical kinetics. Annu Rev Phys Chem *58*, 35-55.

Hsu, J.Y., Reimann, J.D., Sorensen, C.S., Lukas, J., and Jackson, P.K. (2002). E2F-dependent accumulation of hEmi1 regulates S phase entry by inhibiting APC(Cdh1). Nat Cell Biol *4*, 358-366.

Kalaszczynska, I., Geng, Y., Iino, T., Mizuno, S., Choi, Y., Kondratiuk, I., Silver, D.P., Wolgemuth, D.J., Akashi, K., and Sicinski, P. (2009). Cyclin A is redundant in fibroblasts but essential in hematopoietic and embryonic stem cells. Cell *138*, 352-365.

Kar, S., Baumann, W.T., Paul, M.R., and Tyson, J.J. (2009). Exploring the roles of noise in the eukaryotic cell cycle. Proceedings of the National Academy of Sciences of the United States of America *106*, 6471-6476.

Koff, A., Giordano, A., Desai, D., Yamashita, K., Harper, J.W., Elledge, S., Nishimoto, T., Morgan, D.O., Franza, B.R., and Roberts, J.M. (1992). Formation and activation of a cyclin E-cdk2 complex during the G1 phase of the human cell cycle. Science *257*, 1689-1694.

Krek, W., Ewen, M.E., Shirodkar, S., Arany, Z., Kaelin, W.G., Jr., and Livingston, D.M. (1994). Negative regulation of the growth-promoting transcription factor E2F-1 by a stably bound cyclin A-dependent protein kinase. Cell *78*, 161-172.

Liu, J., Hebert, M.D., Ye, Y., Templeton, D.J., Kung, H., and Matera, A.G. (2000). Cell cycle-dependent localization of the CDK2-cyclin E complex in Cajal (coiled) bodies. Journal of cell science *113 (Pt 9)*, 1543-1552.

Lukas, C., Sorensen, C.S., Kramer, E., Santoni-Rugiu, E., Lindeneg, C., Peters, J.M., Bartek, J., and Lukas, J. (1999). Accumulation of cyclin B1 requires E2F and cyclin-A-dependent rearrangement of the anaphase-promoting complex. Nature *401*, 815-818.

Piatkevich, K.D., Hulit, J., Subach, O.M., Wu, B., Abdulla, A., Segall, J.E., and Verkhusha, V.V. (2010). Monomeric red fluorescent proteins with a large Stokes shift. Proceedings of the National Academy of Sciences of the United States of America *107*, 5369-5374.

Schwanhausser, B., Busse, D., Li, N., Dittmar, G., Schuchhardt, J., Wolf, J., Chen, W., and Selbach, M. (2011). Global quantification of mammalian gene expression control. Nature *473*, 337-342.

Sheaff, R.J., Groudine, M., Gordon, M., Roberts, J.M., and Clurman, B.E. (1997). Cyclin E-CDK2 is a regulator of p27Kip1. Genes Dev *11*, 1464-1478.

Sorensen, C.S., Lukas, C., Kramer, E.R., Peters, J.M., Bartek, J., and Lukas, J. (2001). A conserved cyclin-binding domain determines functional interplay between anaphase-promoting complex-Cdh1 and cyclin A-Cdk2 during cell cycle progression. Molecular and cellular biology *21*, 3692-3703.

Spencer, S.L., Cappell, S.D., Tsai, F.C., Overton, K.W., Wang, C.L., and Meyer, T. (2013). The proliferation-quiescence decision is controlled by a bifurcation in CDK2 activity at mitotic exit. Cell *155*, 369-383.

Swanson, C., Ross, J., and Jackson, P.K. (2000). Nuclear accumulation of cyclin E/Cdk2 triggers a concentration-dependent switch for the destruction of p27Xic1. Proceedings of the National Academy of Sciences of the United States of America *97*, 7796-7801.

Wei, W., Ayad, N.G., Wan, Y., Zhang, G.J., Kirschner, M.W., and Kaelin, W.G., Jr. (2004). Degradation of the SCF component Skp2 in cell-cycle phase G1 by the anaphase-promoting complex. Nature *428*, 194-198.

Won, K.A., and Reed, S.I. (1996). Activation of cyclin E/CDK2 is coupled to site-specific autophosphorylation and ubiquitin-dependent degradation of cyclin E. The EMBO journal *15*, 4182-4193.
